# Supplementary material for: PlantCAD2: A DNA foundation model for interpreting genomes across flowering plants
Source: Cell Genom. 2026 Aug 7;6(9):101329. doi: 10.1016/j.xgen.2026.101329 (PMC13576720; doi:10.1016/j.xgen.2026.101329)
Supplement: Supplementary file 1 [file mmc1.pdf]

**Cell Genomics, Volume *6***

## Supplemental information

# PlantCAD2: A DNA foundation model

# for interpreting genomes across flowering plants

**Jingjing Zhai, Aaron Gokaslan, Sheng-Kai Hsu, Szu-Ping Chen, Zong-Yan Liu, Edgar Marroquin, Eric Czech, Betsy Cannon, Ana Berthel, M. Cinta Romay, Matt Pennell, Volodymyr Kuleshov, and Edward S. Buckler**

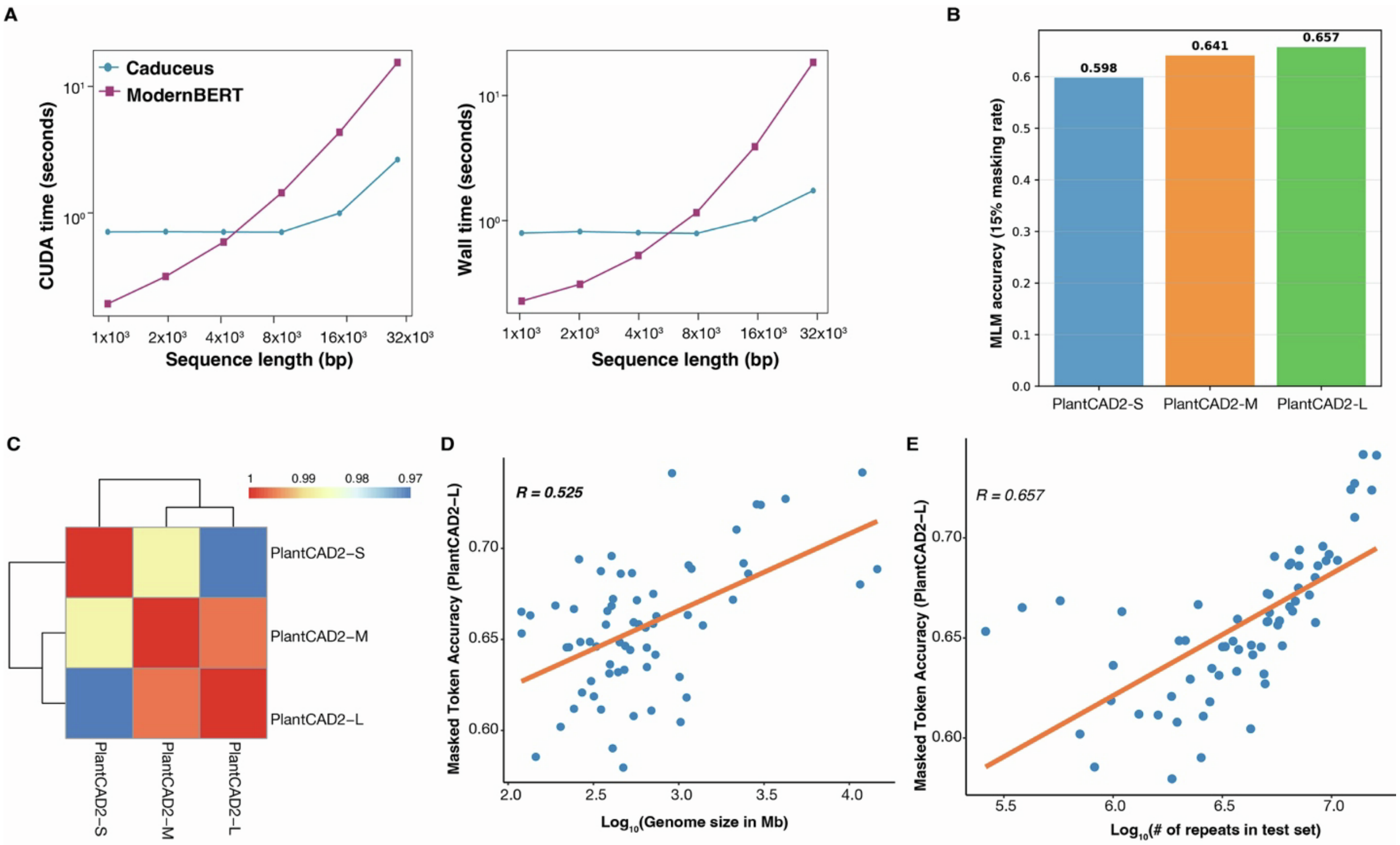


**Figure S1. PlantCAD2 model performance across different scales and species, related to Figure 1. (A)** Inference speed benchmarking shows that the Caduceus architecture exhibits significantly faster CUDA and wall-clock runtime than ModernBERT across increasing sequence lengths. **(B)** Masked language modeling accuracy across the three models, evaluated by randomly masking 15% of nucleotides per sequence, showing improved performance with increasing model size. **(C)** Correlation matrix of per-species masked language modeling accuracy of three PlantCAD2 models. **(D)** Relationship between genome size and masked language modeling accuracy for PlantCAD2-L. **(E)** Relationship between the number of repetitive sequences in the test set and masked language modeling accuracy.

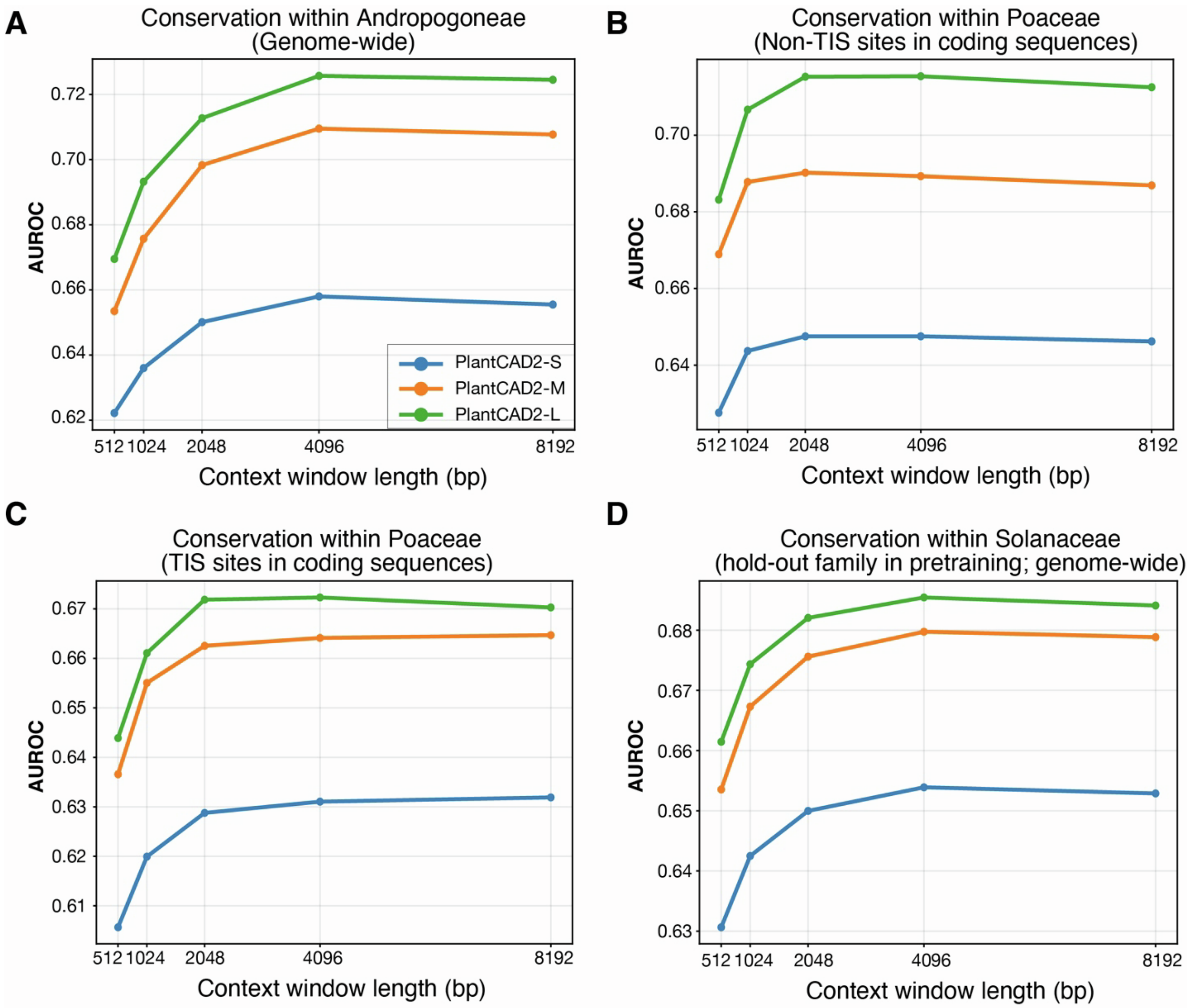


**Figure S2. Effect of context window length on AUROC performance, related to Figure 2.** Evaluation across three PlantCAD2 models for conservation within Andropogoneae **(A)**, Poaceae non-TIS **(B)**, Poaceae TIS **(C)**, and Solanaceae **(D)**.

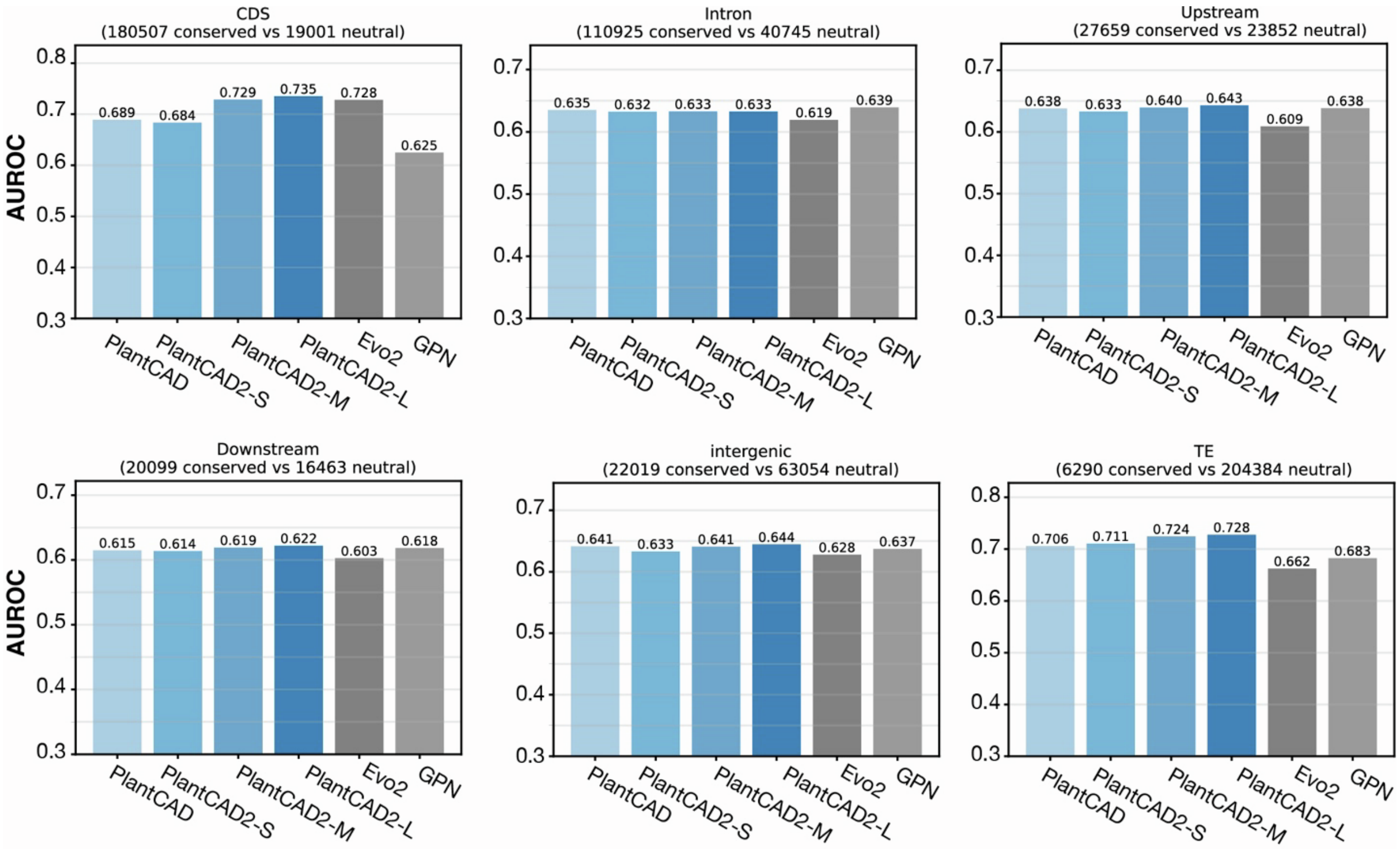


**Figure S3. AUROC of different genomic contexts for conservation prediction in Solanaceae, related to Figure 2.**

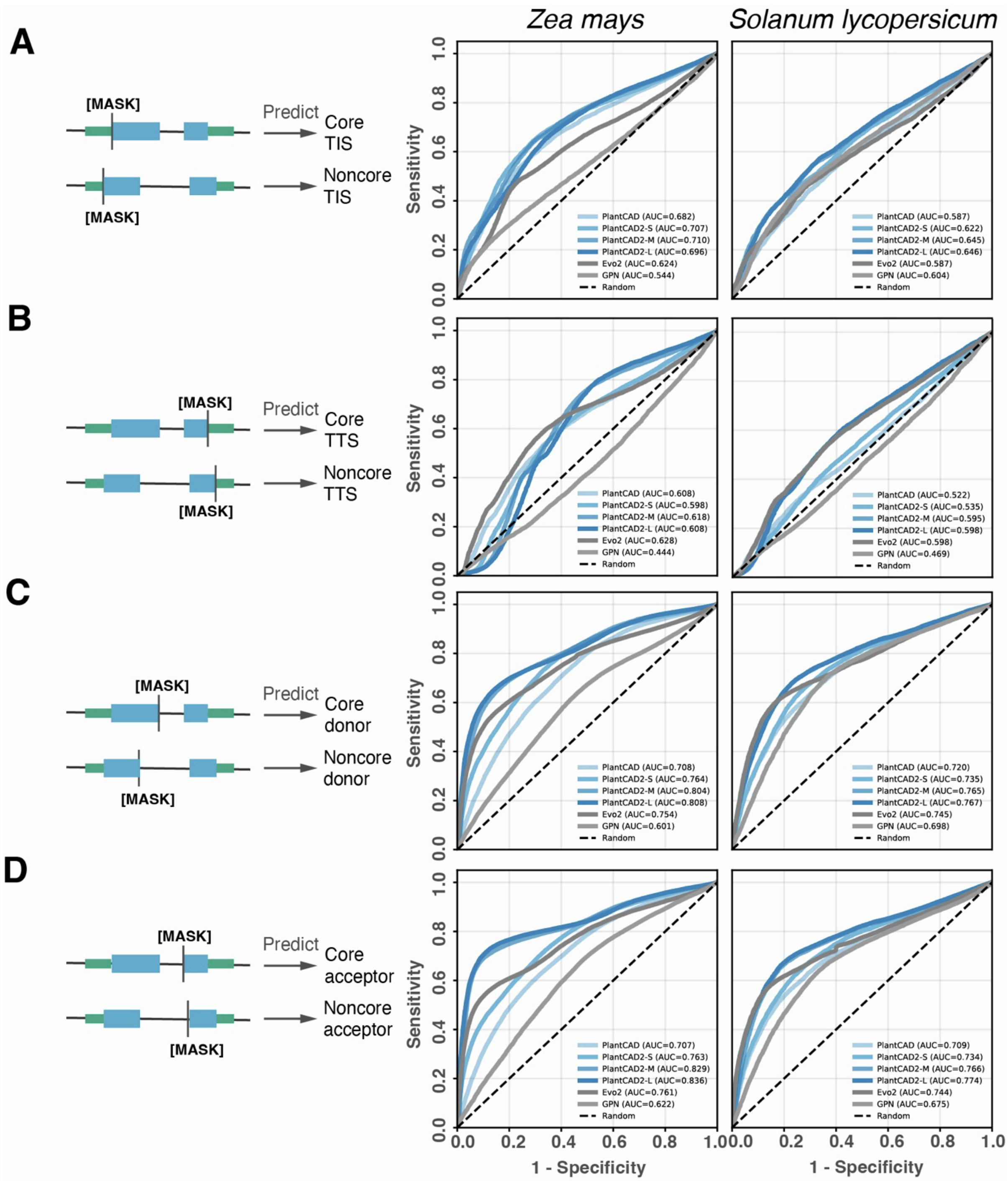


**Figure S4. PlantCAD2 distinguishes core from non-core junctions with zero-shot strategy, related to Figure 3.** ROC curves showing binary classification performance for distinguishing evolutionarily constrained core junctions from rapidly evolving non-core junctions using model log-likelihood scores at junction sites. Left panels show maize (included in pre-training), right panels show tomato (excluded from training). **(A)** Translation initiation sites. **(B)** Translation termination sites. **(C)** Splice donor sites. **(D)** Splice acceptor sites.

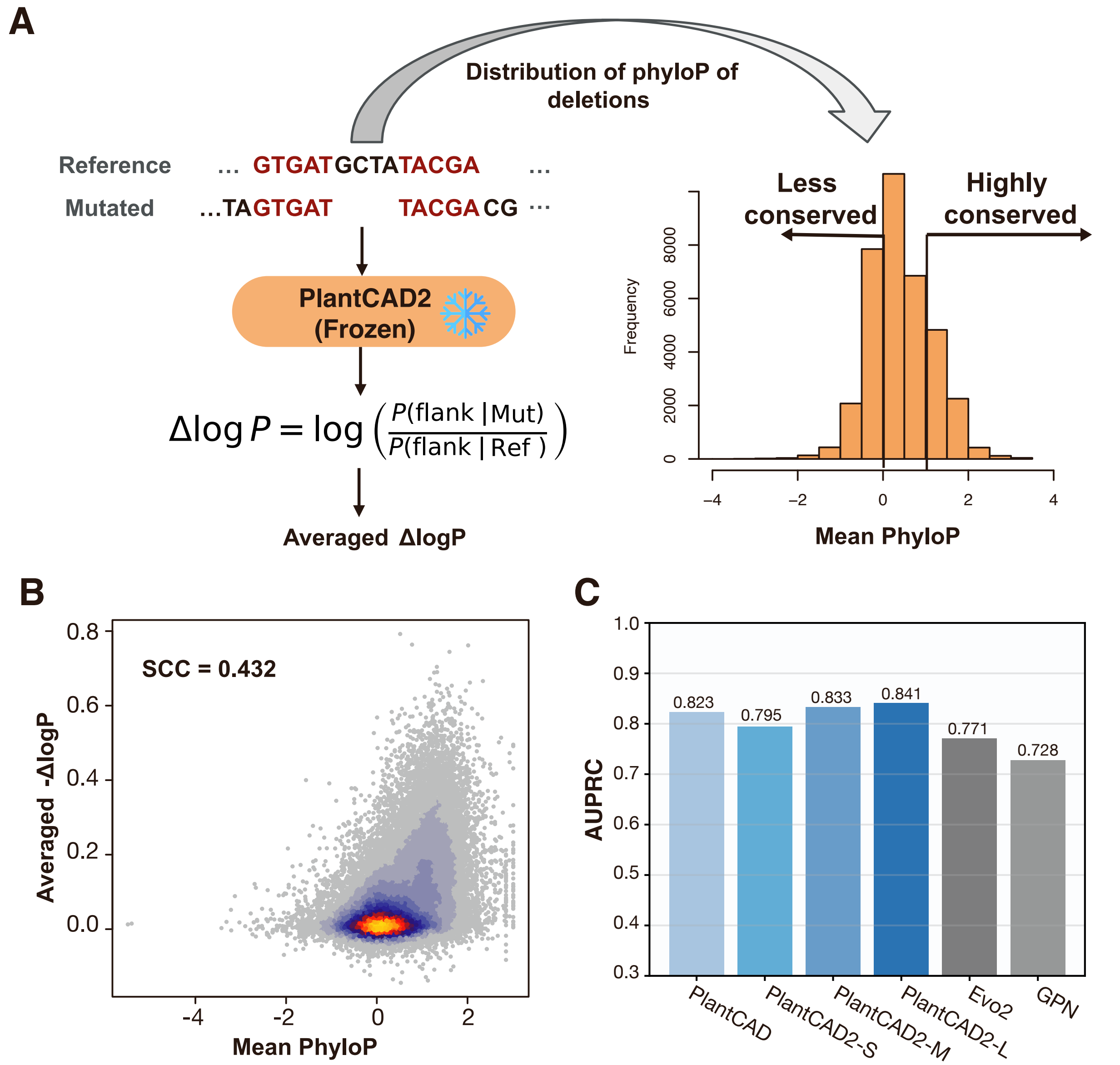


**Figure S5. PlantCAD2 predicts functional impact of structural variants using zero-shot strategy, related to Table 1. (A)** ΔlogP calculation approach for deletion variants and phyloP score distribution for classification. **(B)** Scatter plot showing the positive correlation between PlantCAD2's -ΔlogP scores and phyloP-based conservation scores. **(C)** AUROC performance distinguishes highly conserved from less conserved deletions.

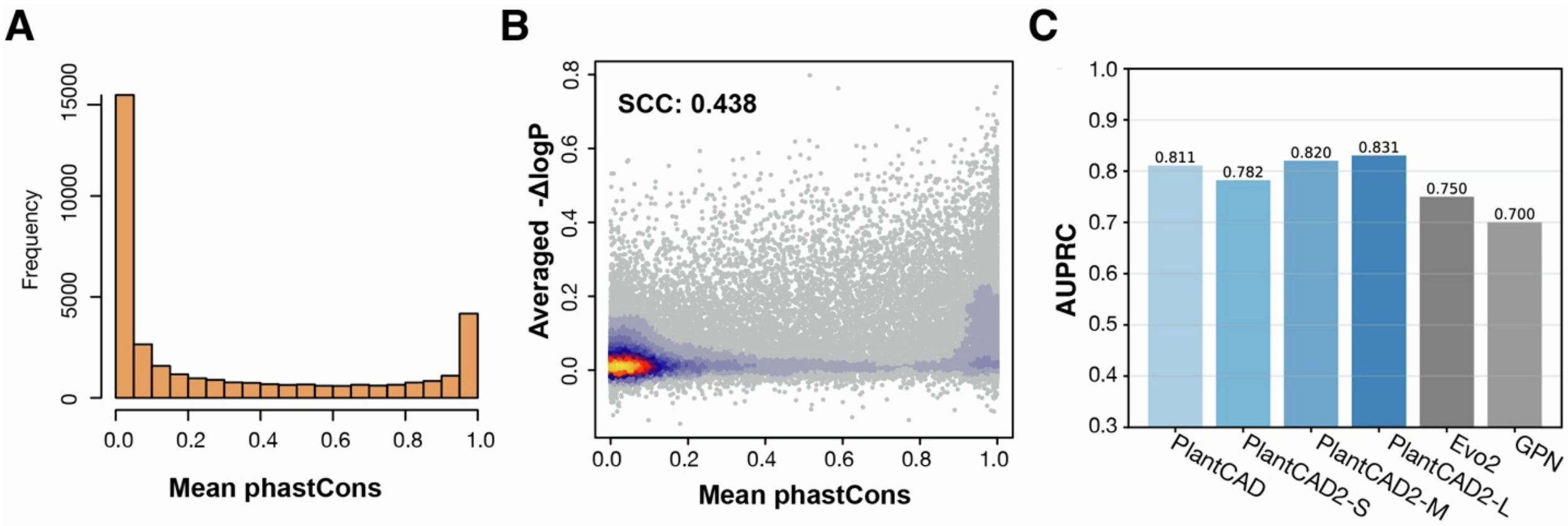


**Figure S6. PlantCAD2 predicts functional impact of structural variants using phastCons scores, related to Table 1.** (A) Distribution of phastCons. (B) Scatter plot showing the positive correlation between PlantCAD2's -ΔlogP scores and phyloP-based conservation scores. (C) AUROC performance distinguishes highly conserved from less conserved deletions.

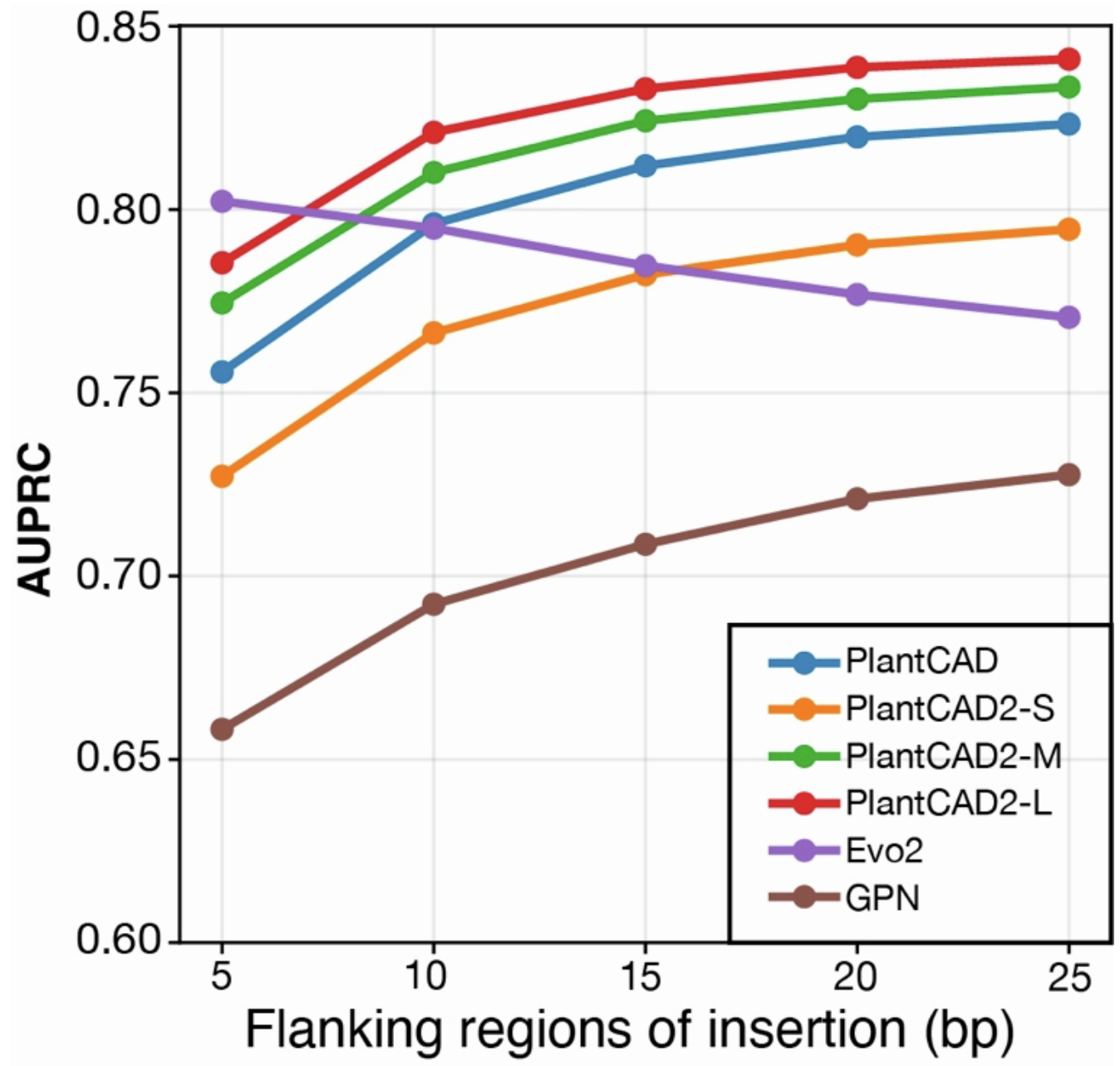


**Figure S7. Effect of flanking sequence length on deletion classification performance, related to Table 1.** AUROC performance for distinguishing conserved from less conserved deletions by flanking sequence length.

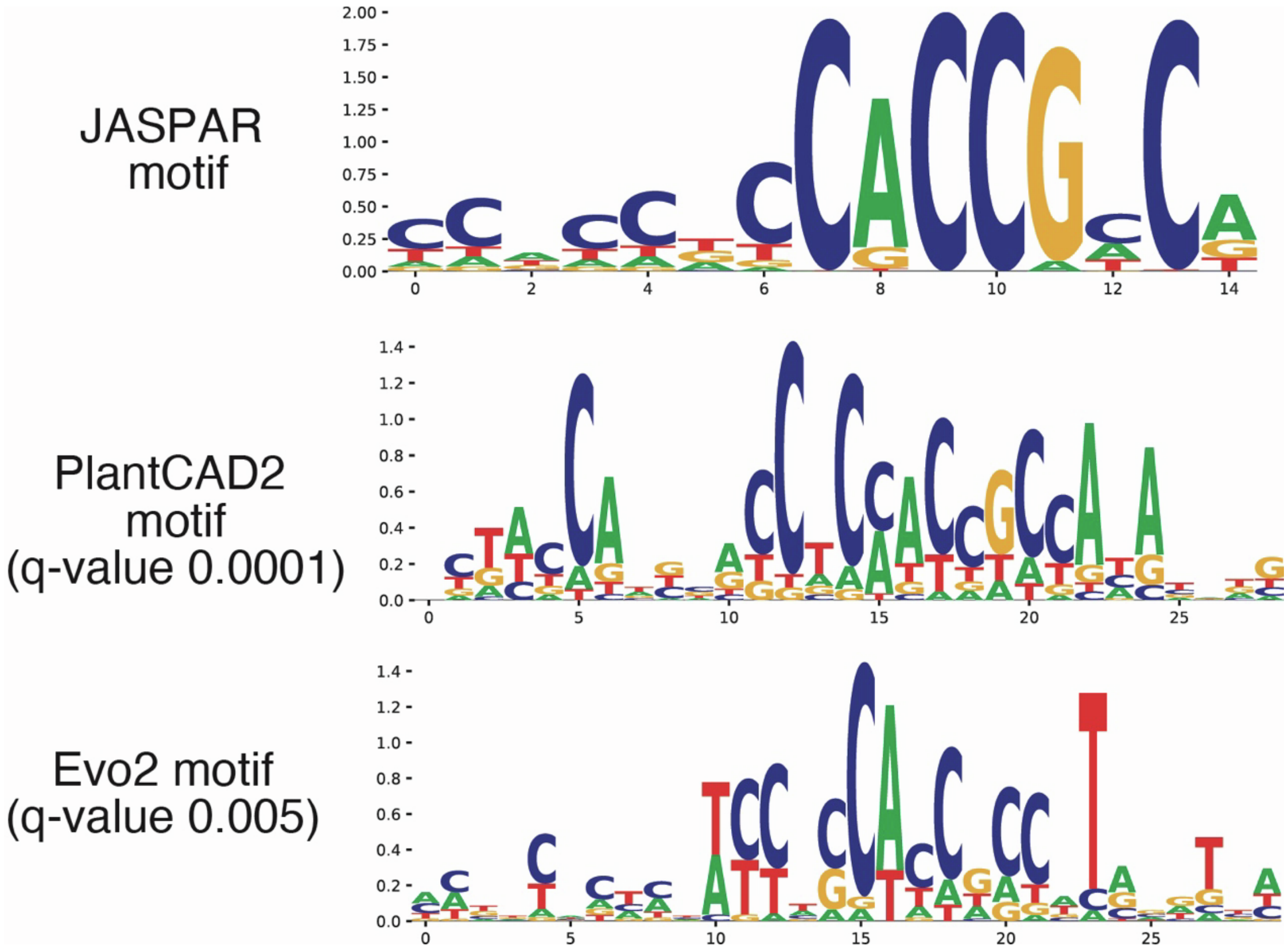


**Figure S8. Motifs generated by TF-MoDISco from PlantCAD2 and Evo2 high-confidence predictions for DREB2 (AP2/ERF family), related to STAR Methods.**

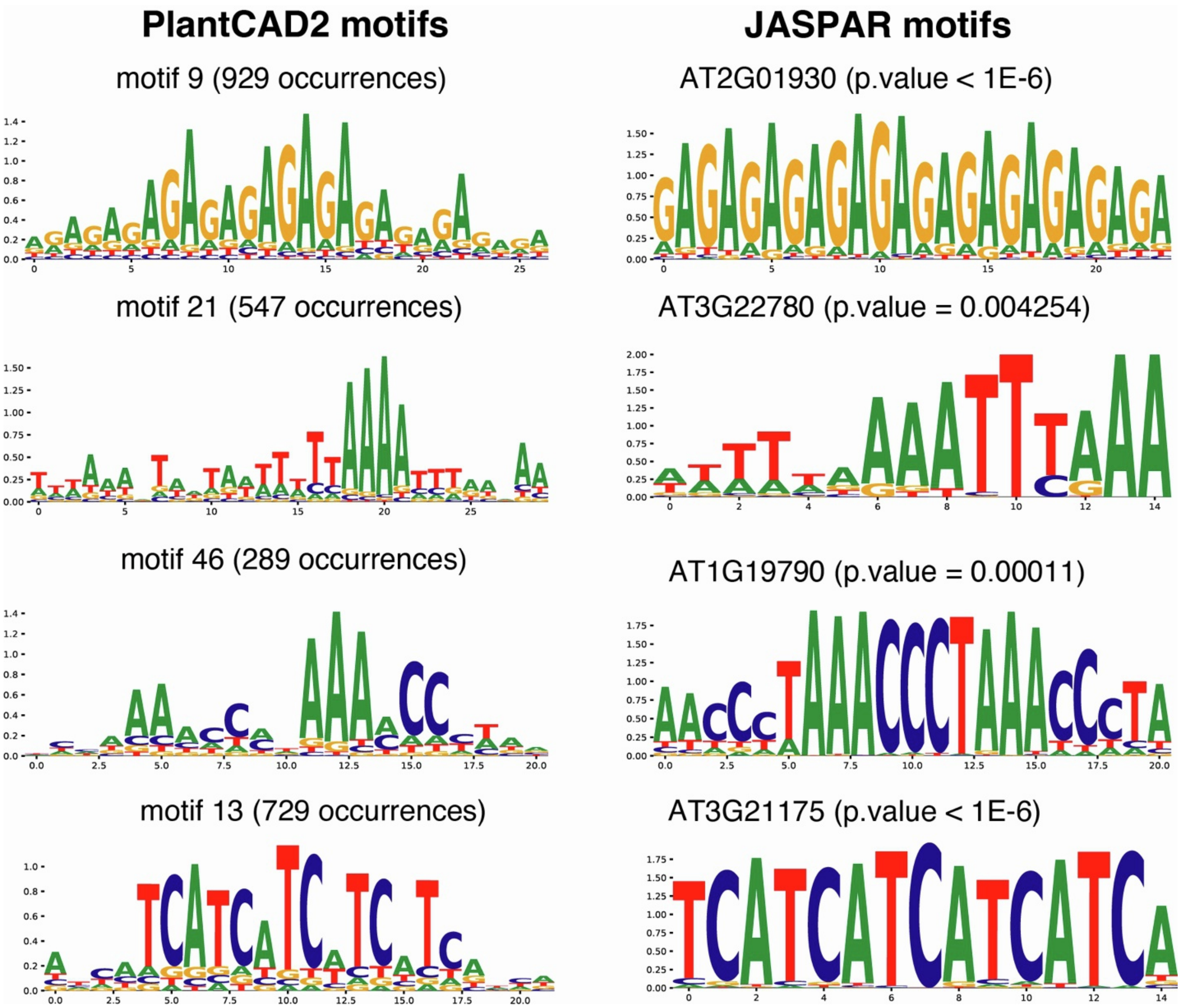


**Figure S9. Example motifs generated by TF-MoDISco from PlantCAD2 high-confidence predictions in Arabidopsis promoter regions, related to STAR Methods.**

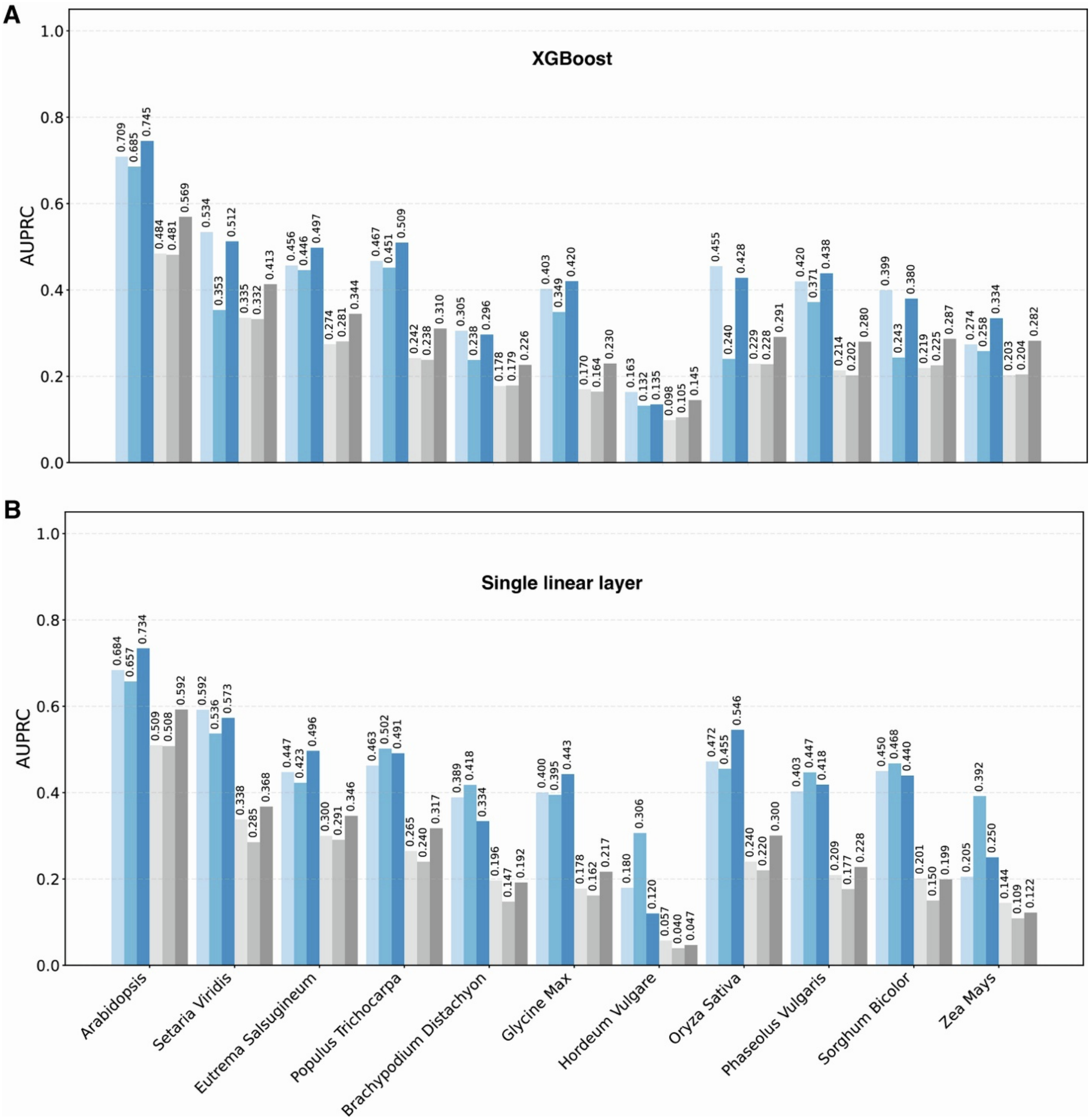


**Figure S10. Accessible region prediction task comparison with Evo2 using frozen embeddings, related to Figure 4. (A)** XGBoost classifier**, (B)** neural network with single linear layer.

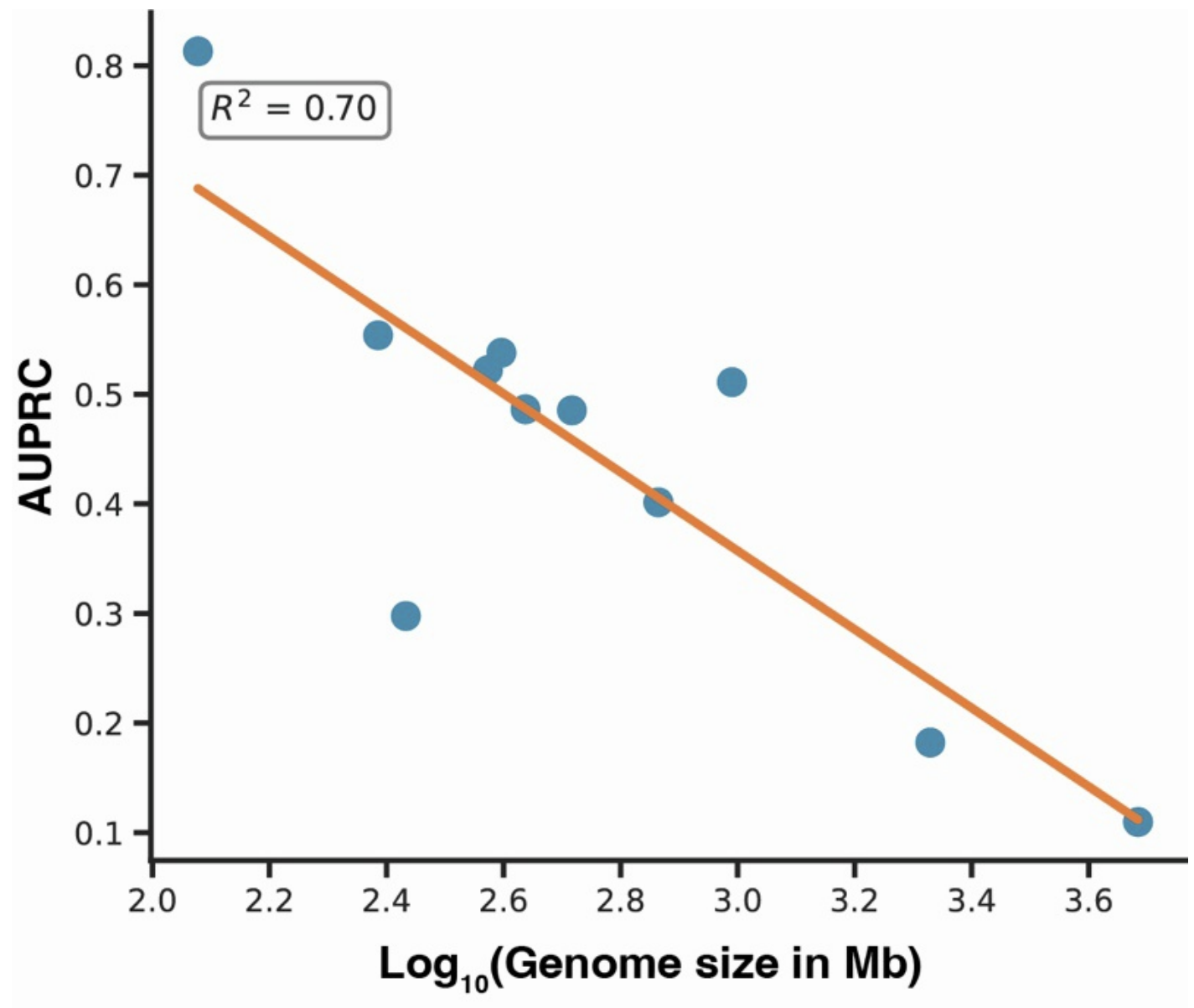


**Figure S11.** Relationship between genome size and accessibility prediction performance, related to Figure 4.

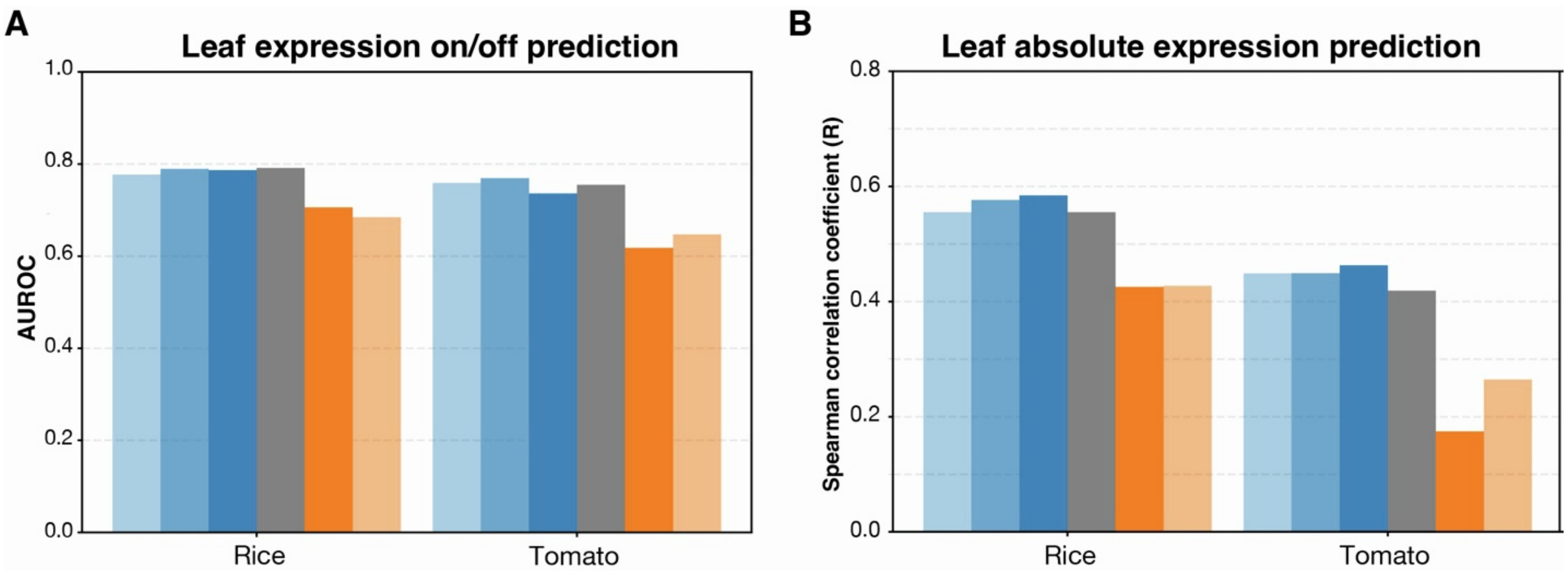


**Figure S12. Gene expression prediction performance in rice and tomato, related to Figure 5.** **(A)** Leaf expression on/off prediction**, (B)** leaf absolute expression prediction.

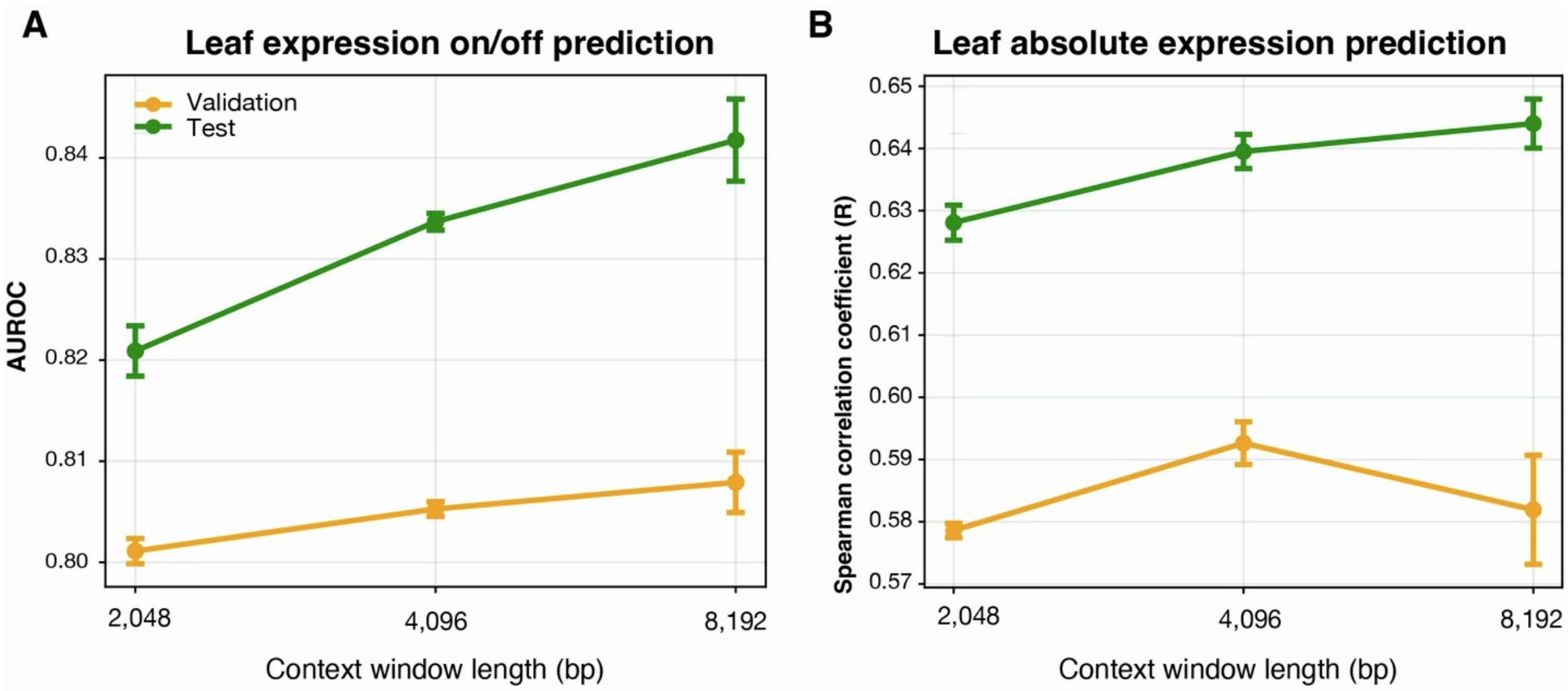


**Figure S13. Effect of context window length on gene expression prediction performance, related to Figure 5.** Performance improvement with increasing context window size for binary leaf expression classification **(A)** and absolute expression regression **(B)** on the maize NAM dataset. Error bars represent five replicates with different random seeds.

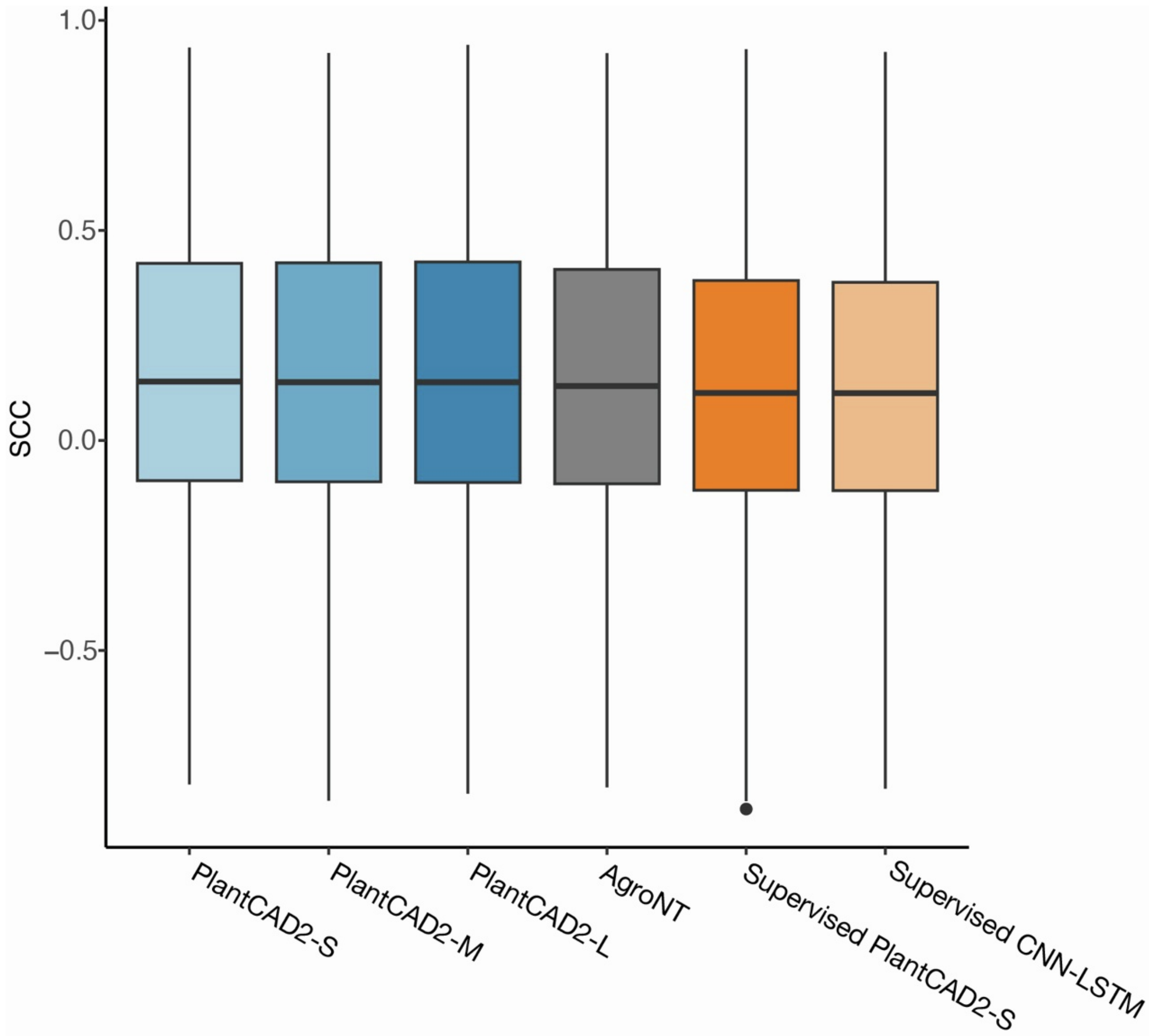


**Figure S14. Per-orthogroup expression correlation performance within the maize NAM population, related to Figure 5.** Spearman correlation coefficients (SCC) for predicting allele-specific expression differences within orthogroups.

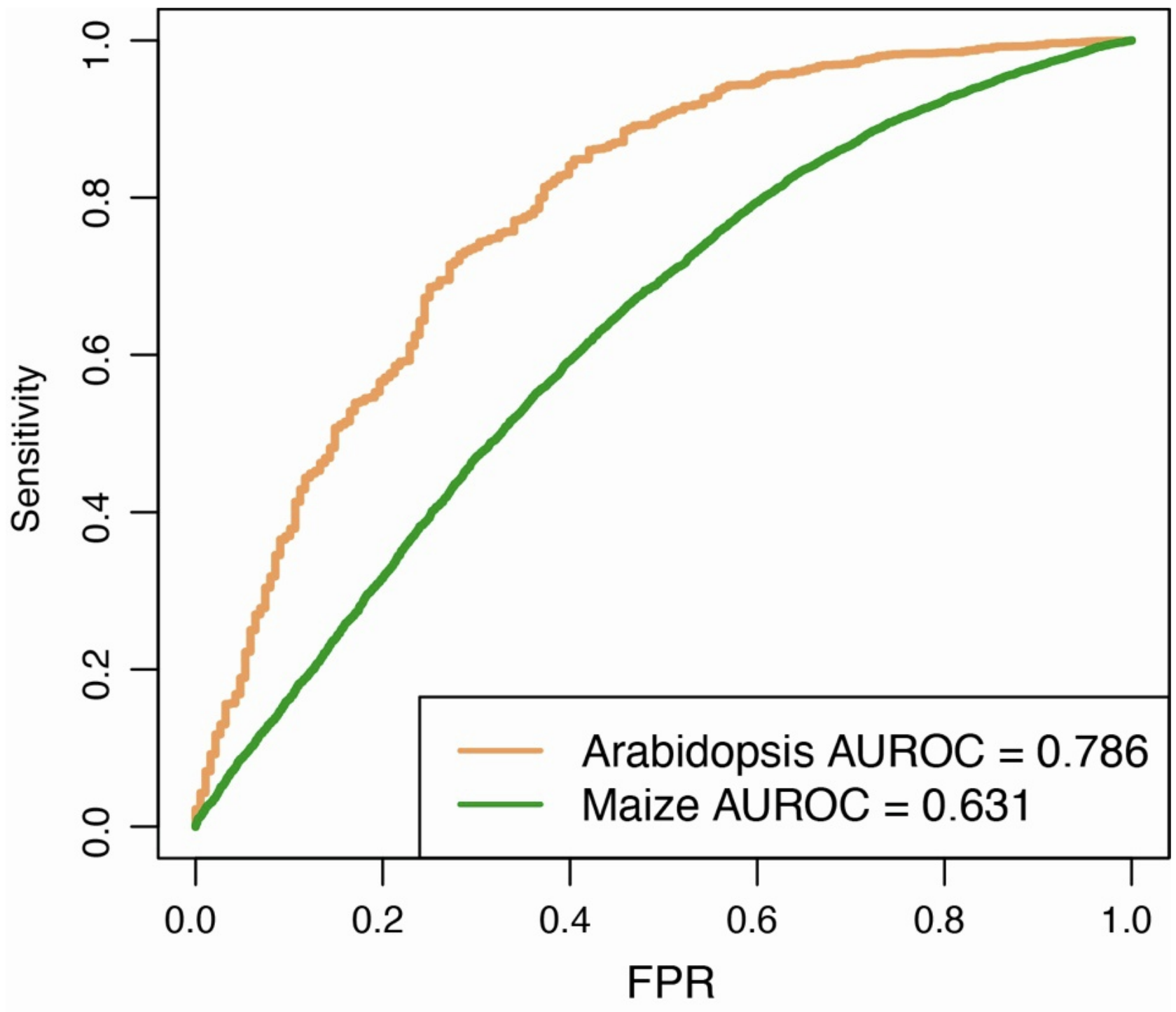


**Figure S15. Cross-species transfer performance for gene expression prediction, related to Figure 5.** ROC curves showing performance when training on Arabidopsis and testing cross-species transfer to maize.

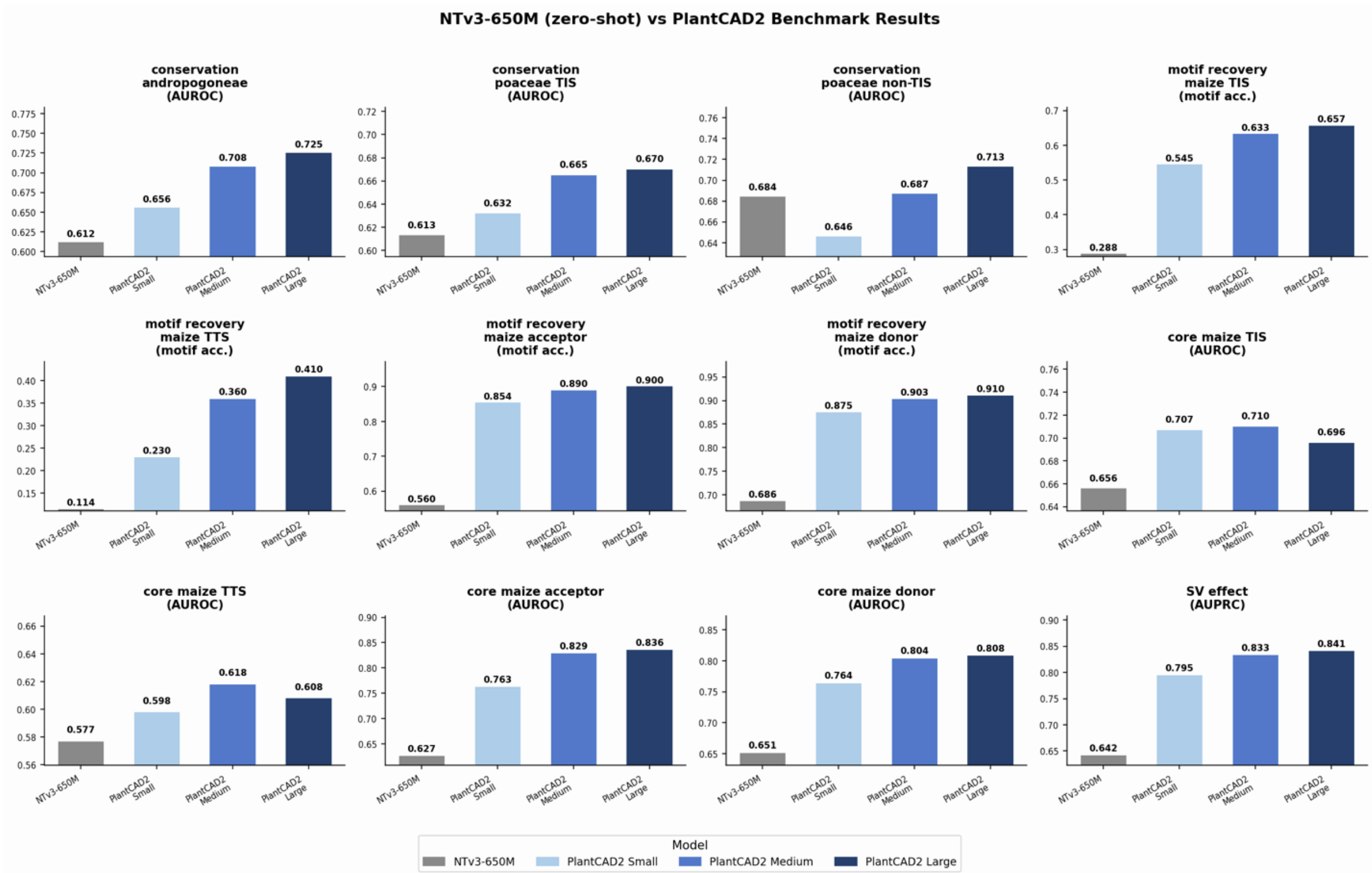


**Figure S16. Zero-shot performance comparison between PlantCAD2 and NTv3, related to Table 1.**

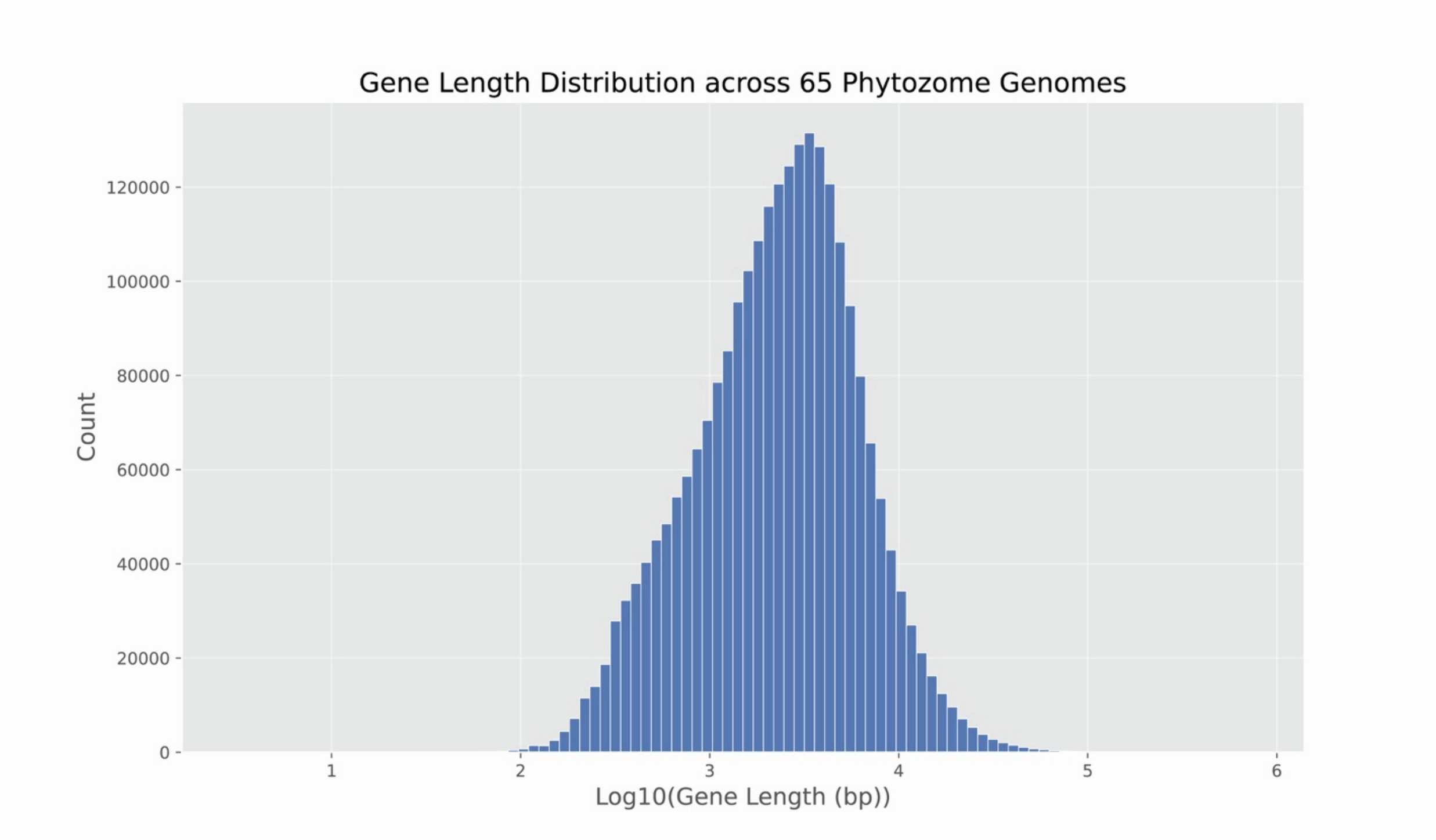


**Figure S17. Gene length distribution of 65 pretraining genomes, related to STAR Methods.**
