# Supplementary material for: PlantCAD2: A DNA foundation model for interpreting genomes across flowering plants
Source: Cell Genom. 2026 Aug 7;6(9):101329. doi: 10.1016/j.xgen.2026.101329 (PMC13576720; doi:10.1016/j.xgen.2026.101329)
Supplement: Supplementary file 2 [file mmc14.pdf]

Cell Genomics

Article

PlantCAD2: A DNA foundation model for interpreting genomes across flowering plants

Graphical abstract

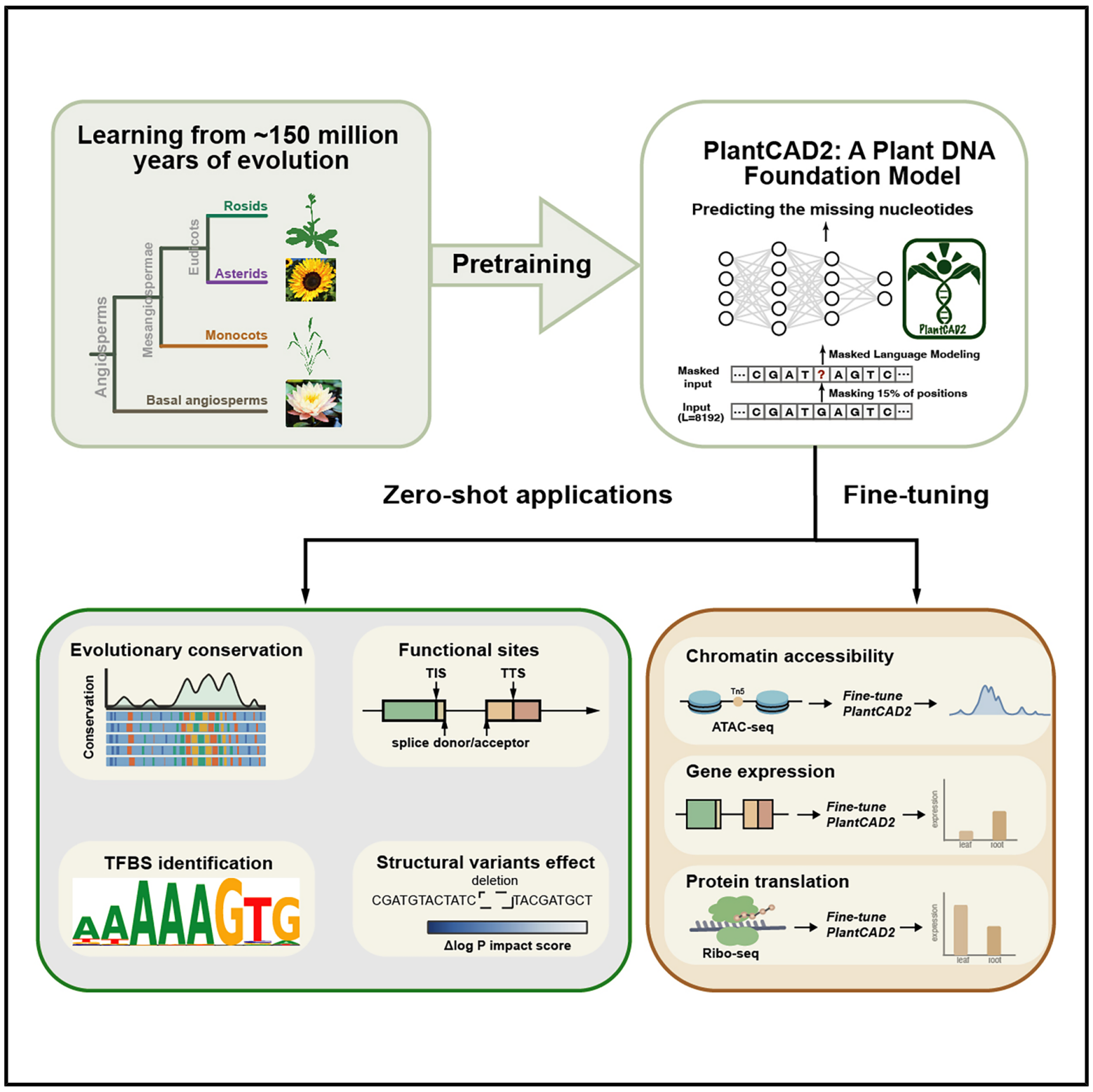

Highlights

- PlantCAD2 is a DNA foundation model pre-trained on 65 angiosperm genomes
- PlantCAD2 achieves state-of-the-art performance to capture evolutionary conservation
- Fine-tuning enables cross-species chromatin, expression, and translation prediction
- PlantCAD2 generalizes to plant lineages absent from pre-training

Authors

Jingjing Zhai, Aaron Gokaslan, Sheng-Kai Hsu, ..., Matt Pennell, Volodymyr Kuleshov, Edward S. Buckler

Correspondence

jz963@cornell.edu (J.Z.),
mpennell@cornell.edu (M.P.),
vk379@cornell.edu (V.K.),
ed.buckler@usda.gov (E.S.B.)

In brief

Zhai et al. introduce PlantCAD2, a DNA foundation model pre-trained on 65 angiosperm genomes. Despite being 10-fold smaller, PlantCAD2 outperforms the 7-billion-parameter Evo2 on most plant-specific zero-shot tasks, demonstrating that phylogenetically focused pre-training captures regulatory grammar more effectively. Through fine-tuning, it predicts chromatin accessibility, gene expression, and translation across species.

Zhai et al., 2026, Cell Genomics *6*, 101329
September 9, 2026 © 2026 The Authors. Published by Elsevier Inc.
https://doi.org/10.1016/j.xgen.2026.101329

CellPress

Cell Genomics

CellPress
OPEN ACCESS

Article

PlantCAD2: A DNA foundation model for interpreting genomes across flowering plants

Jingjing Zhai,[1,7,8,*] Aaron Gokaslan,[2,7] Sheng-Kai Hsu,[1] Szu-Ping Chen,[3] Zong-Yan Liu,[3] Edgar Marroquin,[2] Eric Czech,[5] Betsy Cannon,[5] Ana Berthel,[1] M. Cinta Romay,[1,3] Matt Pennell,[4,*] Volodymyr Kuleshov,[2,*] and Edward S. Buckler[1,3,6,*]
[1]Institute for Genomic Diversity, Cornell University, Ithaca, NY 14853, USA
[2]Department of Computer Science, Cornell University, Ithaca, NY 14853, USA
[3]Section of Plant Breeding and Genetics, Cornell University, Ithaca, NY 14853, USA
[4]Department of Computational Biology, Cornell University, Ithaca, NY 14853, USA
[5]Open Athena AI Foundation, New York, NY 10001, USA
[6]USDA-ARS, Ithaca, NY 14853, USA
[7]These authors contributed equally
[8]Lead contact
*Correspondence: jz963@cornell.edu (J.Z.), mpennell@cornell.edu (M.P.), vk379@cornell.edu (V.K.), ed.buckler@usda.gov (E.S.B.)
https://doi.org/10.1016/j.xgen.2026.101329

SUMMARY

Flowering plants (angiosperms) exhibit extraordinary species diversity, ∼200-fold variation in genome size, and relatively compact coding regions, presenting both a unique challenge and opportunity for DNA language models. Here, we introduce PlantCAD2, an extended-context, plant-specific DNA language model with single-nucleotide resolution, pre-trained on 65 angiosperm genomes, together with a series of public benchmarks for evaluation. Comprehensive zero-shot testing shows that PlantCAD2 (676 million parameters) efficiently captures evolutionary conservation, surpassing the 7-billion-parameter Evo2 in 10 of 12 tasks. With parameter-efficient fine-tuning, PlantCAD2 outperforms the 1-billion-parameter AgroNT across seven cross-species tasks including chromatin accessible region, gene expression, and protein translation. Its 8,192-bp context window substantially improves accessible chromatin prediction in large genomes such as maize (area under the precision-recall curve [AUPRC] increasing from 0.587 to 0.711), underscoring the importance of long-range context for modeling distal regulation. These results establish PlantCAD2 as a powerful and versatile foundation model for plant genome annotation and interpretation across diverse species.

INTRODUCTION

Deciphering how DNA sequence encodes molecular functions, phenotypes, and fitness remains a fundamental goal in biology. The rapid decline in sequencing costs has enabled large-scale initiatives such as the Darwin Tree of Life project,[1] the Earth BioGenome Project,[2] the Vertebrate Genomes Project,[3] and the 10KP Plant Genome Project,[4] which collectively aim to sequence tens of thousands of species across the tree of life, with plants alone contributing over 1,000 assembled genomes.[5] While genomic data accumulates exponentially, functional annotations lag far behind, particularly in plants where labeled data exist for only a few model species and crops.[6] Flowering plants (angiosperms),[7] the dominant terrestrial clade,[8] underpin global food security and ecosystem function. They exhibit maximal biochemical complexity, extraordinary species diversity (over 300,000 species), wide genome size variation, and relatively compact coding regions compared with other eukaryotes—features that present both a unique challenge and an opportunity for computational approaches. This widening gap between available sequences and functional knowledge highlights the critical need for models that can learn from raw sequences alone and transfer knowledge across plant species.

Foundation models,[9] large neural networks pre-trained on vast collections of unlabeled data using self-supervised objectives, have emerged as a powerful approach to this challenge. Rather than requiring large, labeled datasets for each task, these models learn general representations that can be adapted to specific applications with minimal supervision. This paradigm has achieved significant success in protein science, where models such as ESM,[10–12] ProtTrans,[13] and ProBERT[14] have demonstrated strong performance in predicting protein function,[15] structure,[16] and variant effect.[17]

In contrast, genomic language models (LMs) are still rapidly evolving, with recent developments spanning DNA,[18–27] RNA,[28–30] and transcriptomes.[31–33] Early work such as the DNABERT[20] pre-trained BERT[34] model on the human genome showed improved regulatory element prediction. Multi-species pre-training soon proved critical for learning evolutionary conservation,[21,22,25,35] a principle first demonstrated in plants by GPN,[22] which was pre-trained on eight Brassicales genomes and achieved strong variant effect prediction. Broader multi-species models followed, including Nucleotide Transformer[25]

This is an open access article under the CC BY license (http://creativecommons.org/licenses/by/4.0/).

Table 1. Zero-shot evaluation summary compared with the best-performing benchmark models

Category	Task	Description	Metric	PlantCAD2 vs. best benchmark
Cross-species evolutionary conservation (Figure 2)	conservation within Andropogoneae (Genome-wide)	predict conserved vs. non-conserved sites using alignments within 35 Andropogoneae genomes (*n* = 19,030 vs. 19,030)	AUROC	**0.725** vs. 0.691
	conservation within Poaceae (non-TIS)	predict conserved vs. non-conserved coding sites (excluding TIS) within Poaceae (*n* = 103,368 vs. 80,317)		0.713 vs. **0.822**
	conservation within Poaceae (TIS)	predict conserved vs. non-conserved TIS sites (*n* = 26,650 vs. 10,012)		**0.670** vs. 0.551
Key junction recovery (Figure 3)	translation initiation site (maize)	recover masked ATG start codon (*n* = 39,035)	accuracy	**0.657** vs. 0.447
	translation termination site (maize)	recover masked TAG/TAA/TGA stop codon (*n* = 39,035)		**0.410** vs. 0.256
	splice donor (maize)	recover masked GT motif (*n* = 153,869)		**0.910** vs. 0.741
	splice acceptor (maize)	recover masked AG motif (*n* = 153,869)		**0.900** vs. 0.738
Within-species conservation (Figure S4)	translation initiation site (maize)	predict core TIS vs. non-core TIS in maize (*n* = 28,291 vs. 8,118)	AUROC	**0.710** vs. 0.624
	translation termination site (maize)	predict core TTS vs. non-core TTS in maize (*n* = 28,291 vs. 8,118)		0.618 vs. **0.628**
	splice donor (maize)	predict core splice donor vs. non-core splice donor in maize (*n* = 123,183 vs. 21,367)		**0.808** vs. 0.754
	splice acceptor (maize)	predict core splice acceptor vs. non-core acceptor in maize (*n* = 123,183 vs. 21,367)		**0.836** vs. 0.761
Structural variant effect (Figure S5)	structural variant effect prediction	predict conserved deletions vs. non-conserved deletions (*n* = 7,662 vs. 10,413)	AUPRC	**0.841** vs. 0.771

For key junction recovery and within-species conservation, comparable results were observed in tomato.
For each task, the bold and underscored value indicates the highest score.

(850 genomes excluding plants) and Evo[18] (prokaryotic and phage genomes). Within plants, AgroNT[24] extended pre-training to 48 genomes with longer context windows but sacrificed single-nucleotide resolution through non-overlapping k-mer tokenization. We previously developed PlantCaduceus[27] (PlantCAD), a DNA LM pre-trained on 16 divergent angiosperm genomes. It uses the Caduceus[26] architecture, a Mamba-based[36] design that efficiently models both DNA strands simultaneously. PlantCAD achieved up to a 7-fold improvement over the next-best model in cross-species gene annotation tasks and variant effect prediction tasks. However, its context window of 512 bp restricts its ability to model biological processes that depend on distal regulatory interactions.[37] Many regulatory elements influence gene expression over tens to hundreds of kilobases and are key contributors to phenotypic variation[38–40] yet remain challenging to capture with short-context models.

Most recently, Evo2[19] has scaled to 40 billion parameters trained across genomes spanning the entire tree of life. While such generalist models represent impressive engineering achievements, deploying a 40-billion-parameter model for genome-wide annotation across more than 300,000 angiosperm species is prohibitively expensive, placing it beyond the reach of most plant genomics laboratories. More fundamentally, pre-training across all domains of life may dilute the lineage-specific regulatory signals critical for any single clade.

In this paper, we introduce PlantCAD2, an improved DNA LM tailored to angiosperm genomes. PlantCAD2 is pre-trained using a masked language modeling objective on 65 curated flowering plant genomes. PlantCAD2 is built on the efficient Mamba2 architecture,[41] which scales linearly with sequence length instead of quadratically as in transformers.[42] It supports 8,192-bp input windows and reverse-complement equivariance, allowing the model to capture extended-context, strand-invariant regulatory features. To reduce pre-training bias, we applied sampling strategies that both down-weight repetitive sequences and emphasize coding and regulatory regions. Subsequently, we first evaluated PlantCAD2 on 12 comprehensive benchmarks using a zero-shot strategy, demonstrating its efficiency and capacity to capture evolutionary conservation (Table 1). We then fine-tuned the model on seven functional genomics tasks, including chromatin accessibility, gene expression, and protein abundance, to further demonstrate its state-of-the-art cross-species predictive ability (Table 2). Together, these results highlight PlantCAD2's ability to generalize across species and tasks and to serve as a versatile foundation model for plant genome interpretation.

RESULTS

PlantCAD2: An extended-context DNA LM for angiosperms

PlantCAD2 builds on the original PlantCAD[27] DNA LM, preserving its single-nucleotide tokenization and masked language modeling objective while introducing four major improvements: architectural efficiency, context length, parameter scale, and phylogenetic breadth (Figure 1A). First, PlantCAD2 retains the

Table 2. Fine-tuning evaluation summary compared with the best-performing benchmark models

Category	Task	Dataset (train → test)	Task type	Metric	PlantCAD2 vs. best benchmark
Chromatin accessibility (Figure 4)	cross-species accessible regions	*Arabidopsis* → 10 species	binary classification	AUPRC (mean across 10)	**0.409** vs. 0.340
	cross-species accessible regions (multi-species)	9 species → 2 species	binary classification	AUPRC (mean across 2)	**0.570** vs. 0.499
	cell-type-specific accessible regions	maize (hold-out chr10)	multi-label classification	AUPRC (mean across 92 cell types)	**0.662** vs. 0.650
Gene expression (Figure 5)	cross-species leaf gene on/off prediction	15 Andropogoneae → 26 NAM genomes	binary classification	AUROC	**0.854** vs. 0.819
	cross-species leaf absolute gene expression	15 Andropogoneae → 26 NAM genomes	regression	Spearman correlation	**0.633** vs. 0.616
Protein Translation (Figure 5)	cross-species leaf translation on/off prediction	*Arabidopsis* → maize	binary classification	AUROC	**0.692** vs. 0.597
	cross-species leaf absolute translation abundance	*Arabidopsis* → maize	regression	Spearman correlation	**0.321** vs. 0.181

Sample sizes (n) for training and test sets are provided in supplemental tables.
For each task, the bold and underscored value indicates the highest score.

Caduceus[26] architecture with its bidirectional, reverse-complement-equivariant design, but it replaces the original Mamba[36] blocks with Mamba2 blocks.[41] Mamba2 has substantial advantages over Mamba1, leveraging structured state space duality for more efficient parallel training and simplifying recurrence computations to reduce memory usage (see STAR Methods). Compared to traditional transformer architectures,[42,43] PlantCAD2 model architecture shows a much slower increase in inference time than modernBERT models under the same input and output dimensions (Figure S1A) due to the inherent efficiency of state space models in handling long sequences.[36,44] Exploiting this efficiency, PlantCAD2 takes 8,192-bp windows, which is a 16-fold increase over the 512-bp windows used in PlantCAD. Second, to evaluate the effect of model sizes on performance, we trained a series of depth-scaled PlantCAD2 models of 88 million, 311 million, and 694 million parameters (Figures 1A and 1B), which we named PlantCAD2-S, PlantCAD2-M, and PlantCAD2-L respectively. As expected, following pre-training, the largest model (PlantCAD2-L) demonstrated the best masked token prediction accuracy (0.657), followed by PlantCAD2-M (0.641) and PlantCAD2-S (0.598), when evaluated on hold-out test set by randomly masking 15% of nucleotides per sequence (Figure S1B). However, the largest model also shows slowest inference speed at ~32,238 nucleotides per second on an NVIDIA H100 80 GB PCIe GPU (Figure 1C) and uses ~27 GB of GPU memory with a batch size of 32 (Table S1), reflecting the typical trade-off between accuracy and computational efficiency. The smallest PlantCAD2 model, PlantCAD2-S, achieves a 4.5-fold (Figure 1C) higher inference throughput while requiring considerably less GPU memory at equivalent batch sizes (Table S1), highlighting its practicality for high-throughput analyses. Despite differences in model size, the three models showed high correlation in their per-species prediction accuracies ($r > 0.97$), suggesting consistent learning patterns across scales (Figure S1C; Table S2). Third, to assess the effect of input length on pre-training accuracy, we varied the context window size from 512 to 8,192 bp and evaluated performance by masking the central token. All three models showed improved masked token prediction accuracy with longer contexts, underscoring the importance of extended context for modeling kilobase-scale genomic dependencies (Figure 1D). Lastly, we expanded the evolutionary diversity of the training dataset from 16 to 65 angiosperm genomes (Figure 1E; Table S2), selecting one representative species per genus to maximize phylogenetic breadth. When analyzing pre-training performance across species, we found a weak positive correlation between genome size and masked token accuracy ($r = 0.525$; Figure S1D; Table S2). This relationship is likely driven by the fact that larger genomes tend to contain more repetitive sequences.[45] Since the masked language modeling objective can predict repetitive elements more easily than non-repetitive elements even after applying down-sampling and down-weighting (see STAR Methods), the amount of repeats in the test set could inflate accuracy.[22,27,35] Consistent with our expectation, we also detected a positive correlation between the number of repeats in the test set and masked language modeling accuracy (Figure S1E); this also highlights the importance of down-sampling and down-weighting repetitive sequences[35] in pre-training DNA LMs.

PlantCAD2 accurately predicts evolutionary conservation with a zero-shot strategy

Evolutionary conservation, commonly estimated from multiple sequence alignment (MSA), is widely used to identify deleterious mutations that may reduce organismal fitness.[46–49] However, estimating a genome-wide MSA is particularly challenging in plants due to extensive transposable element (TE) insertions and their high turnover rate, which obscure orthologous relationships outside of conserved coding regions.[50] This limitation highlights the need for alignment-free approaches to assess conservation across diverse plant genomes. Given that PlantCAD2 is pre-trained on 65 evolutionary distant species,

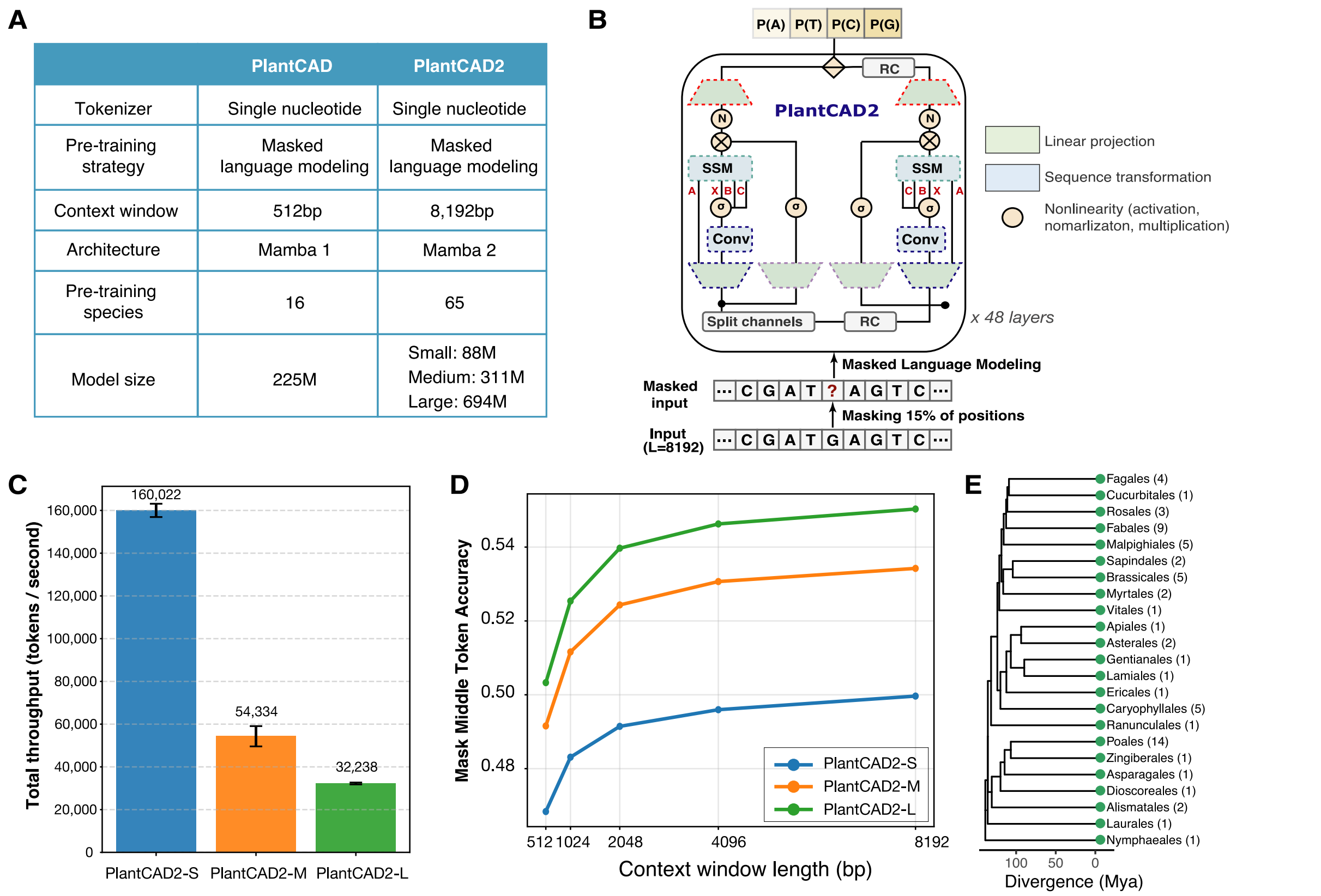

Figure 1. Overview of the PlantCAD2 model

(A) Comparison of PlantCAD1 and PlantCAD2 model configurations. PlantCAD2 introduces a longer context window, upgraded architecture (Mamba2), expanded pre-training species set, and scaled model sizes (small, 88M; medium, 311M; large, 694M parameters) while maintaining single-nucleotide tokenization.

(B) Schematic of the PlantCAD2 architecture based on Mamba2 with reverse-complement (RC) equivariance, convolutional and state space modules (SSM), and a masked language modeling objective applied to 8,192-bp input sequences.

(C) Total throughput (tokens/s) of PlantCAD2 models on NVIDIA H100 80 GB PCIe GPU across batch sizes (1–64). Values on the bar represent mean throughput across batch sizes; error bars represent the standard deviation across batch sizes (1–64).

(D) Effect of context window length on model performance. The *y* axis shows the prediction accuracy of three models when masking the single central token in the held-out test set.

(E) Phylogenetic distribution of the 65 angiosperm genomes across flowering plant orders. Numbers in parentheses indicate the number of species included from each order.

we hypothesize that PlantCAD2 can be used to predict evolutionary conservation without an MSA. We first evaluated how accurate PlantCAD2 is to distinguish highly conserved sites versus less conserved sites using a zero-shot strategy. As illustrated in Figure 2A, we used the masked nucleotide/token prediction accuracy from the frozen model to represent per-base conservation, which means highly conserved bases would receive higher predicted probabilities for the reference allele, whereas less conserved bases would yield lower confidence scores. We benchmarked the performance of PlantCAD2 against three baselines: its predecessor PlantCAD,[27] GPN[22] (a plant-specific DNA LM trained on Brassicales genomes), and Evo2,[19] a general-purpose DNA LM pre-trained using a causal language modeling (CLM) objective, also known as next-token prediction. Unlike masked language modeling, which enables access to both upstream and downstream context, CLM imposes a strict left-to-right constraint, meaning Evo2 is inherently unidirectional. Therefore, we input the entire sequences without masking for Evo2 and use the likelihood of the model to represent conservation. Notably, Evo2 was trained at a substantially greater scale, with 7 billion parameters and 9.3 trillion nucleotides, which is over 310 times more training data than used for PlantCAD2, therefore providing a rigorous benchmark for assessing the efficiency and representational power of our models. We excluded AgroNT from zero-shot evaluation as its non-overlapping k-mer tokenization strategy prevents single-nucleotide resolution tasks, and we previously demonstrated its limited zero-shot capabilities.[27]

We assessed this strategy in three independent tasks. First, we performed cross-species alignments of 34 Andropogoneae genomes[27] to the sorghum reference genome and identified highly conserved and less conserved sites based on alignment coverage and identity (see STAR Methods). PlantCAD2 consistently outperformed PlantCAD in distinguishing highly conserved

CellPress
OPEN ACCESS

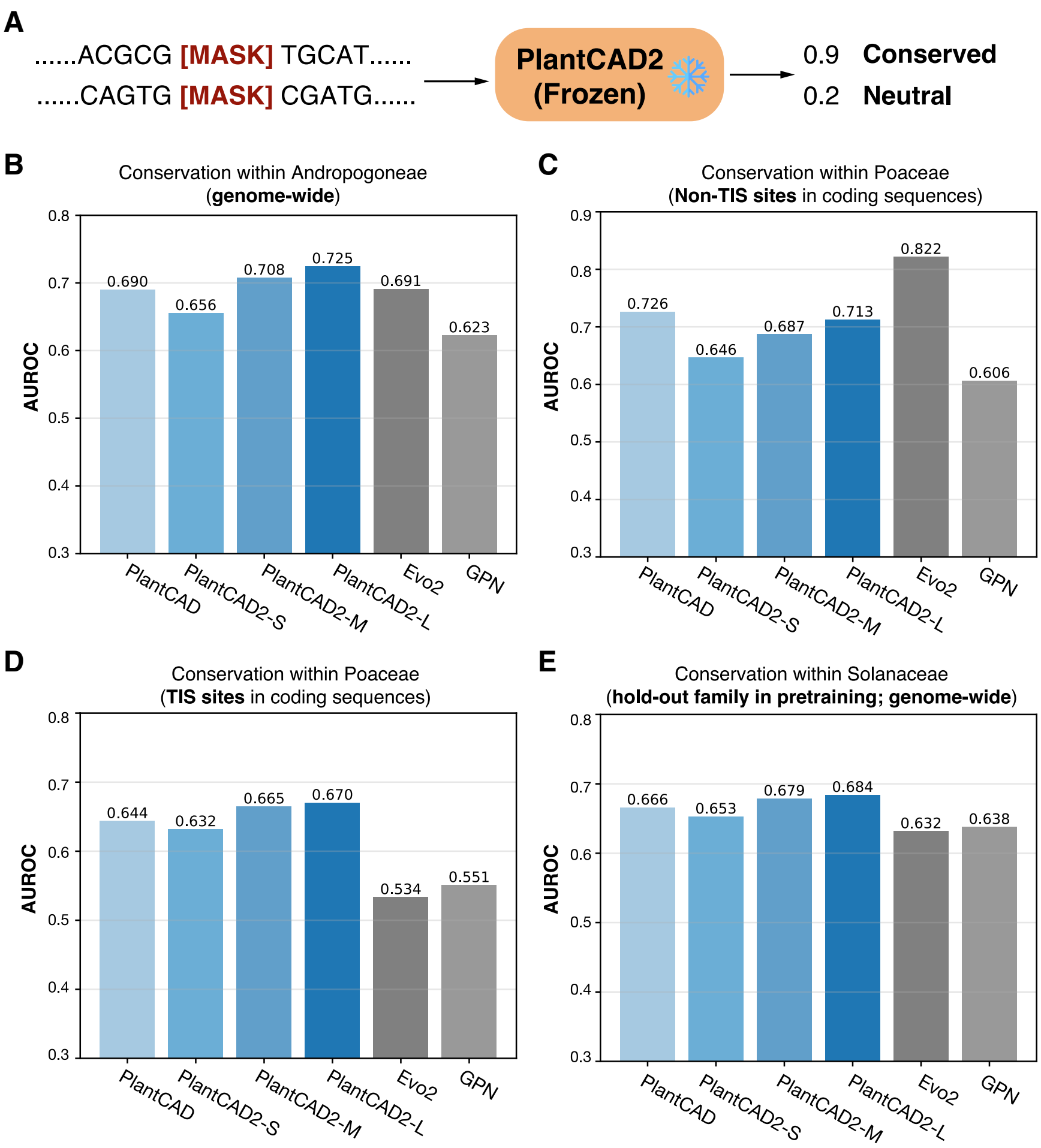

Figure 2. PlantCAD2 accurately predicts evolutionary conservation using zero-shot strategy

(A) Illustration of the zero-shot conservation approach. For each masked token (nucleotide) in the reference sequence, the frozen PlantCAD2 model outputs the probability of the reference allele; higher probabilities indicate higher evolutionary constraint.

(B) AUROC for predicting conserved versus neutral sites in sorghum, representing conservation within the Andropogoneae tribe.

(C and D) AUROC for predicting conservation within Poaceae using sites in coding regions, evaluated separately for non-TIS (C) and TIS (D).

(E) AUROC for conservation prediction within Solanaceae using SNPs from potato. Solanaceae species were entirely excluded from the PlantCAD2 pre-training, making this a hold-out family used to evaluate cross-lineage generalization.

from less conserved sites in the sorghum genome, with the largest PlantCAD2 achieving the highest area under the receiver operating characteristic curve (AUROC) (Figure 2B; Table S3). Notably, PlantCAD2-M achieved slightly better performance than Evo2 (AUROC 0.708 vs. 0.691) despite being ∼22-fold smaller (311 million vs. 7 billion parameters), while PlantCAD2-L, being ∼11-fold smaller (694 million parameters), further improved to 0.73. This demonstrates that our PlantCAD2 models can match or exceed Evo2's performance with far fewer parameters. Given that PlantCAD2 is pre-trained with a context window of 8192 bp, we also examined the effect of context length on conservation prediction. AUROC scores increased with longer sequence contexts, plateauing at 4,096 bp for all PlantCAD2 models (Figure S2A; Table S3). These findings indicate that evolutionary constraint signals benefit from broader sequence context and that larger models with extended receptive fields are better suited to capture these dependencies.

In the second task, we used MSAs from coding sequences of 325 Poaceae genomes to calculate phyloP scores and define highly conserved sites (phyloP > 5) and less conserved sites (phyloP < 1.5). While the relationship between selection and phyloP scores can be nuanced,[51] restricting phyloP calculation to coding regions helps mitigate alignment noise caused by the very high TE turnover rate in plant genomes,[52] providing a more reliable benchmark for conservation prediction. Given Evo2's unidirectional nature, we hypothesized it might struggle with features requiring bidirectional context, particularly translation initiation sites (TISs), where both upstream regulatory motifs in the 5′ UTR and downstream coding sequence context critically influence start codon recognition and conservation.[53] To test this hypothesis, we separately evaluated performance on TIS versus non-TIS positions within coding sequences. For non-TIS sites, PlantCAD2 models showed lower performance compared to Evo2 (Figure 2C; Table S3), potentially because Evo2's training data included mature mRNA sequences while PlantCAD2 was trained exclusively on genomic DNA, giving Evo2 an advantage in coding sequence conservation tasks. We also note that PlantCAD slightly outperforms PlantCAD2 on this task (Figure 2C), likely because PlantCAD's 512-bp pre-training windows closely match the coding sequence (CDS)-centered inputs used here, whereas PlantCAD2's 8-kb genomic windows include intronic and intergenic contexts absent from these restricted inputs. Despite this mismatch, PlantCAD2 remains competitive. Interestingly, when evaluating TIS conservation, we observed a strong bias in Evo2: its AUROC dropped drastically to 0.534, barely above random. In contrast, PlantCAD2 maintained robust performance (AUROC, 0.632–0.670; Figure 2D; Table S3). Even the 65-million-parameter GPN outperformed the 7-billion-parameter Evo2 on this task (AUROC of 0.551), further highlighting Evo2's architectural limitations for TIS prediction (Figure 2D; Table S3). This TIS-specific weakness in Evo2 validates our hypothesis: without access to coding sequence contexts that are more evolutionary constrained, Evo2 cannot properly assess the conservation patterns at translation start sites. Consistent with the previous task,

we observed only modest improvements from longer context windows, indicating that evolutionary conservation signals are predominantly local (Figures S2B and S2C). These results demonstrate that PlantCAD2 provides more consistent and unbiased conservation predictions across different genomic contexts.

To further assess whether PlantCAD2 generalizes to lineages absent from pre-training, we evaluated its zero-shot conservation predictions in Solanaceae, a major angiosperm family not represented in the 65 pre-training species. This evaluation used SNPs from the potato (*Solanum tuberosum* L.) genome, with Genomic Evolutionary Rate Profiling (GERP) scores[54] calculated from an MSA of 95 Solanaceae genomes.[55] Conserved sites were defined as SNPs with GERP scores greater than 1.5 and neutral sites as SNPs with GERP scores lower than 0. As shown in Figure 2E, PlantCAD2 models achieved higher AUROC than both Evo2 and GPN, despite multiple *Solanum* genomes being part of the Evo2 pre-training corpus but absent from PlantCAD2 pre-training. Additionally, to understand functional constraints across the entire genome, we stratified the evaluation into six distinct genomic contexts: CDS, upstream (promoters), downstream, introns, intergenic regions, and TEs (Figure S3). As expected, accuracy was higher in coding versus non-coding regions (Figure S3), likely because the stronger functional constraints on CDS make these patterns easier for the model to capture. When comparing models across the five non-coding categories, PlantCAD2 consistently showed the best performance in four categories (upstream, downstream, intergenic, and TEs) with GPN showing only a marginal advantage in introns. Notably, PlantCAD2 consistently surpassed Evo2 across all six genomic contexts. The strong consistent performance across three phylogenetically distant angiosperm lineages indicates that PlantCAD2 captures fundamental conservation principles shared across flowering plants rather than lineage-specific patterns. This enables accurate alignment-free conservation inference even in lineages unseen during pre-training.

PlantCAD2 accurately predicts within-species conserved transcriptional and translational junction sites with zero-shot strategy

We next quantified how well PlantCAD2 captured the sequence context that defines key transcriptional and translational junctions. Using a similar zero-shot strategy, we designed four tasks to recapitulate motifs (Figure 3). Instead of masking one base pair, for each annotated junction, we replaced the annotated motif with consecutive [MASK] tokens. Given that non-canonical motifs do exist in plants,[56] we retained all annotated sites without filtering for canonical signals. Consequently, the evaluation included both canonical and non-canonical variants as defined in the reference annotation. For example, TISs included both canonical ATG and non-canonical start codons such as GTG and TTG. We then extracted a fixed 8,192-bp window centered on the masked motif and presented the entire masked sequence to the model without fine-tuning. A prediction was considered correct if the model's top-1 reconstruction exactly matched the ground truth motif found in the genome.

As above, we benchmarked the performance of PlantCAD2 against PlantCAD, GPN, and Evo2. Given what we observed in Figure 2D, that Evo2 is limited with its poor TIS conservation prediction, we evaluated it using two configurations: (1) forward sequences (Evo2-fwd), where the model uses upstream context to predict the junction, and (2) reverse-complement sequences (Evo2-rc), where the model uses downstream context (reverse complemented) to predict the junction. For GPN and PlantCAD, which are both limited to context windows of 512 bp, we used 512-bp windows centered on the junctions for evaluation.

When evaluated on both maize (*Zea mays*), which was included in pre-training and tomato (*Solanum lycopersicum*), which was not included in pre-training, PlantCAD2 consistently outperformed PlantCAD1 across all junction types (Figure 3; Table S4). Notably, even the smallest PlantCAD2 model (88 million parameters) outperformed the original PlantCAD (311 million parameters), demonstrating that architectural improvements and expanded phylogenetic diversity (65 vs. 16 genomes) provide substantial benefits beyond parameter scaling alone. Accuracy increased with model size, following expected scaling law, with the largest model achieving the highest masked-motif prediction accuracy across both species.

As expected, Evo2 showed strong directional effects. For junctions where downstream context is more informative (TIS and splice acceptor), Evo2-rc performed better, as the reverse-complement orientation allows the model to "see" the downstream coding sequences that provide stronger signals. Conversely, for junctions where upstream context matters more (TTS and splice donor), Evo2-fwd showed superior performance. The sharp contrast in performance between forward sequences and reverse complemented sequences reflects a fundamental limitation of causal LMs: their unidirectional nature prevents them from accessing both upstream and downstream signals simultaneously. In contrast, PlantCAD2's bidirectional and reverse-complement equivariant design achieved robust performance regardless of sequence orientation, consistently leveraging both upstream and downstream context for all junction types.

While recovering canonical junction motifs demonstrates basic sequence understanding, we next tested whether PlantCAD2 captures deeper evolutionary signals that distinguish core genes (evolutionarily constrained and present across taxa) from non-core genes (rapidly evolving and taxa specific). We extracted each model's log likelihood of the canonical motif as a conservation score and evaluated binary classification performance using AUROC (Figure S4). For Evo2, we selected the optimal orientation based on junction type (forward for TTS/donor, reverse-complement for TIS/acceptor) as determined above. Remarkably, even though the tomato genome was excluded from PlantCAD2's 65 pre-training genomes, PlantCAD2 consistently outperformed Evo2—which did include tomato during pre-training. This demonstrates strong cross-species generalization: PlantCAD2 learned transferable conservation patterns from other angiosperms that effectively predict functional constraints in unseen species. These results highlight PlantCAD2's ability to capture fundamental

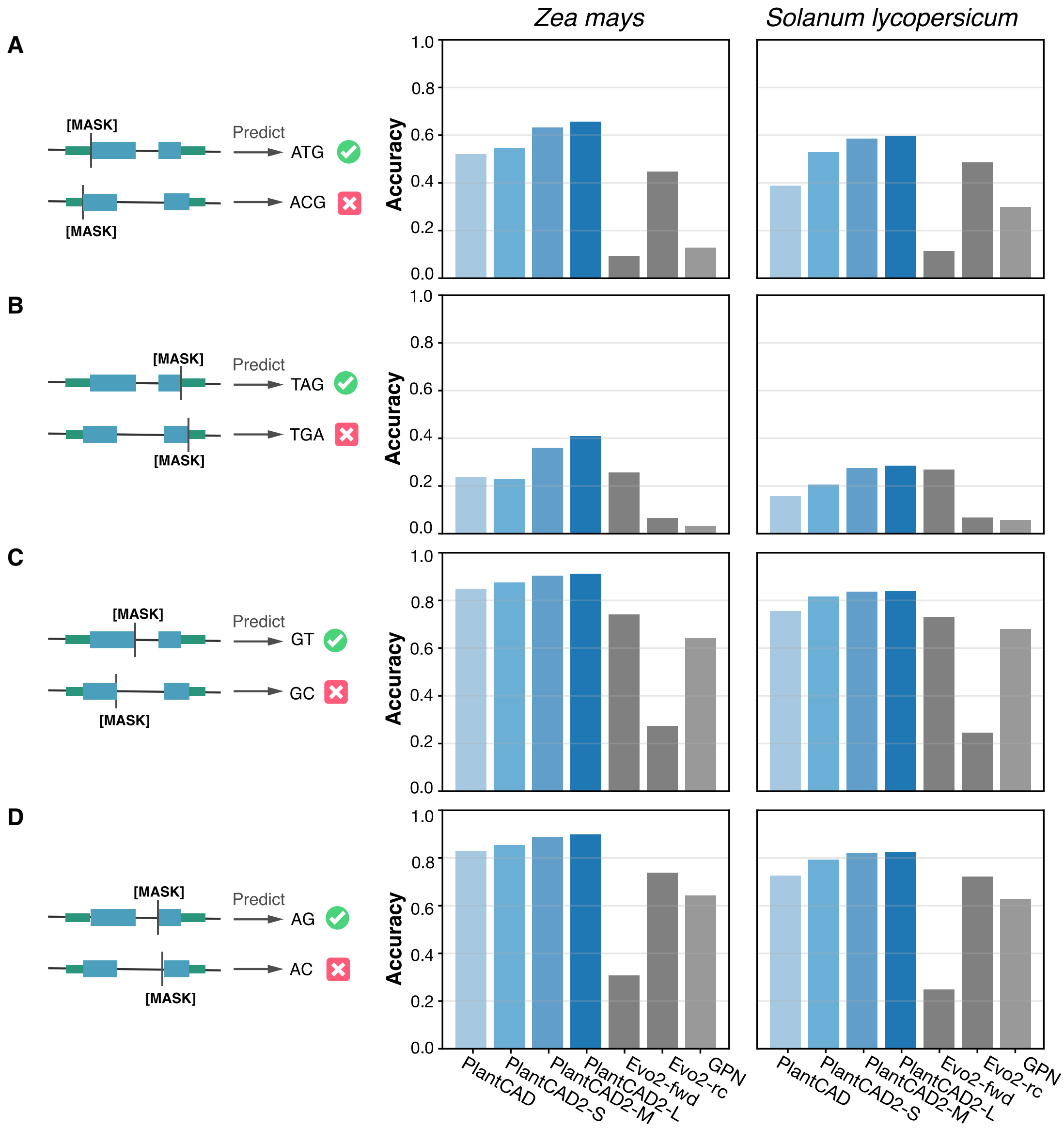

Figure 3. PlantCAD2 accurately predicts transcriptional and translational junction sites using zero-shot masked-motif prediction
Left panels show the masking strategy where canonical motifs are replaced with [MASK] tokens and models predict the correct sequence. Right panels show prediction accuracy for each model on maize (left, included in training) and tomato (right, excluded from training).
(A) TISs (ATG masking).
(B) Translation termination sites (TAG/TGA/TAA masking).
(C) Splice donor sites (GT masking).
(D) Splice acceptor sites (AG masking).

evolutionary principles instead of just recognizing simple motif recognition.

PlantCAD2 predicts functional structural variants with zero-shot strategy

In addition to single-nucleotide conservation, we investigated whether PlantCAD2 can generalize to predicting the functional impact of structural variants (SVs), such as small deletions, using a zero-shot approach. To do this, we simulated a set of deletions in the *Arabidopsis* genome and computed the Δlog P score, defined as the log likelihood ratio between the mutated and reference sequences surrounding the deletion site, averaged across the deletion window (Figure S5A). To assess how well Δlog P reflects the functional deletions, we utilized two complementary conservation metrics derived from MSAs of 63 genomes[57]: phyloP,[58] which measures conservation or acceleration at individual sites, and phastCons,[58] which identifies conserved elements by analyzing patterns across adjacent sites. We classified deletions as either “highly conserved” or “less conserved” based on the average scores from each metric.

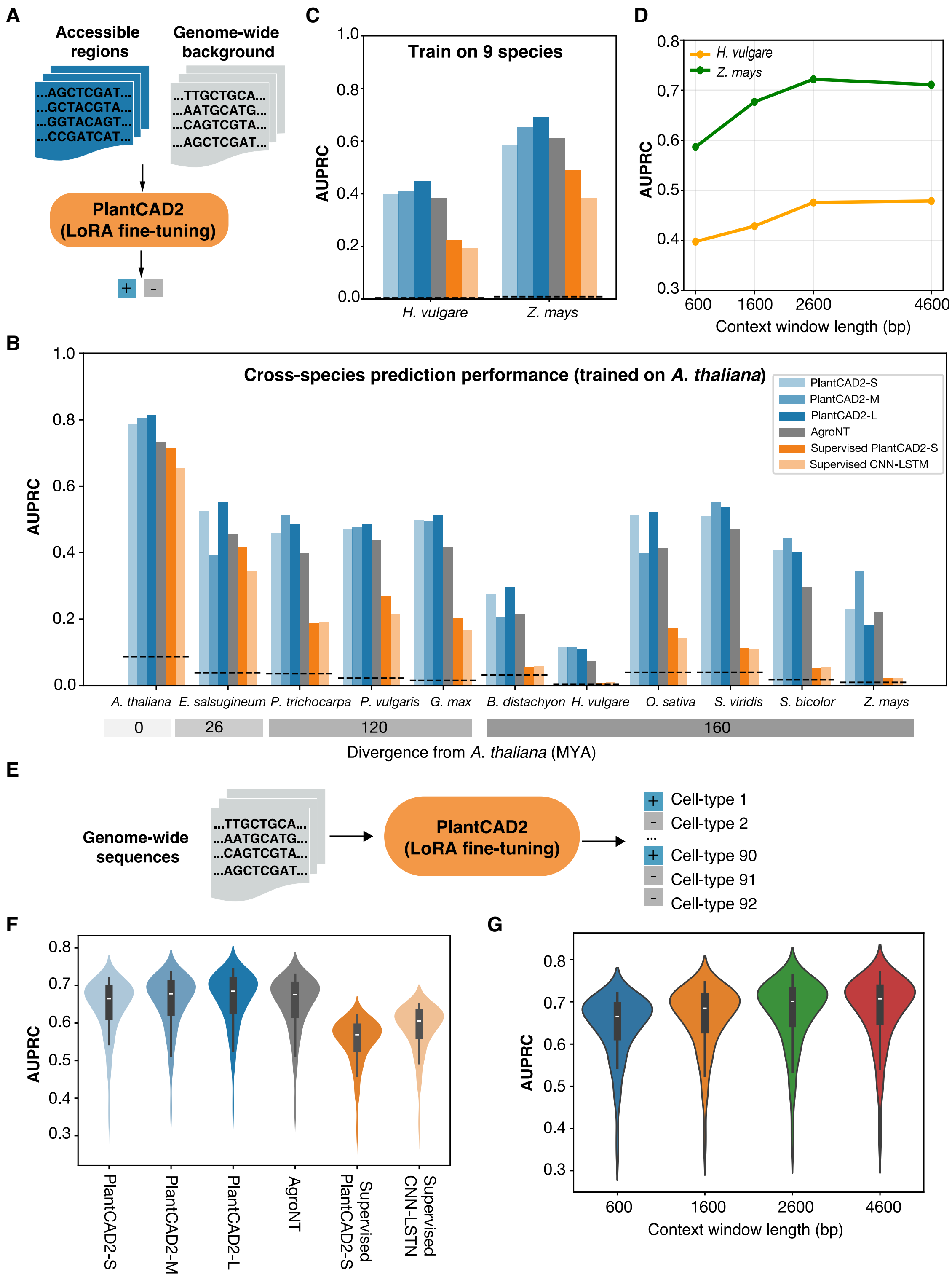

Figure 4. PlantCAD2 predicts chromatin accessibility across species and cell types

(A) LoRA fine-tuning approach for binary accessibility prediction using ATAC-seq peaks versus genomic background.

(B) Cross-species AUPRC performance when trained on *Arabidopsis*, showing superior generalization of PlantCAD2 models compared to supervised baselines across evolutionary distances.

(C) Multi-species training performance on held-out barley and maize.

(legend continued on next page)

Given more negative Δlog P represents higher conservation, we observed PlantCAD2's negative Δlog P scores showed strong positive correlation with both phyloP-based and phastCons constraint scores (Figures S5B and S6), with the model assigning higher likelihoods to mutations in evolutionarily constrained regions. To quantify this relationship, we binarized deletions into highly conserved and less conserved categories based on either phyloP (Figure S5A) or phastCons scores (Figure S6A) and evaluated whether Δlog P could discriminate between them. PlantCAD2 achieved robust classification performance either using phyloP (Figure S5C; Table S5) or phastCons (Figure S6), with even the 88-million-parameter PlantCAD2-S outperforming the 7-billion-parameter Evo2, highlighting the advantage of plant-specific training over general-purpose models again. Additionally, we also observed classification performance is saturated with just 20 bp of the flanking sequence on each side (Figure S7), indicating that local sequence context sufficiently captures the functional impact of small deletions. This strong performance is particularly impressive given that PlantCAD2 was never explicitly trained on structural variants, suggesting it learned generalizable sequence constraint patterns during pre-training. These results suggest that PlantCAD2's learned representations generalize beyond single-nucleotide changes, capturing broader sequence dependencies relevant to non-coding structural variation. This provides a scalable alternative to traditional alignment-based conservation methods. Notably, this also represents one of the first efforts to use DNA LMs for estimating indel effects in plant genomes, underscoring the potential of foundation models in addressing complex variant interpretation challenges.

Transcription factor binding sites revealed by high-confidence predictions of PlantCAD2

To evaluate the interpretability and regulatory grammar capture of PlantCAD2, we first assessed whether the model's high-confidence predictions align with experimental verified transcription factor binding sites (TFBSs) using a zero-shot approach.[22] We masked each position within DNA affinity purification sequencing (DAP-seq)-defined DREB2 peak regions[59] and generated importance scores from the model's output distribution, then clustered these into motifs using TF-MoDISco.[60] DREB2 (AP2/ERF family) was selected as a representative transcription factor because AP2/ERF TF family is prevalent and evolutionary conserved across angiosperms.[61]

The *de novo* motif discovered by TF-MoDISco with PlantCAD2 predictions showed a highly significant match to the canonical AP2/ERF profile (https://jaspar.elixir.no/matrix/MA1222.1) in the JASPAR database (Tomtom p = 0.0001; Figure S8). In contrast, while the Evo2 model also detected the motif, the match was substantially less significant (Tomtom p = 0.005), suggesting that PlantCAD2 captures plant-specific regulatory features with higher fidelity.

We then extended this strategy to performing *de novo* motif discovery across all 1.5-kb regions upstream of TISs in the *Arabidopsis* genome. This genome-wide analysis was designed to systematically identify representative *cis*-regulatory motifs captured by the pre-trained PlantCAD2 model beyond known DAP-seq peaks. In total, 104 high-confidence motifs were identified. Of the 104 high-confidence motifs, 27 matched known entries in the JASPAR plant database (Tomtom p < 0.05). This 26% match rate likely underestimates the true proportion of functional motifs, given that JASPAR's plant subset covers only a fraction of known plant transcription factor families and is heavily biased toward a few model organisms. Representative examples, highlighted in Figure S9, include BPC1 (AT2G01930), involved in ovule identity[62]; TSO1 (AT3G22780), a regulator of floral cell division[63]; SRS7/SPRI2 (AT1G19790), which modulates auxin-dependent reproductive barriers[64]; and ZML1 (AT3G21175), a GAGA-type factor mediating photoprotection.[65] These results may suggest these TFBS exhibit profound evolutionary conservation and indicate PlantCAD2 could be a useful tool to recover both established and potentially novel conserved regulatory elements. All discovered motifs and their statistics are available on GitHub: https://plantcad.github.io/TFBS.

Fine-tuning PlantCAD2 accurately predicts cross-species chromatin accessible regions and cell-type-specific accessible regions

We next investigated whether PlantCAD2 learned chromatin states by assessing its performance in predicting genome-wide chromatin accessibility across multiple plant species. We formulated this as a binary classification task, in which the model predicts whether a given genomic region corresponds to an accessible chromatin region, as defined by assay for transposase-accessible chromatin using sequencing (ATAC-seq) (STAR Methods). We used recently published ATAC-seq data including 11 diverse plant species, including both dicots and monocots.[66] In this task, positive examples correspond to accessible peaks from ATAC-seq, while negative examples were from genomic background regions. We used 600-bp genomic windows for all models, as this resolution captures the typical size of ATAC-seq peaks while providing sufficient sequence context for regulatory element prediction. Due to the biological reality of accessible regions comprising only a small fraction of the entire genome, this task is highly imbalanced (Table S6). For example, less than 1% of regions in the maize genome are labeled as positive.

To effectively leverage the pre-trained foundation model, we used a low-rank adaptation (LoRA)[67] fine-tuning strategy for PlantCAD2, which inserts small trainable rank-decomposition matrices into the feedforward layers while keeping the rest of the model frozen (Figure 4A). This approach updates only a small fraction of parameters, enabling efficient task-specific adaptation with minimal risk of overfitting or forgetting the pre-trained knowledge. To assess the contribution of pre-training, we

(D) Effect of context window length on accessibility prediction accuracy for PlantCAD2-S.
(E) Multi-label classification approach for cell-type-specific accessibility prediction.
(F) Performance comparison across models for 92 cell types.
(G) Context window effects on cell-type-specific prediction accuracy for PlantCAD2-S.

compared this approach to two supervised baselines: (1) a fully supervised version of PlantCAD2-S from scratch, where all model parameters were randomly initialized and updated during training; and (2) a commonly used CNN + LSTM[68] architecture trained from scratch. We also benchmarked against AgroNT,[24] another plant-specific DNA LM. We excluded GPN and PlantCAD due to their limited 512-bp context window. For other DNA LMs, we tailored our benchmarking strategy to the specific characteristics of each model. Specifically, we benchmarked against AgroNT using LoRA, as our previous study[27] demonstrated that AgroNT yields suboptimal performance with frozen embeddings but gains significant accuracy through fine-tuning, which is computationally feasible given its 1-billion-parameter size. In contrast, we evaluated the 7-billion-parameter Evo2 using frozen embeddings from the last hidden state, testing three input configurations (forward, reverse complement, and their average) to capture maximal information content. We probed these embeddings with both a linear classifier and XGBoost,[69] providing a comprehensive assessment of representation quality without computationally prohibitive fine-tuning. All models were trained using *Arabidopsis* and validated on hold-out chromosomes within *Arabidopsis* as well as on 10 additional test species spanning a broad phylogenetic range.

Given the strong class imbalance of this task, we measured model performance using the area under the precision-recall curve (AUPRC), which is more informative than AUROC in imbalanced classification settings. LoRA-fine-tuned PlantCAD2 consistently achieved the best performance in both within-species evaluation and cross-species generalization, outperforming supervised baselines and AgroNT across all test species (Figure 4B; Table S6). Furthermore, in the comparison using fixed (frozen) embeddings against the 7-billion-parameter Evo2, PlantCAD2 consistently achieved higher AUPRC scores across the 11 species, regardless of whether a linear or non-linear classifier was used (Figure S10). While the averaged embedding (combining forward- and reverse-complement strands) proved to be the most robust configuration for Evo2, it was still consistently outperformed by all PlantCAD2 models across all test species. Remarkably, even our smallest model, PlantCAD2-S, surpassed the 7-billion-parameter Evo2. On average, PlantCAD2-L yielded an AUPRC improvement of 0.1142 and 0.1735 over Evo2's best performance using Linear and XGBoost classifiers, respectively (Figure S10). This result highlights the efficiency of PlantCAD2 as an angiosperm DNA foundation model, demonstrating that domain-specific pre-training contributes more to representation quality than simply scaling up model parameters.

Across the diverse species tested, we observed a strong negative correlation between genome size and AUPRC, which reflects the increasing difficulty of distinguishing sparse regulatory elements in large intergenic regions (Figure S11). Despite this challenge, fine-tuned DNA LMs consistently outperformed supervised models trained from scratch (including CNN + LSTM and Supervised PlantCAD2-S), indicating that pre-training enables better learning of chromatin states, particularly when transferring knowledge across species (Figure 4B). Supervised models generalized well to closely related dicots but declined substantially for distant monocots, whereas fine-tuned PlantCAD2 retained strong accuracy across both, capturing regulatory features conserved across deep evolutionary divergence.

While the foundation model fine-tuned on *Arabidopsis* already demonstrated clear advantages in cross-species prediction, we further fine-tuned a multi-species version of PlantCAD2 using accessible chromatin regions from multiple plant genomes to enhance its robustness across diverse lineages. With maize and barley held out as test species, this multi-species model achieved impressive AUPRC scores of 0.691 for maize and 0.449 for barley (Figure 4C; Table S7). To investigate whether extended sequence context could further improve prediction accuracy, we maintained the same 600-bp labels but varied the input window size by including different amounts of flanking sequence. For computational efficiency, we conducted this analysis using PlantCAD2-S. Performance consistently improved with longer context windows, with AUPRC increasing from 0.587 to 0.711 for maize and from 0.398 to 0.479 for barley when extending from 600 to 4,600 bp (Figure 4D). This substantial improvement suggests that distal regulatory elements and broader chromatin context beyond the immediate peak boundaries contribute to accessibility prediction, highlighting the advantage of PlantCAD2's long-context architecture. These fine-tuned models serve as a robust predictor of chromatin accessibility across flowering plants and are publicly available to the community as a ready-to-use resource for regulatory annotation in non-model species.

To further assess whether PlantCAD2 can resolve cell-type-specific regulatory landscapes, we tested its ability to predict accessible chromatin regions identified through single-cell ATAC-seq (scATAC-seq) in maize.[70] In contrast to the binary classification task used for genome-wide accessibility, this task was framed as a multi-label classification problem, where each genomic window could be accessible in one or more cell types (Figure 4E). We curated high-confidence cell-type–specific peaks across major maize tissues from published scATAC-seq datasets (STAR Methods), using them as labels for multi-label fine-tuning and evaluation.

We applied the same LoRA fine-tuning strategy used in prior experiments, adapting PlantCAD2 to predict cell-type-specific accessibility using only a small number of trainable parameters. As in previous sections, we compared performance against two supervised baselines: a CNN + LSTM model trained from scratch and a fully supervised version of PlantCAD2. All models were trained on all maize cell types and evaluated on held-out chromosomes, with performance measured using micro-averaged precision-recall curves across cell types. Despite the complexity and subtlety of cell-type-specific regulatory signatures, LoRA fine-tuned PlantCAD2 can still achieve very high accuracy and outperformed other baselines (Figure 4F; Table S8). Similar to our genome-wide accessibility results, extending the input context window beyond the core 600-bp peak region further improved cell-type specificity, with AUPRC increasing from 0.665 to 0.707 when using 4,600-bp windows (Figure 4G). This suggests that cell-type-specific regulatory programs are influenced by broader chromatin context and distal regulatory interactions. The model captured both shared and lineage-specific accessibility patterns, demonstrating that pre-trained DNA

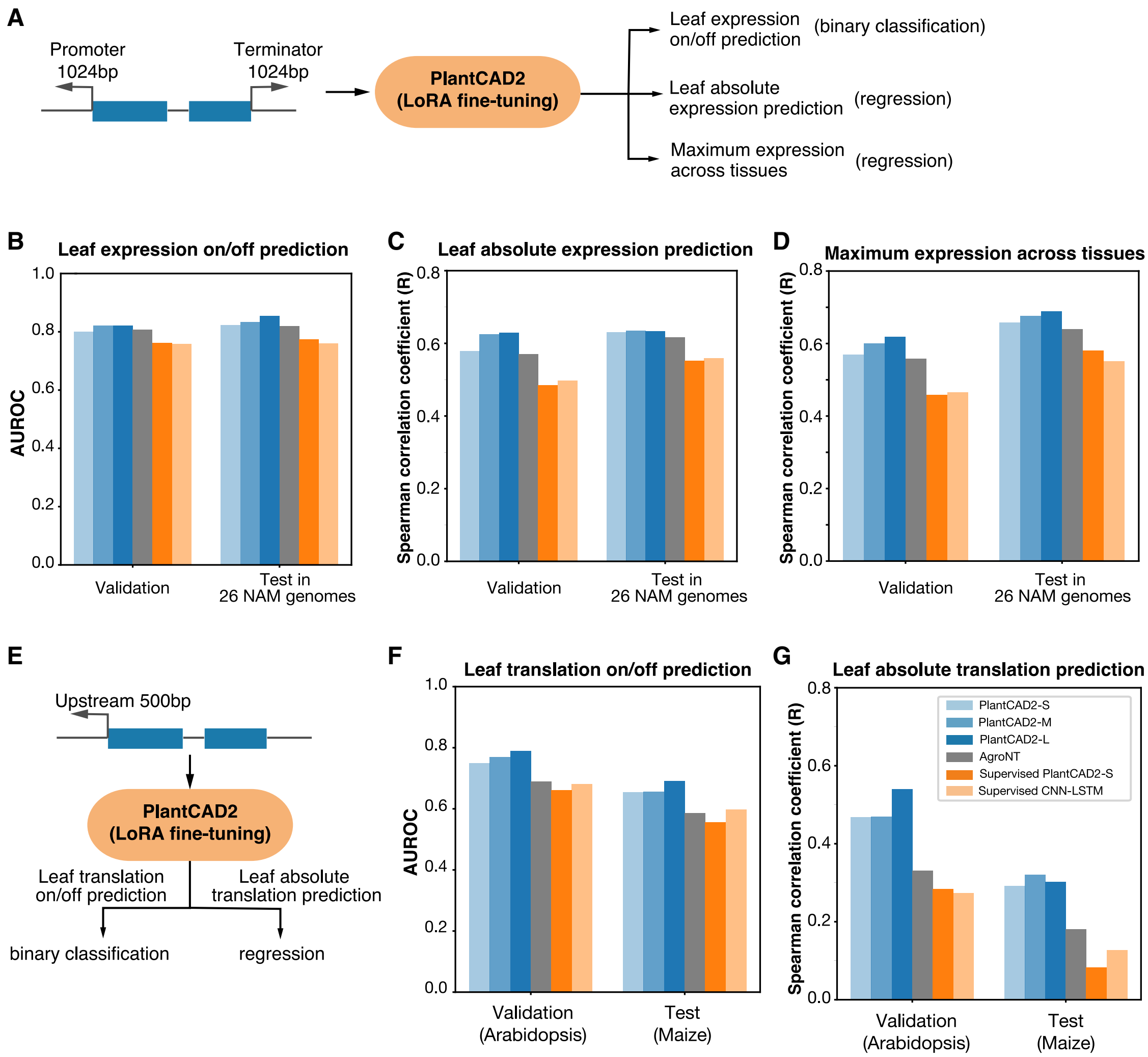

Figure 5. PlantCAD2 predicts gene expression and translation across species

(A) Gene-expression prediction pipeline using promoter and terminator sequences (1,024 bp each) for binary classification and regression tasks.

(B–D) Cross-species gene-expression performance on maize NAM population for binary on/off prediction (B), absolute expression levels (C), and maximum gene expression across tissues (D).

(E) Translation prediction pipeline using 500-bp upstream sequences.

(F and G) Translation prediction performance trained on *Arabidopsis* and tested cross-species on maize for binary on/off prediction (F) and absolute translation levels (G).

representations can be effectively adapted to fine-grained regulatory annotations.

Fine-tuning PlantCAD2 predicts cross-species gene expression and protein abundance

To evaluate PlantCAD2's ability to capture gene regulatory signals, we fine-tuned PlantCAD2 models using LoRA for two complementary tasks: gene expression and leaf translation (Figures 5A and 5E). Each task involved both classification (on/off status) and regression (absolute expression/translation level) objectives. For gene expression, we used promoter and terminator sequences (1,024 bp each) as input; for translation, we used 500-bp upstream sequences. Following the same strategy as the accessible chromatin prediction task, we fine-tuned PlantCAD2 with LoRA and compared its performance with supervised PlantCAD2-S, CNN + LSTM, and AgroNT.

For cross-species gene-expression modeling, we fine-tuned PlantCAD2 on a diverse panel of 15 plant species and evaluated predictions in 26 maize nested association mapping (NAM) genomes[71] (STAR Methods). We used three tasks to evaluate different models' performance to predict gene expression: (1) a binary classification task to predict whether a gene is expressed or not expressed in leaf tissue; (2) a regression task to predict the absolute expression in leaf tissue; (3) a regression task to predict the maximum expression across tissues, which represents a gene's maximal transcriptional potential and avoids dilution from inactive tissues (Figure 5A). Across these tasks, PlantCAD2 consistently outperformed established baselines

such as AgroNT and supervised CNN + LSTM. When testing in 26 NAM genomes, even the smallest PlantCAD2 (PlantCAD2-S, 88M parameters) outperformed the performance of the much larger AgroNT model (1 billion parameters) (Figures 5B–5D; Table S9), demonstrating the efficiency of our foundation model framework. The largest model, PlantCAD2-L, achieved the best AUROC for binary leaf expression and the highest Spearman correlation for absolute expression prediction. To also rigorously assess predictive fit for regression tasks (tasks 2 and 3), we also reported Pearson correlation, R^2, mean absolute error (MAE), and mean squared error (MSE) (Table S9), observing consistent model performance rankings across these metrics. To further validate the model's generalization capabilities beyond maize, we extended our evaluation to rice and tomato, where PlantCAD2 maintained the best cross-species performance (Figure S12).

Given that regulatory information also lies outside the proximal promoter,[37] we evaluated the effect of varying input window sizes on gene-expression prediction. Increasing the window from 1 to 4 kb both upstream of the transcription start site and downstream of the transcription stop site resulted in measurable improvements, raising the AUROC from 0.8221 to 0.8455 for binary leaf expression task and Spearman correlation from 0.6296 to 0.6455 for absolute expression prediction task on the NAM test set (Figure S13). To confirm that these improvements represent a true biological signal rather than stochastic variance, we performed repeated evaluations with different random seeds. The results exhibited high stability with minimal standard deviation (average SD = 0.0025 for binary leaf expression task and SD = 0.0032 for absolute expression prediction task; Table S10; Figure S13), confirming that the performance gain from extended context—though numerically modest—is statistically significant and consistently reproducible. These improvements highlight the role of distal enhancers and long-range motifs in shaping expression. However, previous studies in both humans and plants have shown that current deep-learning models lack the resolution to capture allele-specific effects.[71–73] We therefore tested whether fine-tuning a foundation model could mitigate this limitation by evaluating per-orthogroup correlations within the NAM population, a comparison sensitive to allelic differences. Consistent with prior findings,[71–73] only marginal improvements were observed for leaf absolute expression prediction, with the median Spearman correlation increasing from 0.112 (supervised CNN + LSTM) to 0.140 (PlantCAD2-S) (Figure S14). These small gains likely reflect both the high turnover of *cis*-regulatory elements[74] and limited availability of curated datasets with partitioned *cis/trans* effects.[75,76] Achieving robust allele-specific prediction will likely require explicit *cis*-variant modeling (e.g., VariantFormer[77]) or fine-tuning on curated regulatory variant datasets.[75,78]

Translation prediction was based on ribosome profiling (ribo-seq), a sequencing-based approach that estimates translation activity by mapping ribosome-protected mRNA fragments. Given high-quality ribo-seq datasets are scarce in plants, we restricted training to *Arabidopsis* and tested both within-species performance and cross-species transfer to maize. Interestingly, although supervised PlantCAD2-S (88M) is much larger than the CNN-LSTM (∼1.7 million), the latter performed better, suggesting that large supervised models are prone to overfitting when trained on limited data (Figures 5F and 5G; Table S11). By contrast, fine-tuned PlantCAD2 with parameter-efficient LoRA maintained robust performance without signs of overfitting. However, cross-species regression of absolute translation levels was less effective (Figure 5G), likely due to noise inherent in current ribo-seq-based quantitative estimates. We anticipate that, as more high-quality cross-species ribo-seq datasets become available, fine-tuning will demonstrate significantly improved robustness for quantitative prediction. Despite this limitation, we were encouraged by the strong transfer observed for binary classification of leaf translation. We next tested whether gene expression could similarly be transferred from *Arabidopsis* to maize. Using a separate *Arabidopsis* gene-expression dataset[79] with 1,024-bp upstream and downstream sequences, we found that direct transfer performed poorly (AUROC, 0.786 in *Arabidopsis* vs. 0.631 in maize) compared to protein abundance prediction (0.790 in *Arabidopsis* vs. 0.692 in maize) (Figure S15). This contrast may highlight a fundamental difference between regulatory layers: translational control appears to be more evolutionarily conserved than transcriptional regulation. Consistent with evidence that protein abundance is under stronger selective constraint than transcript levels,[62] these results explain why translation prediction transfers effectively across species, whereas accurate gene-expression prediction requires phylogenetically diverse training data.

DISCUSSION

In this work, we present PlantCAD2, an extended-context window DNA LM that substantially advances the sequence-to-function modeling in plant genomics. Building on the foundation laid by PlantCAD, PlantCAD2 features a model architecture that is three times larger, a 16-fold longer context window (8,192 vs. 512 bp), and a pre-training dataset that is evenly distributed across angiosperm orders to better capture phylogenetic diversity. Through comprehensive zero-shot and fine-tuned evaluations, we demonstrate that PlantCAD2 not only exhibits strong cross-species generalization but also achieves superior performance across a wide range of sequence-to-function tasks, including evolutionary conservation, junction site prediction, variant effect prediction, chromatin accessibility, gene expression, and protein translation.

A key design consideration for PlantCAD2 is the careful curation of one representative genome per genus, which avoids both the diminishing returns of scaling training data without proportionally increasing model capacity[80] and the well-documented bias toward overrepresented lineages in genomic,[81] NLP training corpora[82,83] and protein LMs.[84] More importantly, phylogenetic diversity matters more than absolute species count.[35] For example, NTv2,[25] trained on a few hundred diverse species, outperforms models trained on 1,000 human genomes. This principle also explains why generalist models such as Evo2 and NTv3,[85] despite spanning the entire tree of life, show substantially lower zero-shot performance than PlantCAD2 across all 12 plant benchmarks (Figure S16). Fine-tuning can compensate for weaker lineage-specific representations, but zero-shot performance more directly reflects pre-trained representation

quality for a given clade. By focusing on angiosperms, which diverged ~130–150 million years ago[86] and share a coherent regulatory grammar, PlantCAD2 captures plant-specific constraints without noise from unrelated genomic architectures.

The 8,192-bp context window reflects a deliberate balance between biological resolution and computational feasibility. Although distal regulatory interactions in plants can extend over tens of kilobases,[37] the median gene length of the 65 pre-training genomes is 2,422 bp (Figure S17). Regions far beyond this range are frequently dominated by TEs rather than functional *cis*-regulatory modules,[87] reducing their contribution to prediction accuracy. From a technical perspective, longer windows (e.g., 16–32 kb) remain costly even with linear-complexity architectures,[36,41] although PlantCAD2's flexible chunking strategies can integrate distal information across adjacent windows when needed.

PlantCAD2 represents a significant step toward a foundational model for plant genomics. Rather than building task-specific models for each application, it enables unified modeling of sequence-to-function relationships that can be efficiently adapted across cell types, tissues, and species. Fine-tuning PlantCAD2 enables accurate *de novo* genome annotation,[88] further expanding its utility for genome interpretation. For example, PlantCAD2 could assist in prioritizing causal variants in genome-wide association studies (GWASs), interpreting SVs in non-coding regions, or guiding sequence design for synthetic promoters with desired expression patterns. Its ability to transfer knowledge across evolutionarily distant species further enhances its utility for crop improvement, particularly in non-model organisms where high-quality training data are limited but genomic sequences are available.

Limitations of the study

Despite its advances, several limitations should be considered when interpreting the results of this study. PlantCAD2's model size may limit deployment in GPU-constrained environments. To directly assess the lower bound of model size, we additionally trained and evaluated a substantially smaller PlantCAD2 model (~20 million parameters) using the same pre-training data and zero-shot evaluation protocols. In contrast to the PlantCAD2-S, this lightweight model showed a pronounced drop in performance across most tasks (Table S12). For example, in predicting evolutionary conservation within Andropogoneae, the AUROC decreased from 0.725 (PlantCAD2-L) to 0.593 (20 million model), indicating a clear capacity threshold below which biologically meaningful sequence representations cannot be reliably learned. These results suggest that 88 million parameters is near the minimal viable size, and further reductions would require distillation or compression strategies. Second, there are still technical barriers that may limit accessibility to wet-lab biologists or breeders. Building intuitive interfaces and end-to-end pipelines will be important for broader adoption. Third, while the 8,192-bp context window allows PlantCAD2 to model distal regulatory elements, further extending this capability would be valuable for capturing long-range interactions such as enhancer-promoter loops. For example, in maize, the teosinte branched 1 (*tb1*) enhancer[89,90] and vegetative to generative transition 1 (*Vgt1*)[91] are located approximately 70 and 60 kb upstream of their target genes, respectively. However, capturing such interactions will likely require novel tokenization or compression strategies that can represent long, repetitive sequences without sacrificing resolution. Additionally, our zero-shot conservation evaluations rely on alignment-derived metrics (phyloP, phastCons, GERP) as ground truth, which carry inherent biases in non-coding and repetitive regions where plant genome alignments are less reliable. Furthermore, while PlantCAD2 demonstrates strong cross-species transfer for chromatin accessibility and gene expression, allele-specific expression prediction remains limited, consistent with prior findings across deep-learning models in both plants and humans. Finally, all fine-tuning evaluations were conducted using a single epoch without hyperparameter optimization to ensure reproducibility, which may underestimate the model's full task-specific performance.

Looking forward, future directions include combining PlantCAD2 with multi-modal data such as DNA methylation and chromatin states, which will provide *trans* factors to the genome. In addition, diffusion-based sequence generation models could also be promising coupled with synthetic biology. Ultimately, we envision PlantCAD2 and its successors as key building blocks for a sequence-to-function foundation model capable of enabling predictive genomics and rational genome design in diverse plant species.

RESOURCE AVAILABILITY

Lead contact

Requests for further information and resources should be directed to and will be fulfilled by the lead contact, Jingjing Zhai (jz963@cornell.edu).

Materials availability

This study did not generate new unique reagents.

Data and code availability

- All pre-trained models, datasets, and benchmark tasks are available at https://huggingface.co/collections/kuleshov-group/plantcad2-67e437e241a382671371a572.
- Code for pre-training, fine-tuning, and evaluation is deposited at Zenodo (DOI: https://doi.org/10.5281/zenodo.20971052) and https://github.com/plantcad/plantcad.

ACKNOWLEDGMENTS

This work is funded by a cooperative agreement from the US Department of Agriculture Agricultural Research Service to Cornell University (M.C.R.), a National Science Foundation (NSF) grant (#2240888), an NSF CAREER grant (#2145577), and NIH Maximizing Investigators' Research Awards (#1R35GM151243-01 and 5R35GM151348). We thank Travis Wrightsman (Cornell University) for sharing gene-expression tasks, Michelle C. Stitzer (Cornell University) for plotting the tree, Hai Wang (China Agricultural University) for sharing max expression data of 17 species, Yaoyao Wu (Nanjing Agricultural University) for providing conserved and neutral SNP datasets, Sara Miller (Cornell University) for helpful comments, and all members of Cornell's Institute of Genomic Diversity (M.C.R. and E.S.B.) for helpful discussions. We would also like to thank the SCINet project, Texas Advanced Computing Center at The University of Texas at Austin, and MosaicML for providing compute resources for pre-training and fine-tuning experiments.

AUTHOR CONTRIBUTIONS

J.Z., A.G., V.K., and E.S.B. designed research. J.Z., A.G., S.-K.H., Z.-Y.L., S.-P.C., E.M., E.C., B.C., A.B., M.C.R., M.P., V.K., and E.S.B. performed research and analyses. J.Z., E.M., S.-K.H., M.P., and E.S.B. wrote the manuscript with all other authors' suggestions and comments.

DECLARATION OF INTERESTS

The authors declare no competing interests.

DECLARATION OF GENERATIVE AI AND AI-ASSISTED TECHNOLOGIES IN THE WRITING PROCESS

During the preparation of this manuscript, the authors used Claude (Anthropic) to assist with grammar checking. After using this tool, the authors reviewed and edited the content as needed and take full responsibility for the content of the publication.

STAR★METHODS

Detailed methods are provided in the online version of this paper and include the following:

- KEY RESOURCES TABLE
- EXPERIMENTAL MODEL AND STUDY PARTICIPANT DETAILS
- METHOD DETAILS
  - Preparing pre-training genomes
  - PlantCAD2 model architecture and pre-training
  - Repeat annotation and loss re-weighting
  - Evolutionary constraint prediction using the zero-shot strategy
  - Core and non-core gene classification using the zero-shot strategy
  - Accessible chromatin region prediction
  - Gene expression prediction in leaf
  - Leaf protein abundance prediction task
  - Fine-tuning PlantCAD2
  - Fine-tuning AgroNT
  - Supervised CNN + LSTM baseline
  - Supervised PlantCAD2 baseline
  - Zero-shot evaluation of PlantCAD2, PlantCAD, GPN, and NTv3 models
  - Zero-shot evaluation of Evo2 model
- QUANTIFICATION AND STATISTICAL ANALYSIS
- ADDITIONAL RESOURCES

SUPPLEMENTAL INFORMATION

Supplemental information can be found online at https://doi.org/10.1016/j.xgen.2026.101329.

Received: September 8, 2025
Revised: December 22, 2025
Accepted: July 16, 2026
Published: August 7, 2026

REFERENCES

1. Vancaester, E., and Blaxter, M. (2023). Phylogenomic analysis of Wolbachia genomes from the Darwin Tree of Life biodiversity genomics project. PLoS Biol. *21*, e3001972. https://doi.org/10.1371/journal.pbio.3001972.
2. Lewin, H.A., Robinson, G.E., Kress, W.J., Baker, W.J., Coddington, J., Crandall, K.A., Durbin, R., Edwards, S.V., Forest, F., Gilbert, M.T.P., et al. (2018). Earth BioGenome Project: Sequencing life for the future of life. Proc. Natl. Acad. Sci. USA *115*, 4325–4333. https://doi.org/10.1073/pnas.1720115115.
3. Rhie, A., McCarthy, S.A., Fedrigo, O., Damas, J., Formenti, G., Koren, S., Uliano-Silva, M., Chow, W., Fungtammasan, A., Kim, J., et al. (2021). Towards complete and error-free genome assemblies of all vertebrate species. Nature *592*, 737–746. https://doi.org/10.1038/s41586-021-03451-0.
4. Cheng, S., Melkonian, M., Smith, S.A., Brockington, S., Archibald, J.M., Delaux, P.-M., Li, F.-W., Melkonian, B., Mavrodiev, E.V., Sun, W., et al. (2018). 10KP: A phylodiverse genome sequencing plan. GigaScience *7*, 1–9. https://doi.org/10.1093/gigascience/giy013.
5. Sun, Y., Shang, L., Zhu, Q.-H., Fan, L., and Guo, L. (2022). Twenty years of plant genome sequencing: achievements and challenges. Trends Plant Sci. *27*, 391–401. https://doi.org/10.1016/j.tplants.2021.10.006.
6. Fu, L.-Y., Zhu, T., Zhou, X., Yu, R., He, Z., Zhang, P., Wu, Z., Chen, M., Kaufmann, K., and Chen, D. (2022). ChIP-Hub provides an integrative platform for exploring plant regulome. Nat. Commun. *13*, 3413. https://doi.org/10.1038/s41467-022-30770-1.
7. Magallón, S., and Castillo, A. (2009). Angiosperm diversification through time. Am. J. Bot. *96*, 349–365. https://doi.org/10.3732/ajb.0800060.
8. Bar-On, Y.M., Phillips, R., and Milo, R. (2018). The biomass distribution on Earth. Proc. Natl. Acad. Sci. USA *115*, 6506–6511. https://doi.org/10.1073/pnas.1711842115.
9. Bommasani, R., Hudson, D.A., Adeli, E., Altman, R., Arora, S., von Arx, S., Bernstein, M.S., Bohg, J., Bosselut, A., Brunskill, E., et al. (2021). On the opportunities and risks of foundation models. Preprint at arXiv. https://doi.org/10.48550/arXiv.2108.07258.
10. Rives, A., Meier, J., Sercu, T., Goyal, S., Lin, Z., Liu, J., Guo, D., Ott, M., Zitnick, C.L., Ma, J., and Fergus, R. (2021). Biological structure and function emerge from scaling unsupervised learning to 250 million protein sequences. Proc. Natl. Acad. Sci. USA *118*, e2016239118. https://doi.org/10.1073/pnas.2016239118.
11. Lin, Z., Akin, H., Rao, R., Hie, B., Zhu, Z., Lu, W., Smetanin, N., Verkuil, R., Kabeli, O., Shmueli, Y., et al. (2023). Evolutionary-scale prediction of atomic-level protein structure with a language model. Science *379*, 1123–1130. https://doi.org/10.1126/science.ade2574.
12. Hayes, T., Rao, R., Akin, H., Sofroniew, N.J., Oktay, D., Lin, Z., Verkuil, R., Tran, V.Q., Deaton, J., Wiggert, M., et al. (2025). Simulating 500 million years of evolution with a language model. Science *387*, 850–858. https://doi.org/10.1126/science.ads0018.
13. Elnaggar, A., Heinzinger, M., Dallago, C., Rehawi, G., Wang, Y., Jones, L., Gibbs, T., Feher, T., Angerer, C., Steinegger, M., et al. (2022). ProtTrans: Toward Understanding the Language of Life Through Self-Supervised Learning. IEEE Trans. Pattern Anal. Mach. Intell. *44*, 7112–7127. https://doi.org/10.1109/TPAMI.2021.3095381.
14. Brandes, N., Ofer, D., Peleg, Y., Rappoport, N., and Linial, M. (2022). ProteinBERT: a universal deep-learning model of protein sequence and function. Bioinformatics *38*, 2102–2110. https://doi.org/10.1093/bioinformatics/btac020.
15. Kulmanov, M., Guzmán-Vega, F.J., Duek Roggli, P., Lane, L., Arold, S.T., and Hoehndorf, R. (2024). Protein function prediction as approximate semantic entailment. Nat. Mach. Intell. *6*, 220–228. https://doi.org/10.1038/s42256-024-00795-w.
16. Chowdhury, R., Bouatta, N., Biswas, S., Floristean, C., Kharkar, A., Roy, K., Rochereau, C., Ahdritz, G., Zhang, J., Church, G.M., et al. (2022). Single-sequence protein structure prediction using a language model and deep learning. Nat. Biotechnol. *40*, 1617–1623. https://doi.org/10.1038/s41587-022-01432-w.
17. Brandes, N., Goldman, G., Wang, C.H., Ye, C.J., and Ntranos, V. (2023). Genome-wide prediction of disease variant effects with a deep protein language model. Nat. Genet. *55*, 1512–1522. https://doi.org/10.1038/s41588-023-01465-0.
18. Nguyen, E., Poli, M., Durrant, M.G., Kang, B., Katrekar, D., Li, D.B., Bartie, L.J., Thomas, A.W., King, S.H., Brixi, G., et al. (2024). Sequence

modeling and design from molecular to genome scale with Evo. Science *386*, eado9336. https://doi.org/10.1126/science.ado9336.

19. Brixi, G., Durrant, M.G., Ku, J., Naghipourfar, M., Poli, M., Sun, G., Brockman, G., Chang, D., Fanton, A., Gonzalez, G.A., et al. (2026). Genome modelling and design across all domains of life with Evo 2. Nature *652*, 1349–1361. https://doi.org/10.1038/s41586-026-10176-5.
20. Ji, Y., Zhou, Z., Liu, H., and Davuluri, R.V. (2021). DNABERT: pre-trained Bidirectional Encoder Representations from Transformers model for DNA-language in genome. Bioinformatics *37*, 2112–2120. https://doi.org/10.1093/bioinformatics/btab083.
21. Zhou, Z., Ji, Y., Li, W., Dutta, P., Davuluri, R.V., and Liu, H. (2023). DNABERT-2: Efficient Foundation Model and Benchmark For Multi-Species Genomes. Preprint at arXiv. https://doi.org/10.48550/arXiv.2306.15006.
22. Benegas, G., Batra, S.S., and Song, Y.S. (2023). DNA language models are powerful predictors of genome-wide variant effects. Proc. Natl. Acad. Sci. USA *120*, e2311219120. https://doi.org/10.1073/pnas.2311219120.
23. Benegas, G., Albors, C., Aw, A.J., Ye, C., and Song, Y.S. (2025). A DNA language model based on multispecies alignment predicts the effects of genome-wide variants. Nat. Biotechnol. *43*, 1960–1965. https://doi.org/10.1038/s41587-024-02511-w.
24. Mendoza-Revilla, J., Trop, E., Gonzalez, L., Roller, M., Dalla-Torre, H., de Almeida, B.P., Richard, G., Caton, J., Lopez Carranza, N., Skwark, M., et al. (2024). A foundational large language model for edible plant genomes. Commun. Biol. *7*, 835. https://doi.org/10.1038/s42003-024-06465-2.
25. Dalla-Torre, H., Gonzalez, L., Mendoza-Revilla, J., Lopez Carranza, N., Grzywaczewski, A.H., Oteri, F., Dallago, C., Trop, E., de Almeida, B.P., Sirelkhatim, H., et al. (2025). Nucleotide Transformer: building and evaluating robust foundation models for human genomics. Nat. Methods *22*, 287–297. https://doi.org/10.1038/s41592-024-02523-z.
26. Schiff, Y., Kao, C.-H., Gokaslan, A., Dao, T., Gu, A., and Kuleshov, V. (2024). Caduceus: Bi-Directional Equivariant Long-Range DNA Sequence Modeling. Preprint at arXiv. https://doi.org/10.48550/arXiv.2403.03234.
27. Zhai, J., Gokaslan, A., Schiff, Y., Berthel, A., Liu, Z.-Y., Lai, W.-Y., Miller, Z.R., Scheben, A., Stitzer, M.C., Romay, M.C., et al. (2025). Cross-species modeling of plant genomes at single-nucleotide resolution using a pretrained DNA language model. Proc. Natl. Acad. Sci. USA *122*, e2421738122. https://doi.org/10.1073/pnas.2421738122.
28. Wang, N., Bian, J., Li, Y., Li, X., Mumtaz, S., Kong, L., and Xiong, H. (2024). Multi-purpose RNA language modelling with motif-aware pretraining and type-guided fine-tuning. Nat. Mach. Intell. *6*, 548–557. https://doi.org/10.1038/s42256-024-00836-4.
29. Shen, T., Hu, Z., Sun, S., Liu, D., Wong, F., Wang, J., Chen, J., Wang, Y., Hong, L., Xiao, J., et al. (2024). Accurate RNA 3D structure prediction using a language model-based deep learning approach. Nat. Methods *21*, 2287–2298. https://doi.org/10.1038/s41592-024-02487-0.
30. Yu, H., Yang, H., Sun, W., Yan, Z., Yang, X., Zhang, H., Ding, Y., and Li, K. (2024). An interpretable RNA foundation model for exploring functional RNA motifs in plants. Nat. Mach. Intell. *6*, 1616–1625. https://doi.org/10.1038/s42256-024-00946-z.
31. Hao, M., Gong, J., Zeng, X., Liu, C., Guo, Y., Cheng, X., Wang, T., Ma, J., Zhang, X., and Song, L. (2024). Large-scale foundation model on single-cell transcriptomics. Nat. Methods *21*, 1481–1491. https://doi.org/10.1038/s41592-024-02305-7.
32. Zeng, Y., Xie, J., Shangguan, N., Wei, Z., Li, W., Su, Y., Yang, S., Zhang, C., Zhang, J., Fang, N., et al. (2025). CellFM: a large-scale foundation model pre-trained on transcriptomics of 100 million human cells. Nat. Commun. *16*, 4679. https://doi.org/10.1038/s41467-025-59926-5.
33. Cui, H., Wang, C., Maan, H., Pang, K., Luo, F., Duan, N., and Wang, B. (2024). scGPT: toward building a foundation model for single-cell multi-omics using generative AI. Nat. Methods *21*, 1470–1480. https://doi.org/10.1038/s41592-024-02201-0.
34. Devlin, J., Chang, M.-W., Lee, K., and Toutanova, K. (2018). BERT: Pre-training of Deep Bidirectional Transformers for Language Understanding. Preprint at arXiv. https://doi.org/10.48550/arXiv.1810.04805.
35. Benegas, G., Ye, C., Albors, C., Li, J.C., and Song, Y.S. (2025). Genomic language models: opportunities and challenges. Trends Genet. *41*, 286–302. https://doi.org/10.1016/j.tig.2024.11.013.
36. Gu, A., and Dao, T. (2023). Mamba: Linear-Time Sequence Modeling with Selective State Spaces. Preprint at arXiv. https://doi.org/10.48550/arXiv.2312.00752.
37. Schmitz, R.J., Grotewold, E., and Stam, M. (2022). Cis-regulatory sequences in plants: Their importance, discovery, and future challenges. Plant Cell *34*, 718–741. https://doi.org/10.1093/plcell/koab281.
38. Marand, A.P., Eveland, A.L., Kaufmann, K., and Springer, N.M. (2023). Cis-regulatory elements in plant development, adaptation, and evolution. Annu. Rev. Plant Biol. *74*, 111–137. https://doi.org/10.1146/annurev-arplant-070122-030236.
39. Engelhorn, J., Snodgrass, S.J., Kok, A., Seetharam, A.S., Schneider, M., Kiwit, T., Singh, A., Banf, M., Doan, D.T.H., Khaipho-Burch, M., et al. (2025). Genetic variation at transcription factor binding sites largely explains phenotypic heritability in maize. Nat. Genet. *57*, 2313–2322. https://doi.org/10.1038/s41588-025-02246-7.
40. Marand, A.P., Jiang, L., Gomez-Cano, F., Minow, M.A.A., Zhang, X., Mendieta, J.P., Luo, Z., Bang, S., Yan, H., Meyer, C., et al. (2025). The genetic architecture of cell type-specific cis regulation in maize. Science *388*, eads6601. https://doi.org/10.1126/science.ads6601.
41. Dao, T., and Gu, A. (2024). Transformers are SSMs: Generalized models and efficient algorithms through structured state space duality. Preprint at arXiv. https://doi.org/10.48550/arXiv.2405.21060.
42. Vaswani, A., Shazeer, N., Parmar, N., Uszkoreit, J., Jones, L., Gomez, A.N., Kaiser, L., and Polosukhin, I. (2017). Attention Is All You Need. Preprint at arXiv. https://doi.org/10.48550/arXiv.1706.03762.
43. Warner, B., Chaffin, A., Clavié, B., Weller, O., Hallström, O., Taghadouini, S., Gallagher, A., Biswas, R., Ladhak, F., Aarsen, T., et al. (2024). Smarter, better, faster, longer: A modern bidirectional encoder for fast, memory efficient, and long context finetuning and inference. Preprint at arXiv. https://doi.org/10.48550/arXiv.2412.13663.
44. Gu, A., Goel, K., and Ré, C. (2021). Efficiently Modeling Long Sequences with Structured State Spaces. Preprint at arXiv. https://doi.org/10.48550/arXiv.2111.00396.
45. Novák, P., Guignard, M.S., Neumann, P., Kelly, L.J., Mlinarec, J., Koblížková, A., Dodsworth, S., Kovařík, A., Pellicer, J., Wang, W., et al. (2020). Repeat-sequence turnover shifts fundamentally in species with large genomes. Nat. Plants *6*, 1325–1329. https://doi.org/10.1038/s41477-020-00785-x.
46. Sun, S., Wang, B., Li, C., Xu, G., Yang, J., Hufford, M.B., Ross-Ibarra, J., Wang, H., and Wang, L. (2023). Unraveling Prevalence and Effects of Deleterious Mutations in Maize Elite Lines across Decades of Modern Breeding. Mol. Biol. Evol. *40*, msad170. https://doi.org/10.1093/molbev/msad170.
47. Lozano, R., Gazave, E., dos Santos, J.P.R., Stetter, M.G., Valluru, R., Bandillo, N., Fernandes, S.B., Brown, P.J., Shakoor, N., Mockler, T.C., et al. (2021). Comparative evolutionary genetics of deleterious load in sorghum and maize. Nat. Plants *7*, 17–24. https://doi.org/10.1038/s41477-020-00834-5.
48. Lye, Z., Choi, J.Y., and Purugganan, M.D. (2022). Deleterious Mutations and the Rare Allele Burden on Rice Gene Expression. Mol. Biol. Evol. *39*, msac193. https://doi.org/10.1093/molbev/msac193.
49. Mezmouk, S., and Ross-Ibarra, J. (2014). The pattern and distribution of deleterious mutations in maize. G3 *4*, 163–171. https://doi.org/10.1534/g3.113.008870.

50. Song, B., Buckler, E.S., and Stitzer, M.C. (2024). New whole-genome alignment tools are needed for tapping into plant diversity. Trends Plant Sci. *29*, 355–369. https://doi.org/10.1016/j.tplants.2023.08.013.

51. Huber, C.D., Kim, B.Y., and Lohmueller, K.E. (2020). Population genetic models of GERP scores suggest pervasive turnover of constrained sites across mammalian evolution. PLoS Genet. *16*, e1008827. https://doi.org/10.1371/journal.pgen.1008827.

52. Bennetzen, J.L., and Wang, H. (2014). The contributions of transposable elements to the structure, function, and evolution of plant genomes. Annu. Rev. Plant Biol. *65*, 505–530. https://doi.org/10.1146/annurev-arplant-050213-035811.

53. Kozak, M. (1986). Point mutations define a sequence flanking the AUG initiator codon that modulates translation by eukaryotic ribosomes. Cell *44*, 283–292. https://doi.org/10.1016/0092-8674(86)90762-2.

54. Davydov, E.V., Goode, D.L., Sirota, M., Cooper, G.M., Sidow, A., and Batzoglou, S. (2010). Identifying a high fraction of the human genome to be under selective constraint using GERP++. PLoS Comput. Biol. *6*, e1001025. https://doi.org/10.1371/journal.pcbi.1001025.

55. Wu, Y., Li, D., Hu, Y., Li, H., Ramstein, G.P., Zhou, S., Zhang, X., Bao, Z., Zhang, Y., Song, B., et al. (2023). Phylogenomic discovery of deleterious mutations facilitates hybrid potato breeding. Cell *186*, 2313–2328.e15. https://doi.org/10.1016/j.cell.2023.04.008.

56. Li, Y.-R., and Liu, M.-J. (2020). Prevalence of alternative AUG and non-AUG translation initiators and their regulatory effects across plants. Genome Res. *30*, 1418–1433. https://doi.org/10.1101/gr.261834.120.

57. Tian, F., Yang, D.-C., Meng, Y.-Q., Jin, J., and Gao, G. (2020). PlantRegMap: charting functional regulatory maps in plants. Nucleic Acids Res. *48*, D1104–D1113. https://doi.org/10.1093/nar/gkz1020.

58. Hubisz, M.J., Pollard, K.S., and Siepel, A. (2011). PHAST and RPHAST: phylogenetic analysis with space/time models. Briefings Bioinform. *12*, 41–51. https://doi.org/10.1093/bib/bbq072.

59. O'Malley, R.C., Huang, S.-S.C., Song, L., Lewsey, M.G., Bartlett, A., Nery, J.R., Galli, M., Gallavotti, A., and Ecker, J.R. (2016). Cistrome and epicistrome features shape the regulatory DNA landscape. Cell *165*, 1280–1292.

60. Shrikumar, A., Tian, K., Avsec, Ž., Shcherbina, A., Banerjee, A., Sharmin, M., Nair, S., and Kundaje, A. (2018). Technical note on Transcription Factor Motif Discovery from Importance Scores (TF-MoDISco) version 0.5.6.5. Preprint at arXiv. https://doi.org/10.48550/arXiv.1811.00416.

61. Kerstens, M.H.L., Schranz, M.E., and Bouwmeester, K. (2020). Phylogenomic analysis of the APETALA2 transcription factor subfamily across angiosperms reveals both deep conservation and lineage-specific patterns. Plant J. *103*, 1516–1524. https://doi.org/10.1111/tpj.14843.

62. Kooiker, M., Airoldi, C.A., Losa, A., Manzotti, P.S., Finzi, L., Kater, M.M., and Colombo, L. (2005). BASIC PENTACYSTEINE1, a GA binding protein that induces conformational changes in the regulatory region of the homeotic Arabidopsis gene SEEDSTICK. Plant Cell *17*, 722–729. https://doi.org/10.1105/tpc.104.030130.

63. Liu, Z., Running, M.P., and Meyerowitz, E.M. (1997). TSO1 functions in cell division during Arabidopsis flower development. Development *124*, 665–672. https://doi.org/10.1242/dev.124.3.665.

64. Fujii, S., Yamamoto, E., Ito, S., Tangpranomkorn, S., Kimura, Y., Miura, H., Yamaguchi, N., Kato, Y., Niidome, M., Yoshida, A., et al. (2023). SHI family transcription factors regulate an interspecific barrier. Nat. Plants *9*, 1862–1873. https://doi.org/10.1038/s41477-023-01535-5.

65. Shaikhali, J., de Dios Barajas-Lopéz, J., Ötvös, K., Kremnev, D., Garcia, A.S., Srivastava, V., Wingsle, G., Bako, L., and Strand, Å. (2012). The CRYPTOCHROME1-dependent response to excess light is mediated through the transcriptional activators ZINC FINGER PROTEIN EXPRESSED IN INFLORESCENCE MERISTEM LIKE1 and ZML2 in Arabidopsis. Plant Cell *24*, 3009–3025. https://doi.org/10.1105/tpc.112.100099.

66. Lu, Z., Marand, A.P., Ricci, W.A., Ethridge, C.L., Zhang, X., and Schmitz, R.J. (2019). The prevalence, evolution and chromatin signatures of plant regulatory elements. Nat. Plants *5*, 1250–1259. https://doi.org/10.1038/s41477-019-0548-z.

67. Hu, E.J., Shen, Y., Wallis, P., Allen-Zhu, Z., Li, Y., Wang, S., Wang, L., and Chen, W. (2021). LoRA: Low-Rank Adaptation of large language models. Preprint at arXiv. https://doi.org/10.48550/arXiv.2106.09685.

68. Quang, D., and Xie, X. (2016). DanQ: a hybrid convolutional and recurrent deep neural network for quantifying the function of DNA sequences. Nucleic Acids Res. *44*, e107. https://doi.org/10.1093/nar/gkw226.

69. Chen, T., and Guestrin, C. (2016). XGBoost: A Scalable Tree Boosting System. Preprint at arXiv. https://doi.org/10.48550/arXiv.1603.02754.

70. Marand, A.P., Chen, Z., Gallavotti, A., and Schmitz, R.J. (2021). A cis-regulatory atlas in maize at single-cell resolution. Cell *184*, 3041–3055.e21. https://doi.org/10.1016/j.cell.2021.04.014.

71. Wrightsman, T., Ferebee, T.H., Romay, M.C., Seetharam, A.S., AuBuchon-Elder, T., Phillips, A.R., Syring, M., Hufford, M.B., Kellogg, E.A., and Buckler, E.S. (2024). Current genomic deep learning architectures generalize across grass species but not alleles. Preprint at bioRxiv. https://doi.org/10.1101/2024.04.11.589024.

72. Huang, C., Shuai, R.W., Baokar, P., Chung, R., Rastogi, R., Kathail, P., and Ioannidis, N.M. (2023). Personal transcriptome variation is poorly explained by current genomic deep learning models. Nat. Genet. *55*, 2056–2059. https://doi.org/10.1038/s41588-023-01574-w.

73. Tang, Z., Toneyan, S., and Koo, P.K. (2023). Current approaches to genomic deep learning struggle to fully capture human genetic variation. Nat. Genet. *55*, 2021–2022. https://doi.org/10.1038/s41588-023-01517-5.

74. Hale, C.O., Hsu, S.-K., Zhai, J., Schulz, A., Aubuchon-Elder, T., Costa-Neto, G., Gelfond, A., El-Walid, M.Z., Hufford, M., Kellogg, E.A., et al. (2026). Widespread turnover of a conserved cis-regulatory code across 589 grass species. Mol. Biol. Evol. *43*, msaf324. https://doi.org/10.1093/molbev/msaf324.

75. Jaganathan, K., Ersaro, N., Novakovsky, G., Wang, Y., James, T., Schwartzentruber, J., Fiziev, P., Kassam, I., Cao, F., Hawe, J., et al. (2025). Predicting expression-altering promoter mutations with deep learning. Science *389*, eads7373. https://doi.org/10.1126/science.ads7373.

76. Giri, A., Khaipho-Burch, M., Buckler, E.S., and Ramstein, G.P. (2021). Haplotype associated RNA expression (HARE) improves prediction of complex traits in maize. PLoS Genet. *17*, e1009568. https://doi.org/10.1371/journal.pgen.1009568.

77. Ghosal, S., Barhomi, Y., Ganapathi, T., Krystosik, A., Krishnan, L., Guntury, S., Li, D., Alzheimer's Disease Neuroimaging Initiative; Karaletsos, T., and Karaletsos, T. (2025). VariantFormer: A hierarchical transformer integrating DNA sequences with genetic variations and regulatory landscapes for personalized gene expression prediction. Preprint at bioRxiv. https://doi.org/10.1101/2025.10.31.685862.

78. Liu, T., Zhang, X., Ying, R., and Zhao, H. (2025). Pre-training genomic language model with variants for better modeling functional genomics. Preprint at bioRxiv. https://doi.org/10.1101/2025.02.26.640468.

79. Li, T., Xu, H., Teng, S., Suo, M., Bahitwa, R., Xu, M., Qian, Y., Ramstein, G.P., Song, B., Buckler, E.S., and Wang, H. (2024). Modeling 0.6 million genes for the rational design of functional cis-regulatory variants and de novo design of cis-regulatory sequences. Proc. Natl. Acad. Sci. USA *121*, e2319811121. https://doi.org/10.1073/pnas.2319811121.

80. Kaplan, J., McCandlish, S., Henighan, T., Brown, T.B., Chess, B., Child, R., Gray, S., Radford, A., Wu, J., and Amodei, D. (2020). Scaling laws for neural language models. Preprint at arXiv. https://doi.org/10.48550/arXiv.2001.08361.

81. Marks, R.A., Hotaling, S., Frandsen, P.B., and VanBuren, R. (2021). Representation and participation across 20 years of plant genome

CellPress
OPEN ACCESS

sequencing. Nat. Plants *7*, 1571–1578. https://doi.org/10.1038/s41477-021-01031-8.

82. Bender, E.M., Gebru, T., McMillan-Major, A., and Shmitchell, S. (2021). On the dangers of stochastic parrots: Can language models be too big? In Proceedings of the 2021 ACM Conference on Fairness, Accountability, and Transparency (ACM). https://doi.org/10.1145/3442188.3445922.
83. Geirhos, R., Jacobsen, J.-H., Michaelis, C., Zemel, R., Brendel, W., Bethge, M., and Wichmann, F.A. (2020). Shortcut learning in deep neural networks. Nat. Mach. Intell. *2*, 665–673. https://doi.org/10.1038/s42256-020-00257-z.
84. Ding, F., and Steinhardt, J. (2024). Protein language models are biased by unequal sequence sampling across the tree of life. Preprint at bioRxiv. https://doi.org/10.1101/2024.03.07.584001.
85. Boshar, S., Evans, B., Tang, Z., Picard, A., Adel, Y., Lorbeer, F.K., Rajesh, C., Karch, T., Sidbon, S., Emms, D., et al. (2025). A foundational model for joint sequence-function multi-species modeling at scale for long-range genomic prediction. Preprint at bioRxiv. https://doi.org/10.64898/2025.12.22.695963.
86. Smith, S.A., and Brown, J.W. (2018). Constructing a broadly inclusive seed plant phylogeny. Am. J. Bot. *105*, 302–314. https://doi.org/10.1002/ajb2.1019.
87. Stitzer, M.C., Anderson, S.N., Springer, N.M., and Ross-Ibarra, J. (2021). The genomic ecosystem of transposable elements in maize. PLoS Genet. *17*, e1009768. https://doi.org/10.1371/journal.pgen.1009768.
88. Liu, Z.-Y., Berthel, A., Czech, E., Marroquin, E., Stitzer, M., Hsu, S.K., Pennell, M., Buckler, E.S., and Zhai, J. (2025). GeneCAD: Plant genome annotation with a DNA foundation model. Preprint at bioRxiv. https://doi.org/10.1101/2025.10.31.685877.
89. Clark, R.M., Wagler, T.N., Quijada, P., and Doebley, J. (2006). A distant upstream enhancer at the maize domestication gene tb1 has pleiotropic effects on plant and inflorescent architecture. Nat. Genet. *38*, 594–597. https://doi.org/10.1038/ng1784.
90. Studer, A., Zhao, Q., Ross-Ibarra, J., and Doebley, J. (2011). Identification of a functional transposon insertion in the maize domestication gene tb1. Nat. Genet. *43*, 1160–1163. https://doi.org/10.1038/ng.942.
91. Tu, X., Mejía-Guerra, M.K., Valdes Franco, J.A., Tzeng, D., Chu, P.-Y., Shen, W., Wei, Y., Dai, X., Li, P., Buckler, E.S., and Zhong, S. (2020). Reconstructing the maize leaf regulatory network using ChIP-seq data of 104 transcription factors. Nat. Commun. *11*, 5089. https://doi.org/10.1038/s41467-020-18832-8.
92. Loshchilov, I., and Hutter, F. (2017). Decoupled Weight Decay Regularization. Preprint at arXiv. https://doi.org/10.48550/arXiv.1711.05101.
93. Song, B., Buckler, E.S., Wang, H., Wu, Y., Rees, E., Kellogg, E.A., Gates, D.J., Khaipho-Burch, M., Bradbury, P.J., Ross-Ibarra, J., et al. (2021). Conserved noncoding sequences provide insights into regulatory sequence and loss of gene expression in maize. Genome Res. *31*, 1245–1257. https://doi.org/10.1101/gr.266528.120.
94. Welker, C.A.D., McKain, M.R., Estep, M.C., Pasquet, R.S., Chipabika, G., Pallangyo, B., and Kellogg, E.A. (2020). Phylogenomics enables biogeographic analysis and a new subtribal classification of Andropogoneae (Poaceae—Panicoideae). J. Syst. Evol. *58*, 1003–1030. https://doi.org/10.1111/jse.12691.
95. Stitzer, M.C., Seetharam, A.S., Scheben, A., Hsu, S.-K., Schulz, A.J., AuBuchon-Elder, T.M., El-Walid, M., Ferebee, T.H., Hale, C.O., La, T., et al. (2025). Extensive genome evolution distinguishes maize within a stable tribe of grasses. Preprint at bioRxiv. https://doi.org/10.1101/2025.01.22.633974.
96. Song, B., Marco-Sola, S., Moreto, M., Johnson, L., Buckler, E.S., and Stitzer, M.C. (2022). AnchorWave: Sensitive alignment of genomes with high sequence diversity, extensive structural polymorphism, and whole-genome duplication. Proc. Natl. Acad. Sci. USA *119*, e2113075119. https://doi.org/10.1073/pnas.2113075119.
97. Pollard, K.S., Hubisz, M.J., Rosenbloom, K.R., and Siepel, A. (2010). Detection of nonneutral substitution rates on mammalian phylogenies. Genome Res. *20*, 110–121. https://doi.org/10.1101/gr.097857.109.
98. Hufford, M.B., Seetharam, A.S., Woodhouse, M.R., Chougule, K.M., Ou, S., Liu, J., Ricci, W.A., Guo, T., Olson, A., Qiu, Y., et al. (2021). De novo assembly, annotation, and comparative analysis of 26 diverse maize genomes. Science *373*, 655–662. https://doi.org/10.1126/science.abg5289.
99. Gao, L., Gonda, I., Sun, H., Ma, Q., Bao, K., Tieman, D.M., Burzynski-Chang, E.A., Fish, T.L., Stromberg, K.A., Sacks, G.L., et al. (2019). The tomato pan-genome uncovers new genes and a rare allele regulating fruit flavor. Nat. Genet. *51*, 1044–1051. https://doi.org/10.1038/s41588-019-0410-2.
100. Wrightsman, T., Marand, A.P., Crisp, P.A., Springer, N.M., and Buckler, E.S. (2022). Modeling chromatin state from sequence across angiosperms using recurrent convolutional neural networks. Plant Genome *15*, e20249. https://doi.org/10.1002/tpg2.20249.
101. Wu, H.-Y.L., Ai, Q., Teixeira, R.T., Nguyen, P.H.T., Song, G., Montes, C., Elmore, J.M., Walley, J.W., and Hsu, P.Y. (2024). Improved super-resolution ribosome profiling reveals prevalent translation of upstream ORFs and small ORFs in Arabidopsis. Plant Cell *36*, 510–539. https://doi.org/10.1093/plcell/koad290.
102. Zhu, W., Miao, X., Qian, J., Chen, S., Jin, Q., Li, M., Han, L., Zhong, W., Xie, D., Shang, X., and Li, L. (2023). A translatome-transcriptome multi-omics gene regulatory network reveals the complicated functional landscape of maize. Genome Biol. *24*, 60. https://doi.org/10.1186/s13059-023-02890-4.
103. Bolger, A.M., Lohse, M., and Usadel, B. (2014). Trimmomatic: a flexible trimmer for Illumina sequence data. Bioinformatics *30*, 2114–2120. https://doi.org/10.1093/bioinformatics/btu170.
104. Langmead, B., Trapnell, C., Pop, M., and Salzberg, S.L. (2009). Ultrafast and memory-efficient alignment of short DNA sequences to the human genome. Genome Biol. *10*, R25. https://doi.org/10.1186/gb-2009-10-3-r25.
105. Dobin, A., Davis, C.A., Schlesinger, F., Drenkow, J., Zaleski, C., Jha, S., Batut, P., Chaisson, M., and Gingeras, T.R. (2013). STAR: ultrafast universal RNA-seq aligner. Bioinformatics *29*, 15–21. https://doi.org/10.1093/bioinformatics/bts635.
106. Pertea, M., Pertea, G.M., Antonescu, C.M., Chang, T.-C., Mendell, J.T., and Salzberg, S.L. (2015). StringTie enables improved reconstruction of a transcriptome from RNA-seq reads. Nat. Biotechnol. *33*, 290–295. https://doi.org/10.1038/nbt.3122.
107. Mangrulkar, S., Gugger, S., Debut, L., Belkada, Y., Paul, S., and Bossan, B. (2022). Peft: State-of-the-art parameter-efficient fine-tuning methods. Preprint at arXiv. https://doi.org/10.48550/arXiv.2312.12148.

STAR★METHODS

KEY RESOURCES TABLE

REAGENT or RESOURCE	SOURCE	IDENTIFIER
Deposited data		
Pretraining genomes	Phytozome v14	https://huggingface.co/datasets/plantcad/Angiosperm_65_genomes_8192bp
Zero-shot tasks	This study	https://huggingface.co/datasets/plantcad/PlantCAD2_zero_shot_tasks; https://doi.org/10.5281/zenodo.20971052
Fine-tuning tasks	This study	https://huggingface.co/datasets/plantcad/PlantCAD2_fine_tuning_tasks; https://doi.org/10.5281/zenodo.20971052
Arabidopsis PhyloP and phastCons	Tian et al.[57]	http://plantregmap.gao-lab.org/download.php#alignment-conservation
DREB2 DAP-Seq	O'Malley et al.[59]	https://www.ncbi.nlm.nih.gov/geo/query/acc.cgi?acc=GSE60143
Software and algorithms		
PlantCAD2	This study	https://doi.org/10.5281/zenodo.20971052
PlantCAD	Zhai et al.[21]	https://huggingface.co/kuleshov-group/PlantCaduceus_l32
GPN	Benegas et al.[22]	https://github.com/songlab-cal/gpn
Evo2	Brixi et a.,[19]	https://github.com/arcinstitute/evo2
AgroNT	Mendoza-Revilla et al.[24]	https://github.com/instadeepai/nucleotide-transformer
Supervised CNN+LSTM	Quang et al.[68]	https://github.com/uci-cbcl/DanQ
TF-MoDISco	Shrikumar et al.[60]	https://github.com/jmschrei/tfmodisco-lite/

EXPERIMENTAL MODEL AND STUDY PARTICIPANT DETAILS

This study did not involve experimental organisms, human subjects, or cell lines. All analyses were performed on publicly available genome assemblies obtained from the Phytozome database (https://phytozome-next.jgi.doe.gov). Species information, including taxonomic classification and genome assembly details, is provided in Table S2.

METHOD DETAILS

Preparing pre-training genomes

A total of 65 genomes were selected for pre-training from the Phytozome database. To ensure taxonomic relevance and minimize redundancy, we applied a series of manual filtering steps. First, non-angiosperm species were excluded. For each remaining species, we retained only the most recent genome assembly version. In cases where two haplotypes were available for a species, we selected the haplotype with the higher N50 value; if N50 values were comparable, we retained the assembly with fewer scaffolds to prioritize less fragmented genomes. Taxonomic information, including order, family, and genus, was appended to each genome to facilitate downstream analyses, and their relationships were visualized using a published time-calibrated phylogeny.[86] For each selected genome, we extracted genomic sequences centered on each annotated gene, extending 5 kilobases (kb) upstream and 5 kb downstream of the gene center. These ±5 kb gene-centered regions were then segmented into overlapping windows of 8,192 bp with a step size of 4,096 bp, ensuring comprehensive coverage of regulatory and genic features while maintaining continuity across sequence boundaries. These windows served as input sequences for model pre-training.

PlantCAD2 model architecture and pre-training

PlantCAD2 builds upon the Caduceus architecture[26] used in PlantCAD,[27] retaining its key design principles while incorporating architectural improvements. Like PlantCAD1, PlantCAD2 maintains three core features: (1) bidirectional sequence processing, where sequences are processed both forward and reverse with outputs summed together; (2) reverse-complement (RC) equivariance, ensuring the model commutes with RC operations; and (3) parameter-efficient bidirectional implementation through shared linear projections between forward and reverse passes.

The primary architectural improvement in PlantCAD2 is the replacement of Mamba1 blocks[36] with Mamba2 blocks.[41] Mamba2 introduces a structured state space duality that recasts the selective state space computation into an equivalent convolutional form using structured matrices, improving parallelism and hardware efficiency. This dual representation enables significantly faster training (up to 2–4× in some scenarios) while retaining the input-dependent selection mechanism that allows the model to

dynamically modulate state updates based on sequence content. These advances allow PlantCAD2 to efficiently handle 8,192 bp sequences with linear computational complexity.

For the pre-training of PlantCAD2, each model was trained for 240,000 steps using a Decoupled AdamW optimizer[92] with the global batch size of 2,048. The learning rate is 2E-4 with a cosine decay scheduler, and 6% of the training duration was dedicated to warm up. The learning rate decayed to 4E-6 by the end of training. The default BERT[34] masking recipe was used with a masking probability of 15%. For each masked token: i) there is an 80% probability it will be replaced by a special token ([MASK]), ii) a 10% probability it will be replaced by a random token, and iii) a 10% probability it will remain unchanged. Unless otherwise specified, all models were trained using a sequence length of 8192 base pairs. A weight decay of 1E-5 was applied throughout the training process. Pre-training of the 676M-parameter PlantCAD2-L model required approximately 28 days on 64 NVIDIA H100 GPUs, while the 88M-parameter model completed training in approximately 3 days under the same hardware configuration.

Repeat annotation and loss re-weighting

To mitigate the influence of highly repetitive sequences during pre-training, we explicitly down-weighted the contribution of repetitive regions to the masked language modeling loss. This loss has been found in other DNA models[22,27] to improve performance on downstream tasks and better calibrate likelihoods between repetitive and nonrepetitive DNA. We employed a *de-novo* pipeline to annotate highly repetitive sequences[93] (https://github.com/baoxingsong/dCNS), then repetitive sequences are down-weighted during pre-training as demonstrated important in previous studies.[22] Then the loss is calculated as:

$$\mathcal{L} = \frac{\sum_{i=1}^{N} w_i \cdot \ell(x_i, y_i)}{\sum_{j=1}^{N} w_j},$$

Where w_i represents the weight of i-th nucleotide,

$$w_i = \begin{cases} 0.1 \; \textit{if position i is in repetitive region} \\ 1.0 \; \textit{otherwise} \end{cases}$$

Evolutionary constraint prediction using the zero-shot strategy

To evaluate the extent to which PlantCAD2 captures evolutionary conservation signals, a zero-shot strategy was applied to predict constrained genomic regions. Two independent tasks were used. The first task focused on *Sorghum bicolor*, using conservation estimates from the Andropogoneae tribe, a large clade of approximately 1,200 grass species that descended from a common ancestor approximately 18 million years ago.[94] To generate conservation labels, 34 high-quality genomes[95] were aligned to the Sorghum bicolor reference genome using AnchorWave.[96] Per-base conservation was quantified using alignment identity scores across all species. Sites with high-quality coverage (i.e., aligned in at least 34 out of 35 species) were retained for analysis. Among these, positions with an identity score $\geq$34 were labeled as conserved, while those with identity scores <15 were labeled as neutral. Sites with intermediate identity scores or insufficient coverage were excluded from evaluation to ensure high-confidence labels.[27]

The second task utilized conservation scores derived from multiple sequence alignments (MSAs) of orthologous coding sequences from 325 Poaceae genomes, a high-quality subset of the recently published set of 727 genomes. Using *Pharus latifolius* as an outgroup species, gap columns were removed prior to conservation estimates. Using PHAST,[97] PhyloP scores were calculated per site based on a neutral model derived from 4-fold degenerate sites and "LRT" methods with the mode "CONACC". Sites with phyloP scores above 5 were classified as conserved, while those below 1.5 were considered neutral. Sites with intermediate scores were excluded to maintain label clarity. For TIS sites, we retained all 36,668 (26,653 conserved vs. 10,015 less conserved) sites given their biological importance. For non-TIS sites, we downsampled to 183,687 sites (103,369 conserved versus 80,318 neutral) for computational efficiency while maintaining the conserved-to-neutral ratio.

For both tasks, the evaluated site was centered (i.e., 4096th of 8,192bp) within an input sequence, and the reference base at that position was masked. The model's predicted likelihood of the reference allele was extracted and used as the zero-shot conservation score. Higher likelihoods were hypothesized to reflect stronger conservation. Model performance was assessed using AUROC, comparing scores between conserved and neutral sites.

Core and non-core gene classification using the zero-shot strategy

To assess the ability of PlantCAD2 to distinguish between core and non-core genes in a population, a zero-shot strategy was applied to classify within species conservation of genes in maize and tomato. For maize, the pangene table derived from 26 Nested Association Mapping genomes[98] was used. Core genes were defined as those present in all 26 NAM genomes, whereas non-core genes included both dispensable genes (present in 2–23 genomes) and private genes (present in only one genome). For genes with multiple transcripts, the canonical transcript specified in the annotation was used. For tomato, the pan-genome dataset assembled from 586 high-quality genomes[99] was used. Genes present in all 586 accessions were defined as core genes, and non-core genes consist of dispensable (present in 6–580 accessions) and private (present in less than 5 accessions). The longest transcript was selected to represent each gene across all analyses.

To quantify the model's prediction at each functional junction, a masked motif accuracy score was defined. For example, to evaluate translation initiation sites, the canonical ATG start codon was masked, and the model's predicted likelihoods for the three masked nucleotides were extracted and averaged. A similar approach was applied to other junction types, including translation termination sites (TAA, TAG, TGA), splice donor sites (GT), and splice acceptor sites (AG), by masking the corresponding motifs and calculating average token likelihoods.

To evaluate performance, core genes were treated as positive and non-core genes as negative, and AUROC was calculated based on the masked motif accuracy scores for each gene.

Accessible chromatin region prediction

To evaluate the capability of PlantCAD2 to capture regulatory sequence features, fine-tuning experiments were performed using Low-Rank Adaptation (LoRA)[67] on two accessible chromatin prediction tasks: (1) cross-species accessible regions (ACRs) prediction, and (2) cell-type-specific ACR prediction.

For the cross-species task, ATAC-seq peak regions from 12 plant species[66] were downloaded from NCBI (https://www.ncbi.nlm.nih.gov/geo/query/acc.cgi?acc=GSE128434). We followed the data processing pipeline as described by Wrightsman et al.[100] For each species, peak regions were processed by extracting the midpoint of each peak and symmetrically extending it by half the target input window size (300bp, 600bp and 1,000bp) in both directions to generate positive observations. To reflect the real-world scenario in which most of the genome is inaccessible, the rest of the genome was used as negative examples, ensuring no overlap with known peaks.

For the cell-type-specific task, we used the single-cell ATAC-Seq[70] and used a similar preprocessing pipeline, but each genomic region could be associated with accessibility across 92 cell types. As such, the task was framed as a multi-label classification problem, where each region was assigned a binary accessibility label for each of the 92 cell types based on its overlap with experimentally identified peaks.

Gene expression prediction in leaf

To evaluate the models' ability to predict gene expression, we designed two tasks: (1) leaf absolute expression and (2) leaf on/off expression classification. The training dataset was derived from 15 Andropogoneae species.[71] For validation, we held out two species closest to *Zea mays*—*Tripsacum zopilotense* and *Zea diploperennis*—both members of the Tripsacinae subtribe, which diverged from maize approximately 0.6 to 4 million years ago. This setup enabled evaluation of the models' cross-species generalization to closely related taxa. For the leaf absolute expression task, the $\log_{10}$TPM values were used as regression targets during fine-tuning. For the on/off expression task, genes with TPM >1 were labeled as expressed (positive), and those with TPM $\leq$1 were considered non-expressed (negative). This setup enabled evaluation of the models' ability to generalize expression predictions across closely related species within the clade.

Leaf protein abundance prediction task

To evaluate the models' ability to predict protein abundance, we designed two tasks analogous to the gene expression analysis: absolute abundance and on/off classification. Ribo-Seq data were obtained from Arabidopsis[101] and *Z. mays*.[102] Raw reads were downloaded from NCBI, and Trimmomatic[103] was used to trim adapters and filter low-quality reads. Cleaned reads were first aligned to rRNA reference sequences using Bowtie[104] to remove contaminating rRNA. The remaining reads were then mapped to the reference genomes of Arabidopsis and maize using STAR.[105] Gene-level translation abundance was quantified using StringTie[106] based on uniquely mapped reads. We then designed two tasks analogous to the expression prediction setup: (1) absolute protein abundance, where the log10-transformed Ribo-Seq expression values were used as regression targets, and (2) on/off classification, where genes with TPM >1 were considered expressed (positive) and those with TPM $\leq$1 were labeled as non-expressed (negative).

Fine-tuning PlantCAD2

To adapt the pre-trained PlantCAD2 model to downstream tasks, we employed Low-Rank Adaptation (LoRA),[67] a parameter-efficient fine-tuning strategy that inserts trainable low-rank matrices into the attention layers of the transformer. This approach enables effective adaptation while keeping the vast majority of model parameters frozen. Fine-tuning was performed using the PEFT library[107] with LoRA rank = 8, α = 32, and dropout = 0.1, targeting the "x_proj", "in_proj", and "out_proj" modules. Models were trained using the Hugging Face Trainer with a learning rate of 1e−4, a global batch size of 128, and one training epoch. BF16 precision and linear learning rate scheduling with 50 warm-up steps were used. Over 98% of the model parameters remained frozen, enabling efficient and scalable fine-tuning across tasks. All tasks were fine-tuned for a single epoch without hyperparameter tuning to ensure stability and consistency across experiments. Fine-tuning objectives for all models were a binary cross entropy loss for classification tasks and a mean squared error loss for regression tasks.

Fine-tuning AgroNT

To directly compare the performance of fine-tuned PlantCAD2 with AgroNT,[24] we applied the same parameter-efficient fine-tuning strategy using LoRA. All LoRA hyperparameters were kept consistent with those used for PlantCAD2, including rank = 8, α = 32, and

dropout = 0.1. For AgroNT, LoRA adapters were inserted into the "query" and "key" projection layers of the transformer,[42] reflecting its architecture. This setup ensured a fair comparison between models under matched fine-tuning conditions.

Supervised CNN + LSTM baseline

To benchmark against traditional supervised models, we implemented a CNN+LSTM architecture based on DanQ,[68] a widely used hybrid model for DNA sequence classification. For each task, the model was trained from scratch using one-hot encoded sequences. We used the Adam optimizer with a learning rate of 0.01, a batch size of 2,048, and trained for up to 200 epochs, with early stopping after 20 epochs without validation improvement.

Supervised PlantCAD2 baseline

To assess the contribution of pretraining, we trained a small PlantCAD2 model from scratch for each downstream task. This supervised baseline used the same architecture and hyperparameters as the fine-tuned version but was initialized without pretrained weights—by loading only the Hugging Face model configuration.

Zero-shot evaluation of PlantCAD2, PlantCAD, GPN, and NTv3 models

All three models were pre-trained with masked language modeling. For PlantCAD, we used the largest available model "kuleshov-group/PlantCaduceus_l32″ for evaluation. For GPN, we used "songlab/gpn-brassicales"". Due to the 512 bp context window limitation of both PlantCAD and GPN, we cropped input sequences to 512 bp centered on the target position. For NTv3, we used the largest pre-trained version (InstaDeepAI/NTv3_650M_pre) for evaluation. All other evaluation configurations remained identical to those used for PlantCAD2.

Zero-shot evaluation of Evo2 model

All zero-shot tasks were also benchmarked using the Evo2[19] model ("evo2_7b") for comparison. Since Evo2 is autoregressive (predicting the next token rather than masked tokens), masked token accuracy could not be directly computed. Therefore, for the evolutionary constraint task, we fed the full input sequence into the model and extracted the likelihood of the reference allele at the target site as the conservation score. To ensure a fair comparison, we used an 8,192 bp context window for Evo2, matching the input length used for PlantCAD2 evaluations. The same approach was applied for benchmarking structural variants.

For the masked motif accuracy task, we evaluated Evo2 using two configurations to compensate for its unidirectional architecture: (1) forward sequences (Evo2-fwd), where the model uses upstream context to predict the junction—for example, for TIS prediction, we used the 4,094 bp upstream of the TIS as a prompt for Evo2 to generate the next three tokens; and (2) reverse-complement sequences (Evo2-rc), where the model uses downstream context (reverse complemented) to predict the junction in the opposite direction.

QUANTIFICATION AND STATISTICAL ANALYSIS

No statistical hypothesis tests were performed; all model comparisons are based on held-out test set performance metrics (AUROC, AUPRC, Spearman correlation) with sample sizes reported in Tables 1, 2, S3, S4, S5, S6, S7, S8, S9, S10, and S11.

ADDITIONAL RESOURCES

This study did not generate additional resources.

Cell Genomics, Volume *6*

Supplemental information

PlantCAD2: A DNA foundation model

for interpreting genomes across flowering plants

Jingjing Zhai, Aaron Gokaslan, Sheng-Kai Hsu, Szu-Ping Chen, Zong-Yan Liu, Edgar Marroquin, Eric Czech, Betsy Cannon, Ana Berthel, M. Cinta Romay, Matt Pennell, Volodymyr Kuleshov, and Edward S. Buckler

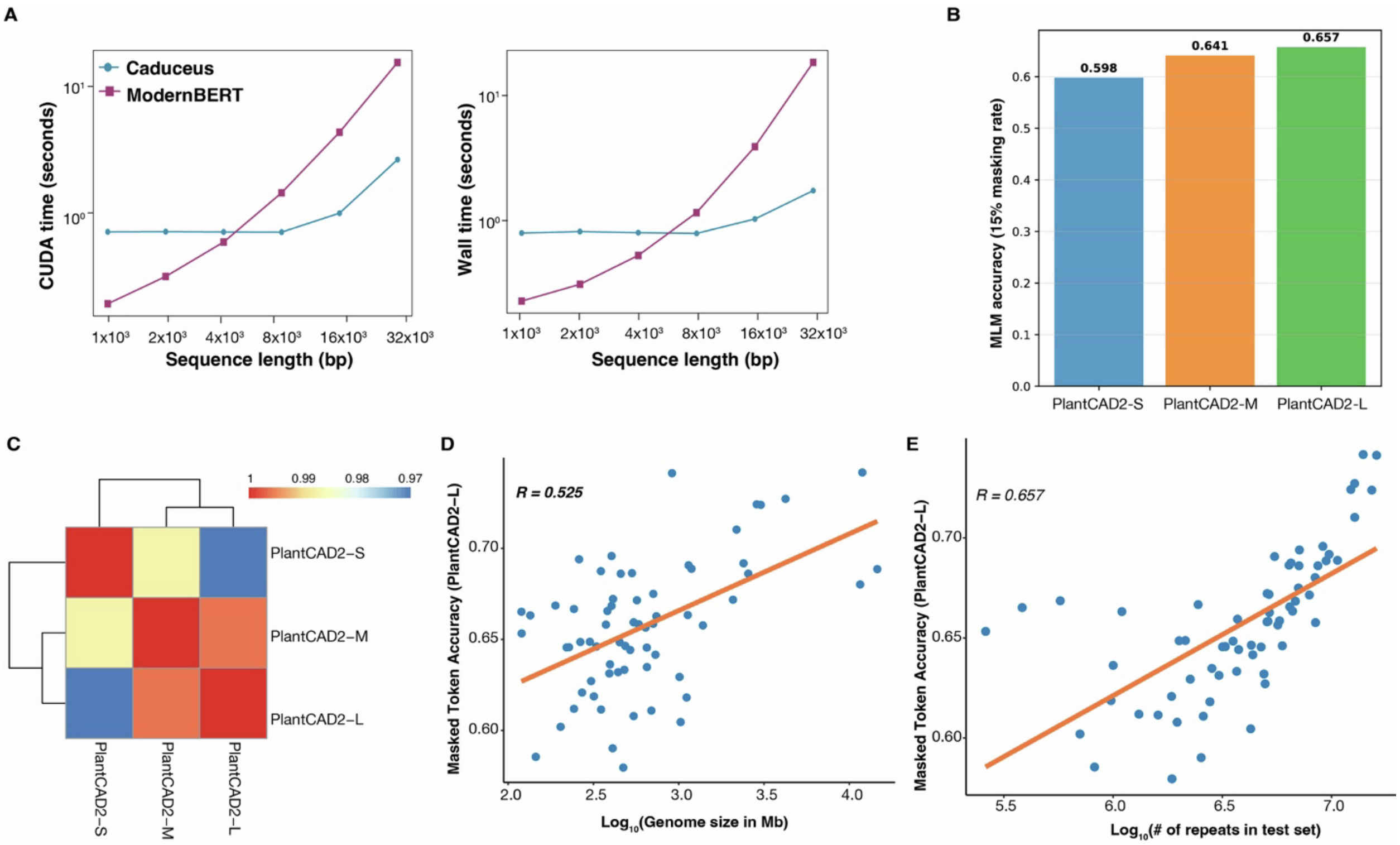

Figure S1. PlantCAD2 model performance across different scales and species, related to Figure 1. (A) Inference speed benchmarking shows that the Caduceus architecture exhibits significantly faster CUDA and wall-clock runtime than ModernBERT across increasing sequence lengths. **(B)** Masked language modeling accuracy across the three models, evaluated by randomly masking 15% of nucleotides per sequence, showing improved performance with increasing model size. **(C)** Correlation matrix of per-species masked language modeling accuracy of three PlantCAD2 models. **(D)** Relationship between genome size and masked language modeling accuracy for PlantCAD2-L. **(E)** Relationship between the number of repetitive sequences in the test set and masked language modeling accuracy.

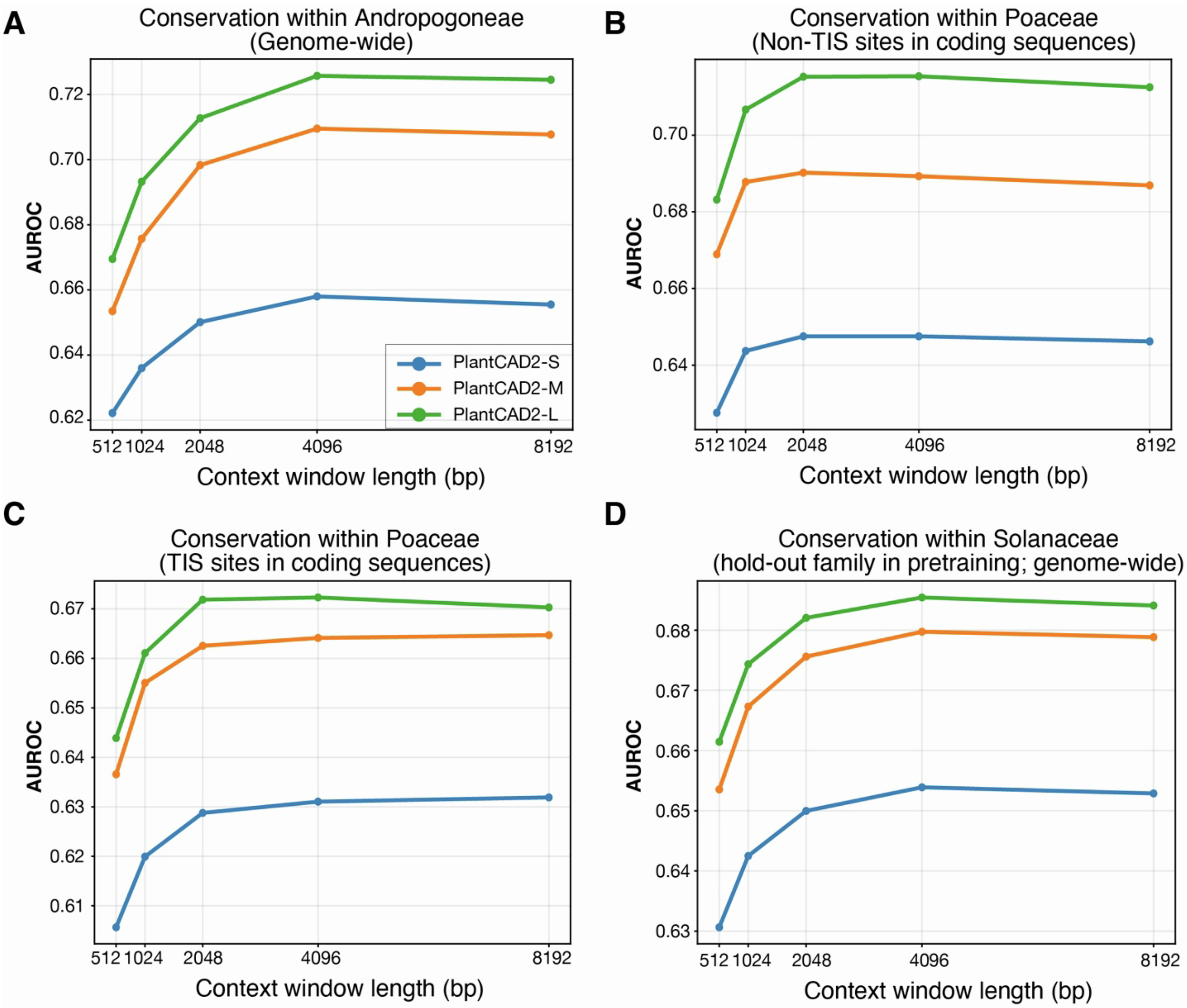

Figure S2. Effect of context window length on AUROC performance, related to Figure 2. Evaluation across three PlantCAD2 models for conservation within Andropogoneae **(A)**, Poaceae non-TIS **(B)**, Poaceae TIS **(C)**, and Solanaceae **(D)**.

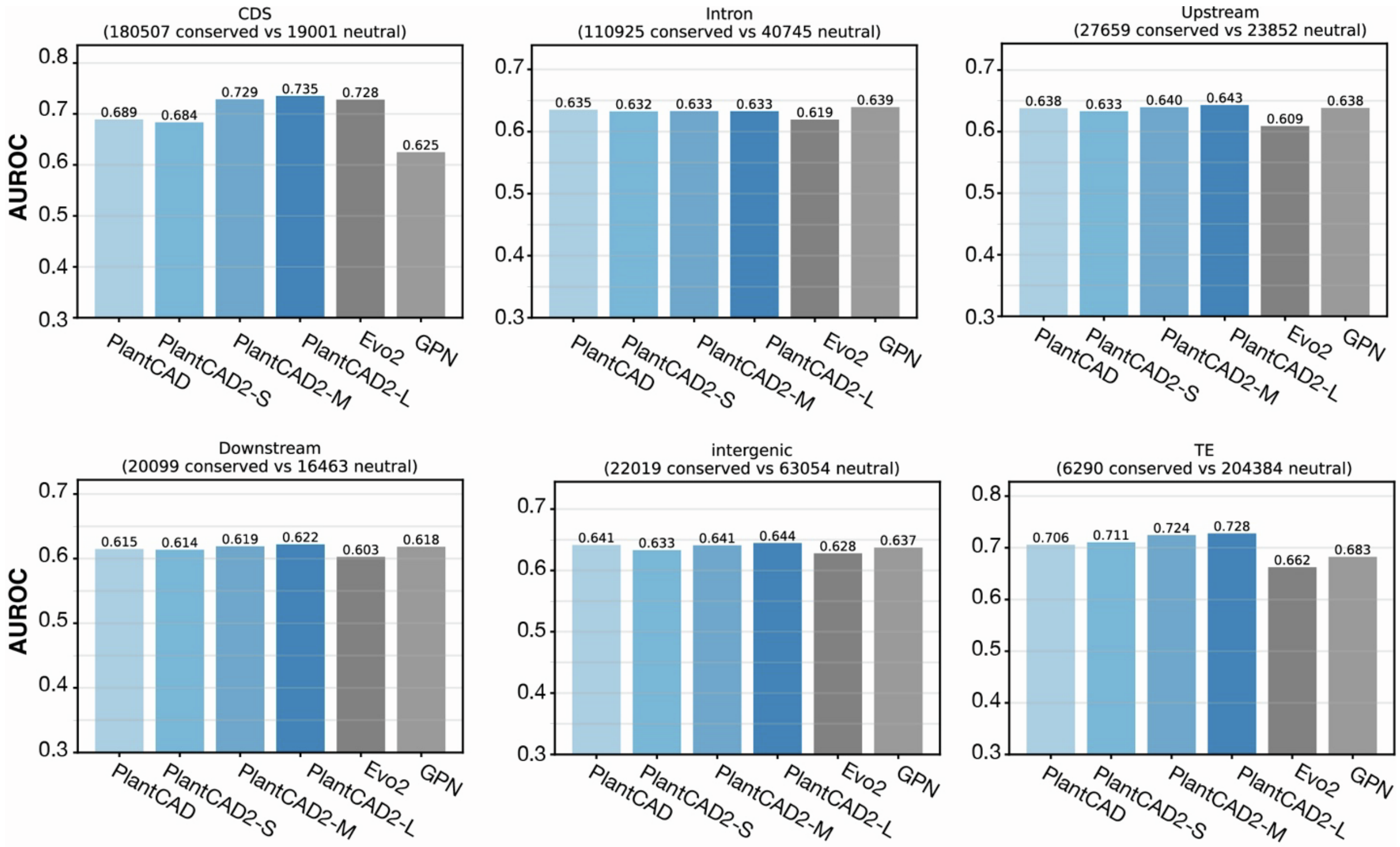

Figure S3. AUROC of different genomic contexts for conservation prediction in Solanaceae, related to Figure 2.

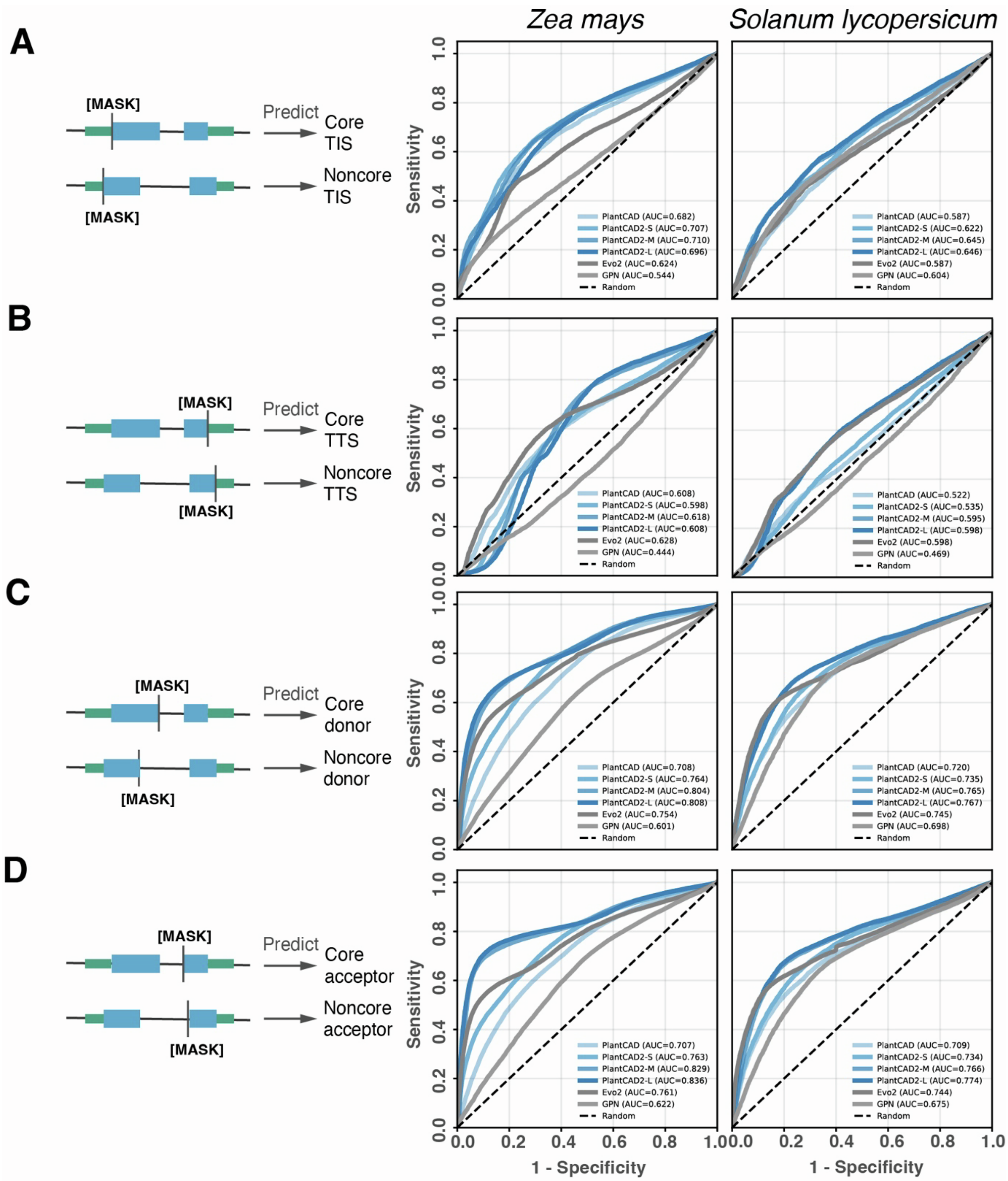

Figure S4. PlantCAD2 distinguishes core from non-core junctions with zero-shot strategy, related to Figure 3. ROC curves showing binary classification performance for distinguishing evolutionarily constrained core junctions from rapidly evolving non-core junctions using model log-likelihood scores at junction sites. Left panels show maize (included in pre-training), right panels show tomato (excluded from training). **(A)** Translation initiation sites. **(B)** Translation termination sites. **(C)** Splice donor sites. **(D)** Splice acceptor sites.

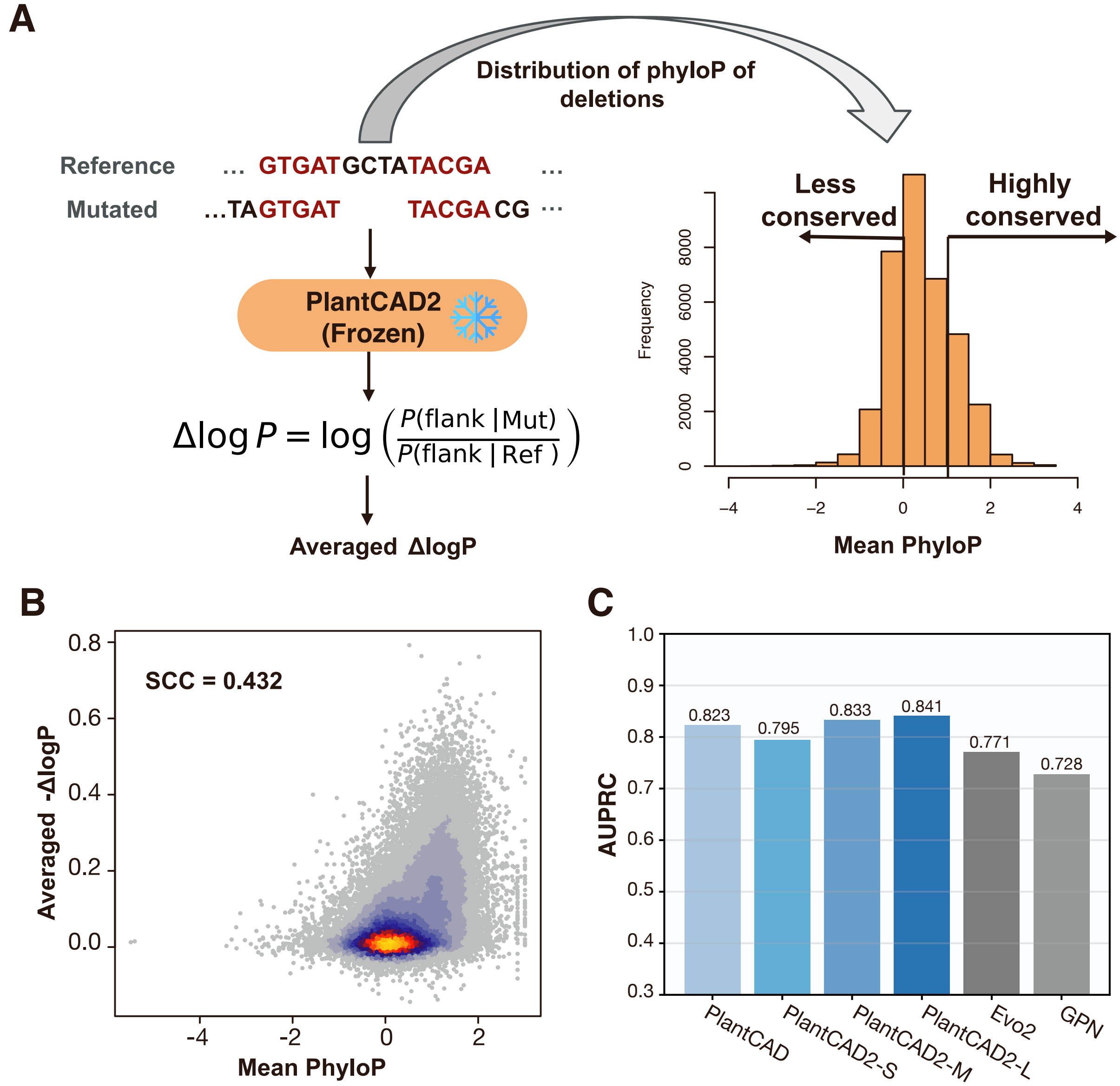

Figure S5. PlantCAD2 predicts functional impact of structural variants using zero-shot strategy, related to Table 1. (A) ΔlogP calculation approach for deletion variants and phyloP score distribution for classification. **(B)** Scatter plot showing the positive correlation between PlantCAD2's -ΔlogP scores and phyloP-based conservation scores. **(C)** AUROC performance distinguishes highly conserved from less conserved deletions.

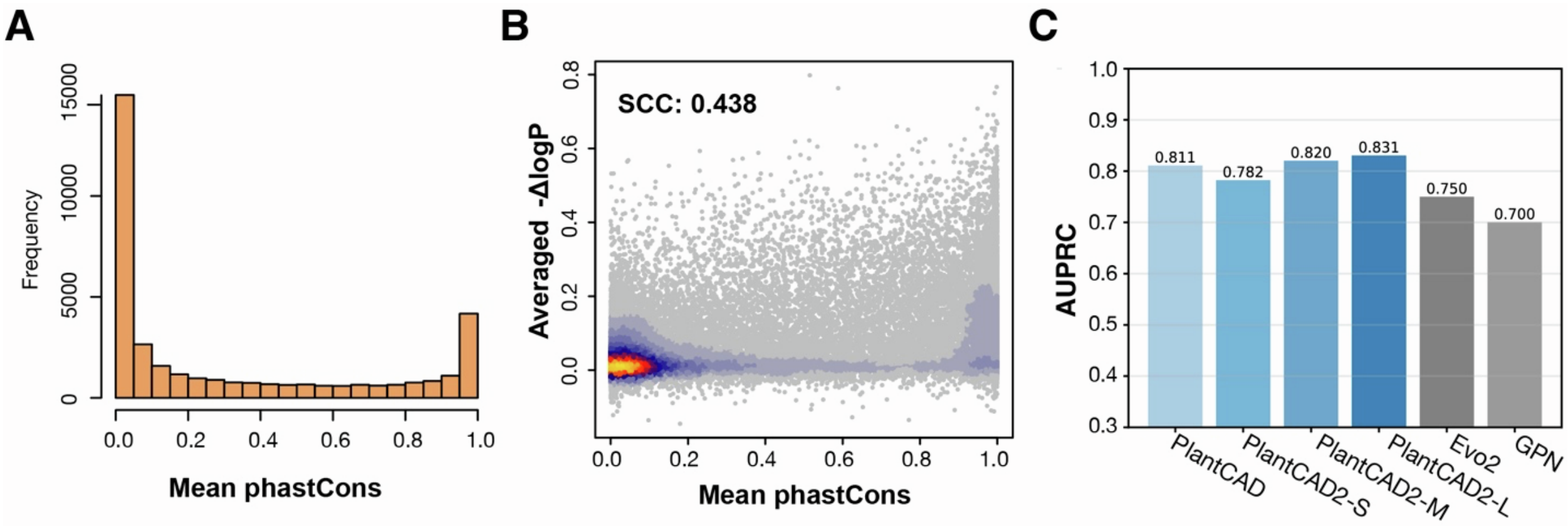

Figure S6. PlantCAD2 predicts functional impact of structural variants using phastCons scores, related to Table 1. (A) Distribution of phastCons. (B) Scatter plot showing the positive correlation between PlantCAD2's -ΔlogP scores and phyloP-based conservation scores. (C) AUROC performance distinguishes highly conserved from less conserved deletions.

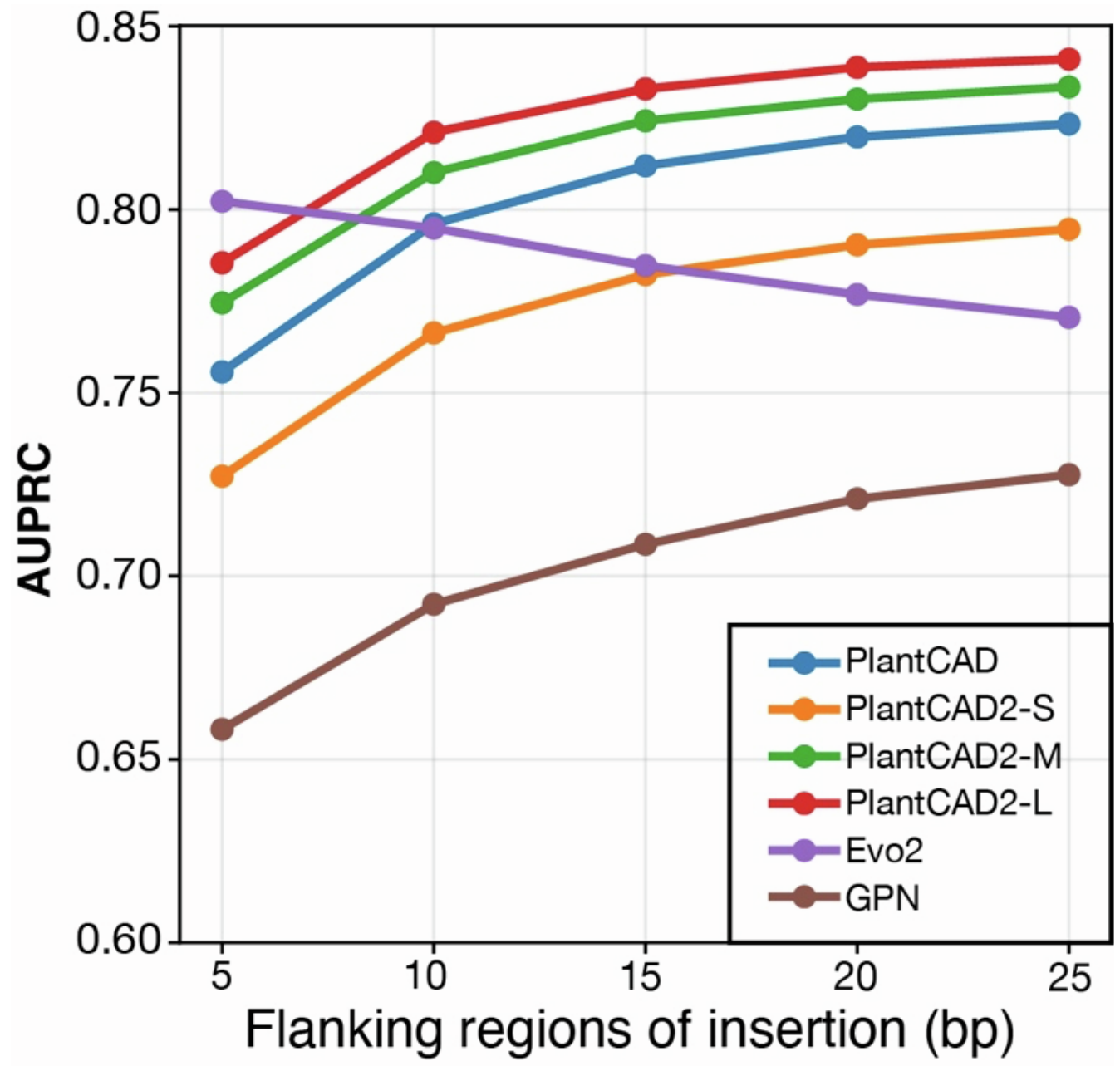

Figure S7. Effect of flanking sequence length on deletion classification performance, related to Table 1. AUROC performance for distinguishing conserved from less conserved deletions by flanking sequence length.

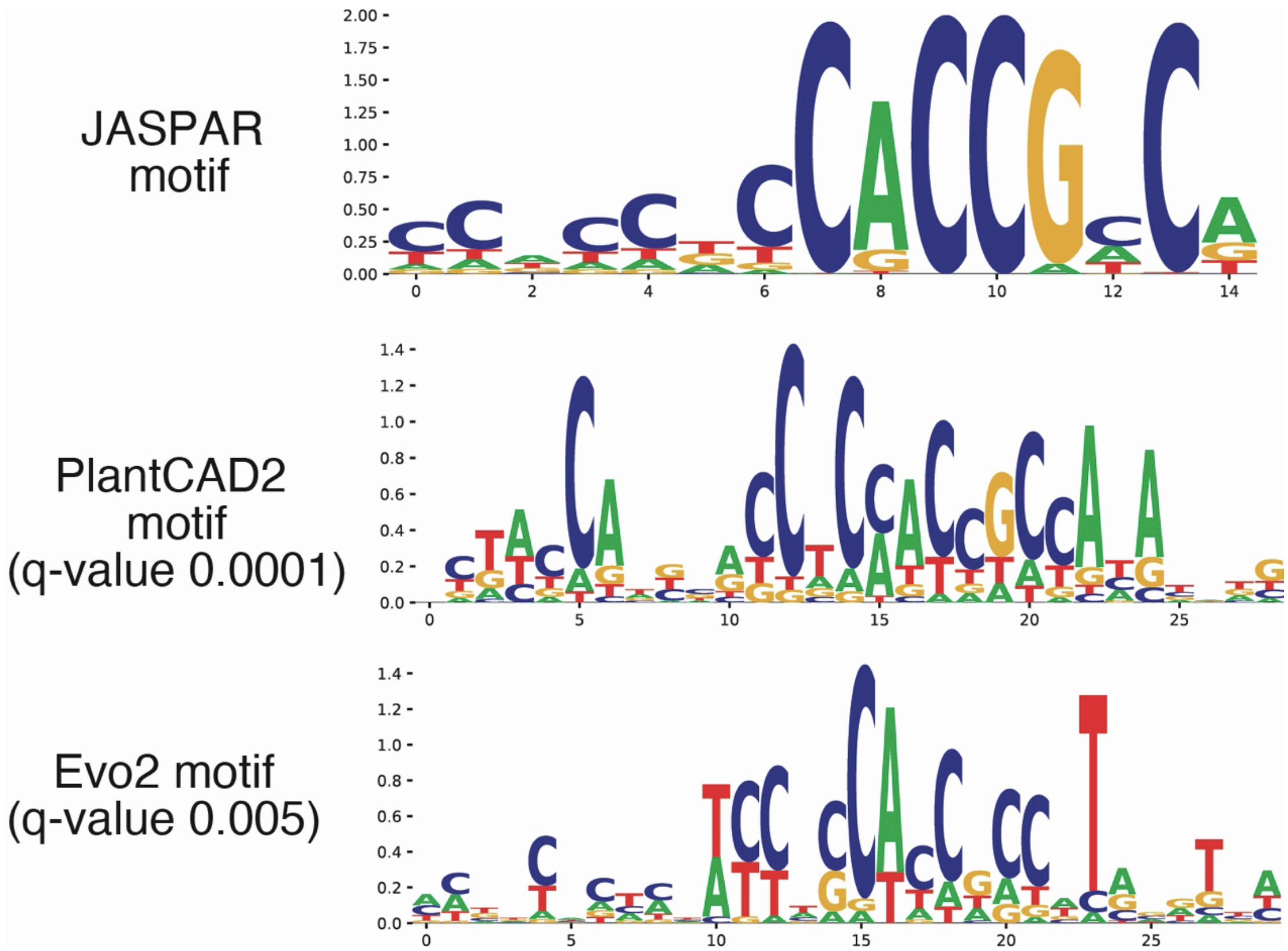

Figure S8. Motifs generated by TF-MoDISco from PlantCAD2 and Evo2 high-confidence predictions for DREB2 (AP2/ERF family), related to STAR Methods.

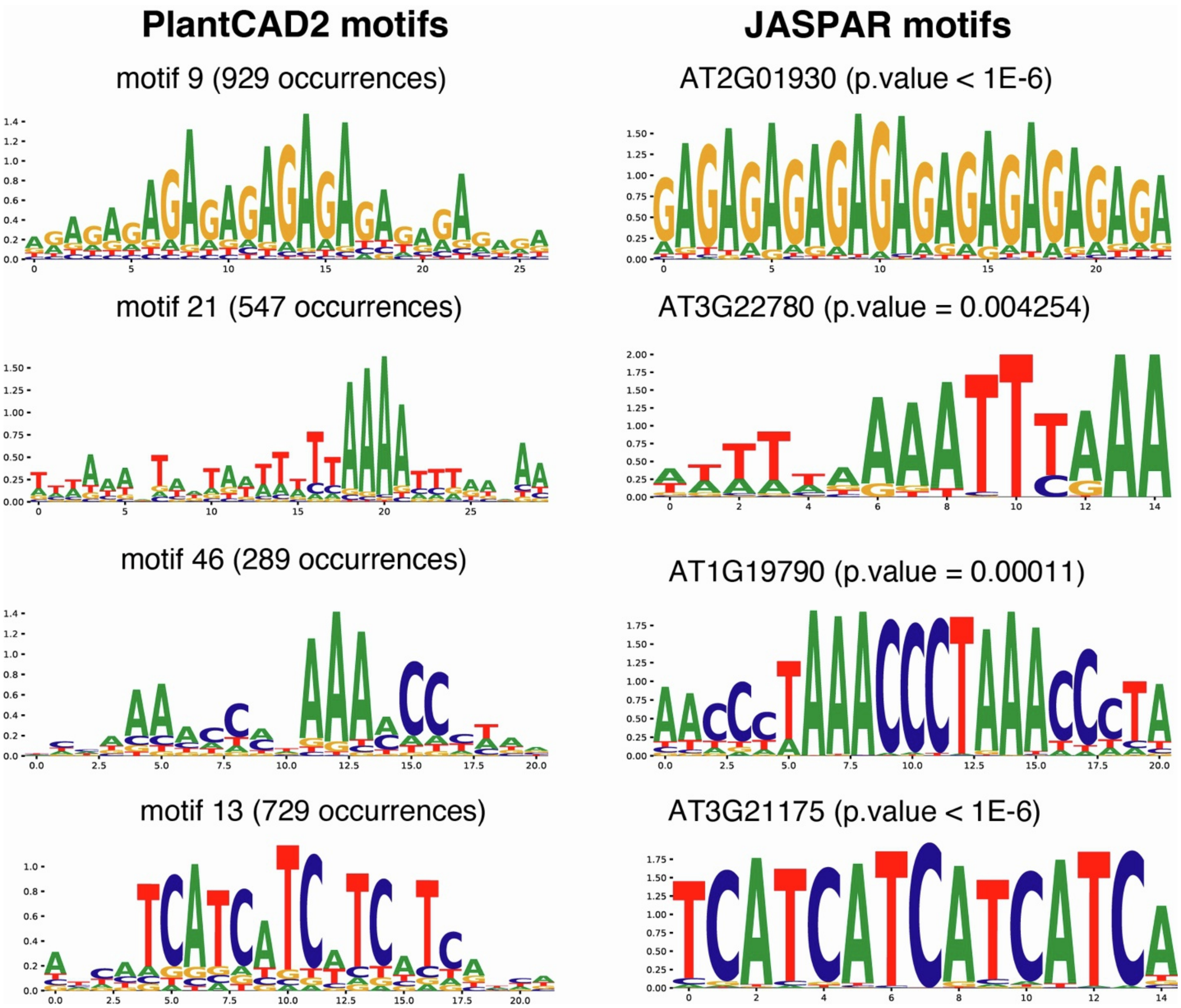

Figure S9. Example motifs generated by TF-MoDISco from PlantCAD2 high-confidence predictions in Arabidopsis promoter regions, related to STAR Methods.

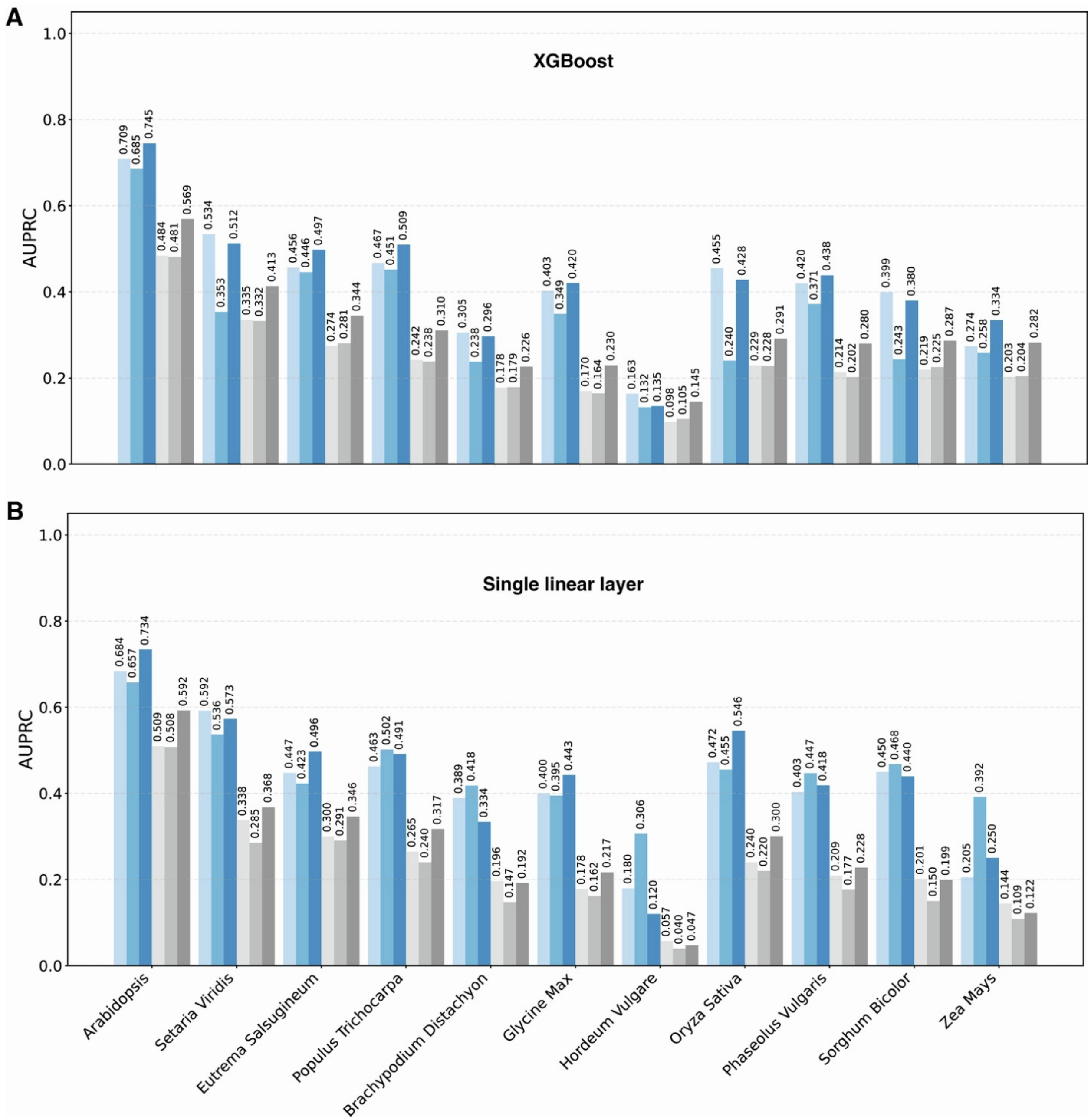

Figure S10. Accessible region prediction task comparison with Evo2 using frozen embeddings, related to Figure 4. (A) XGBoost classifier**, (B)** neural network with single linear layer.

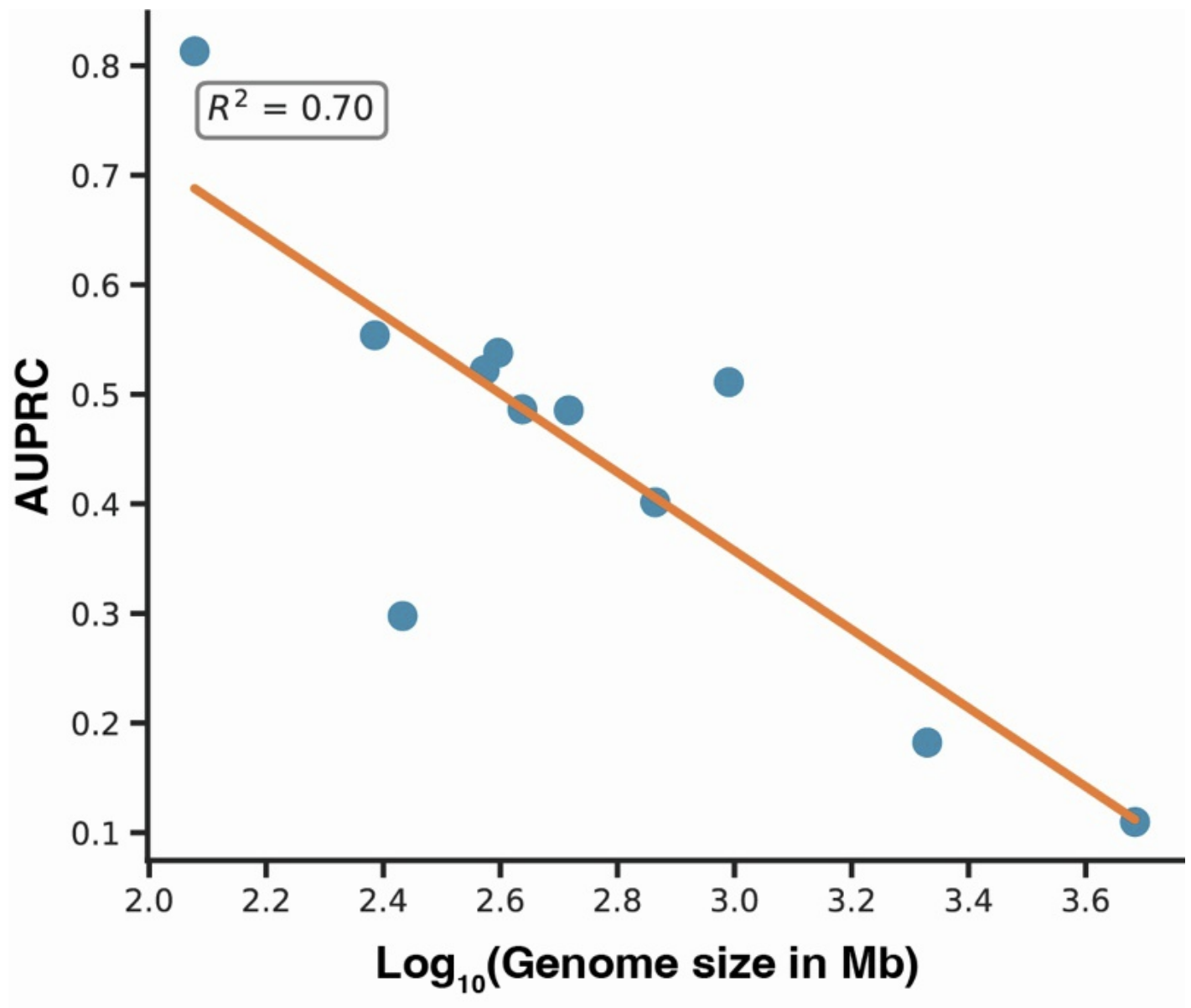

Figure S11. Relationship between genome size and accessibility prediction performance, related to Figure 4.

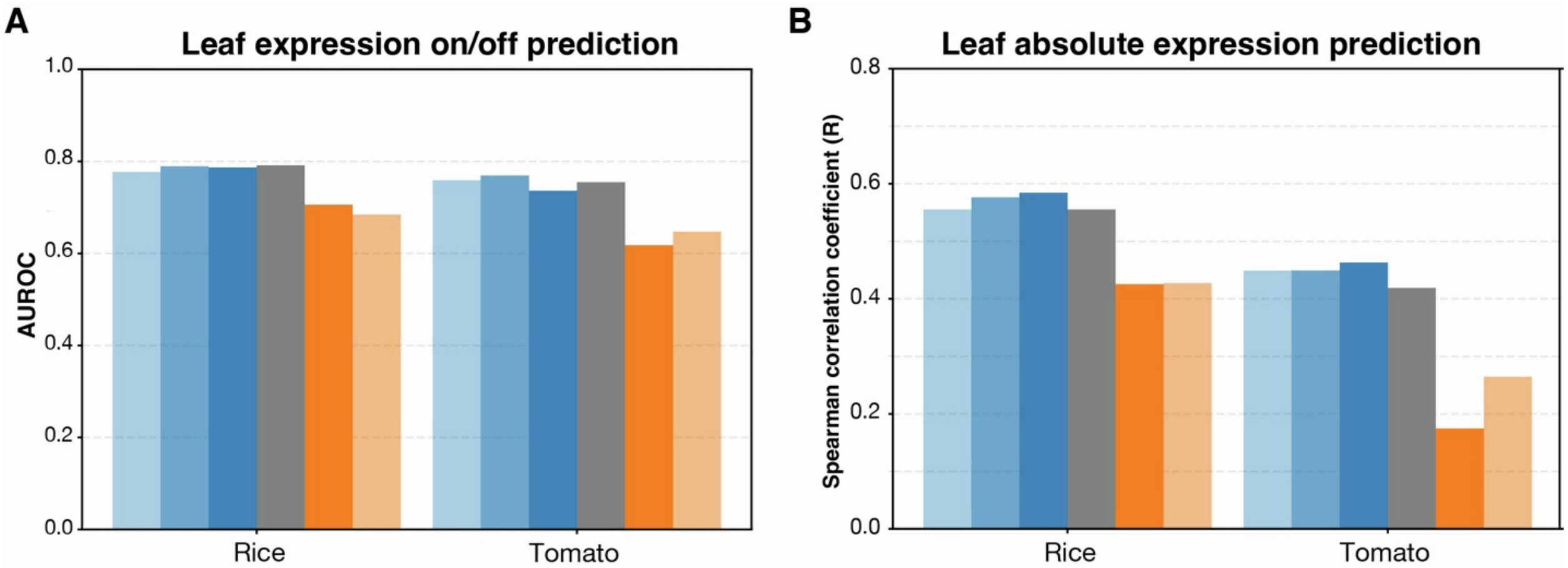

Figure S12. Gene expression prediction performance in rice and tomato, related to Figure 5. **(A)** Leaf expression on/off prediction**, (B)** leaf absolute expression prediction.

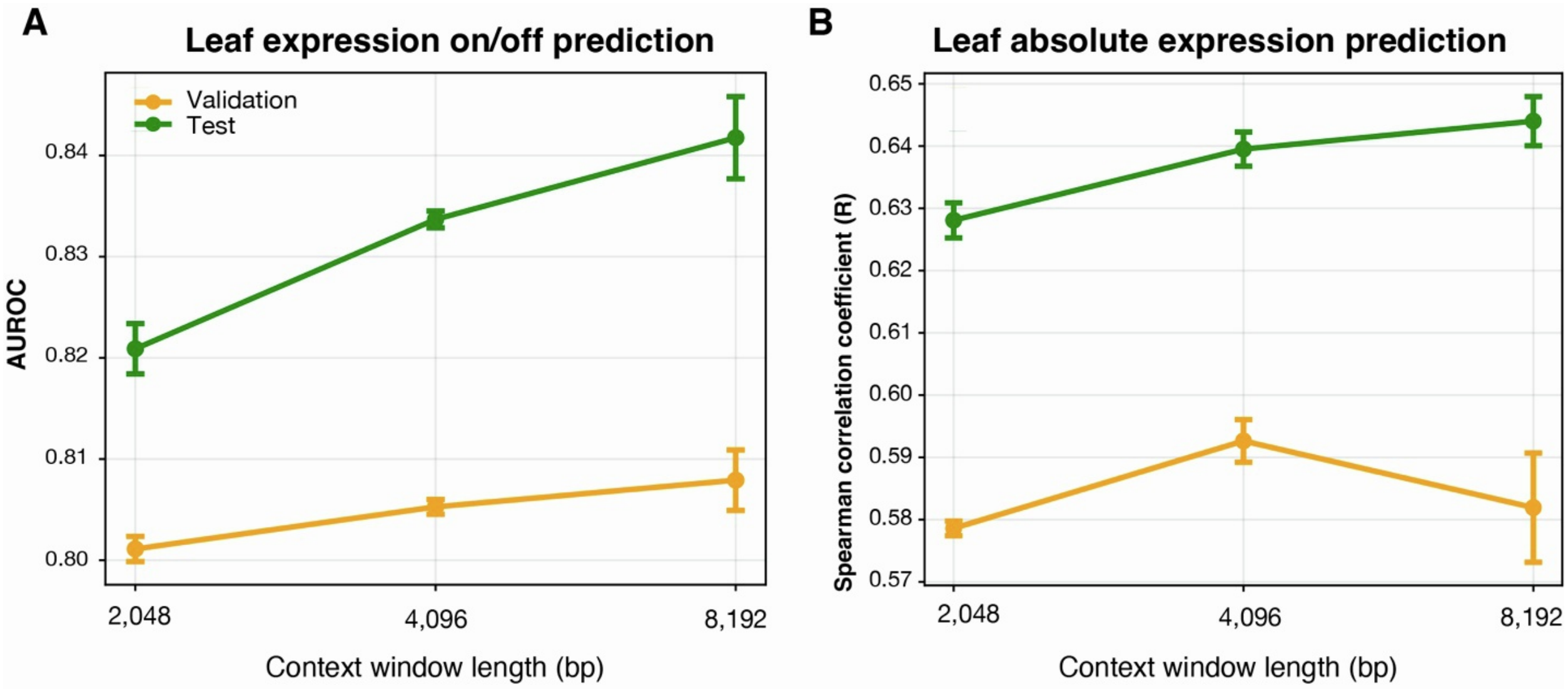

Figure S13. Effect of context window length on gene expression prediction performance, related to Figure 5. Performance improvement with increasing context window size for binary leaf expression classification **(A)** and absolute expression regression **(B)** on the maize NAM dataset. Error bars represent five replicates with different random seeds.

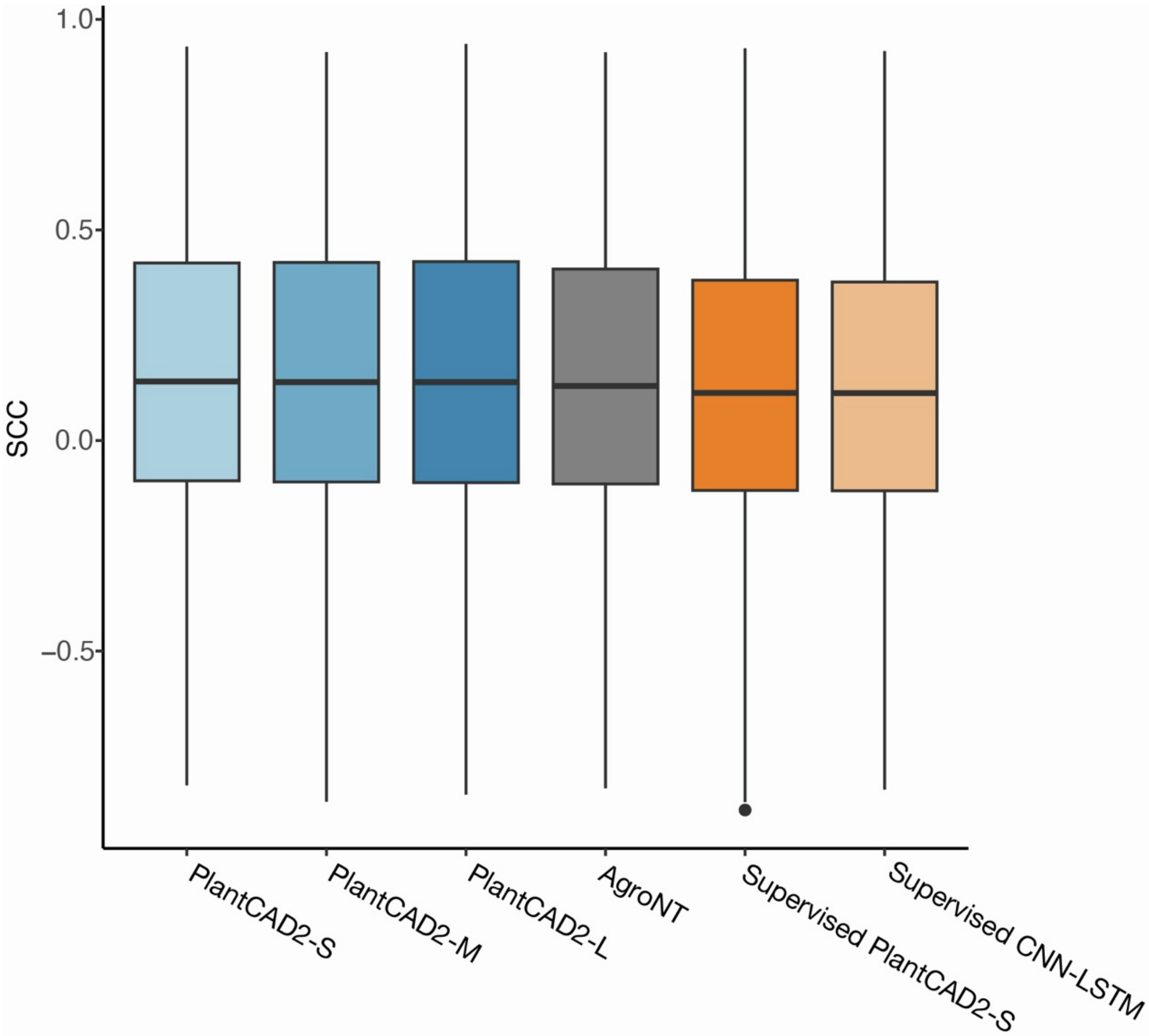

Figure S14. Per-orthogroup expression correlation performance within the maize NAM population, related to Figure 5. Spearman correlation coefficients (SCC) for predicting allele-specific expression differences within orthogroups.

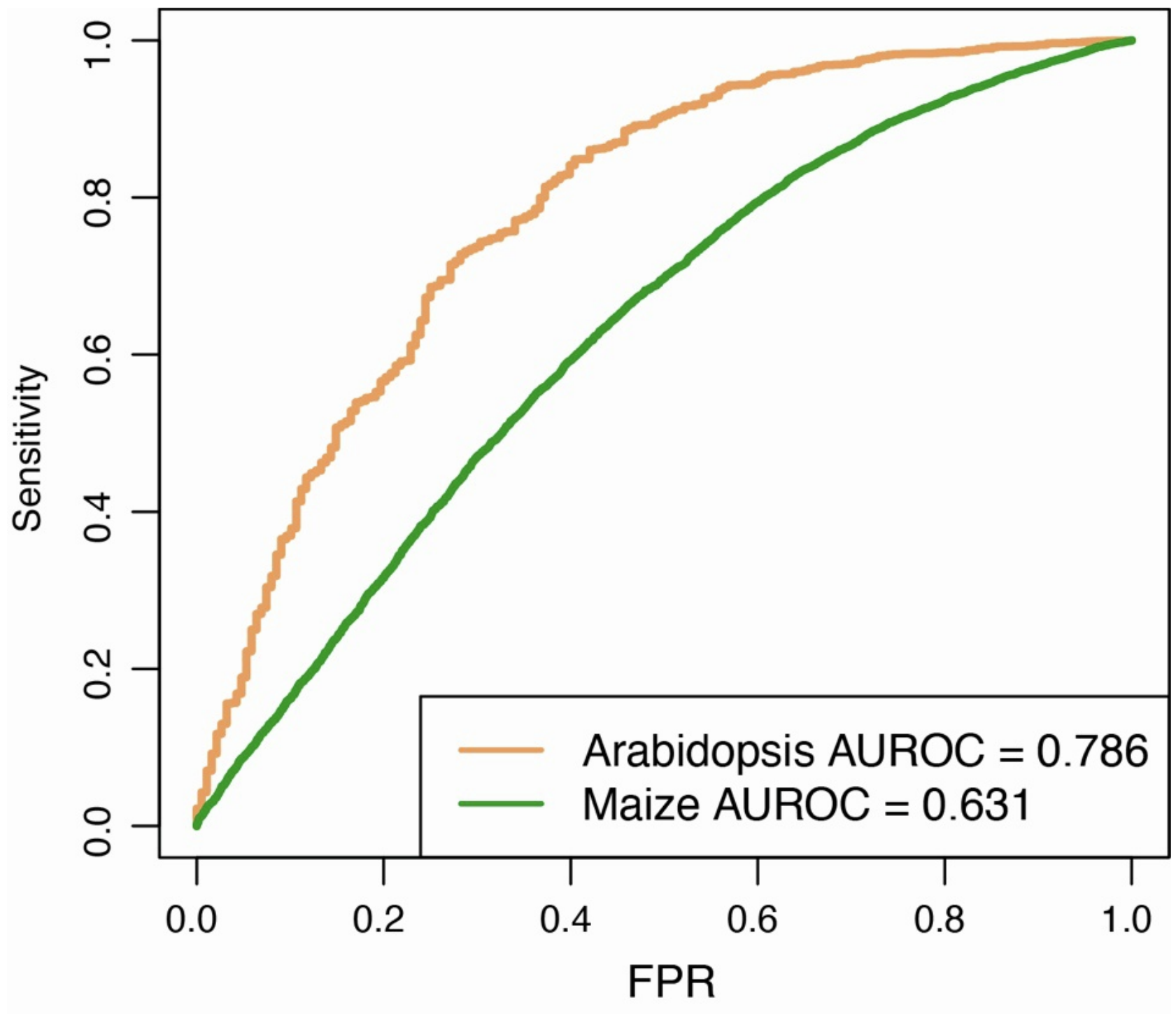

Figure S15. Cross-species transfer performance for gene expression prediction, related to Figure 5. ROC curves showing performance when training on Arabidopsis and testing cross-species transfer to maize.

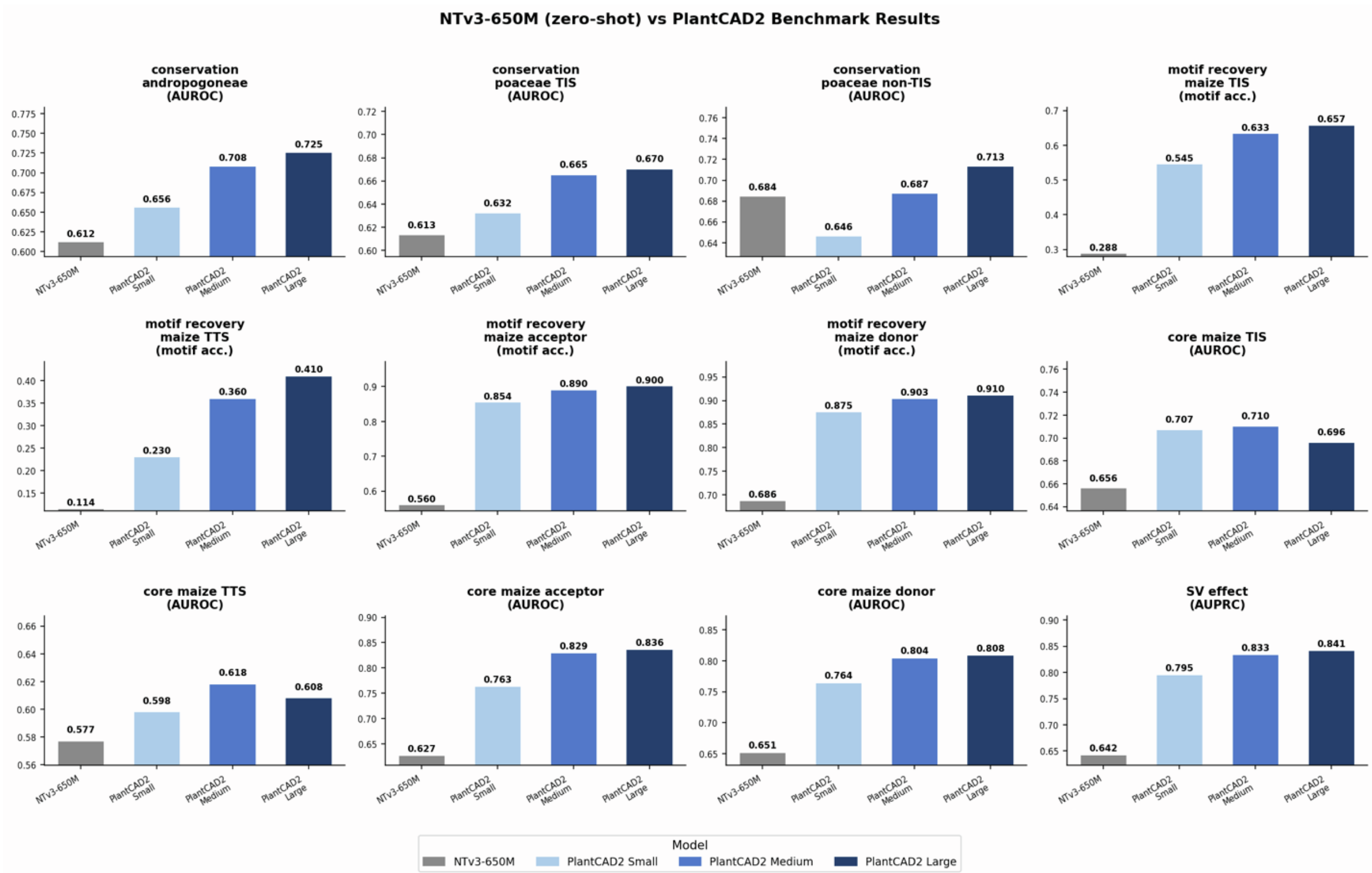

Figure S16. Zero-shot performance comparison between PlantCAD2 and NTv3, related to Table 1.

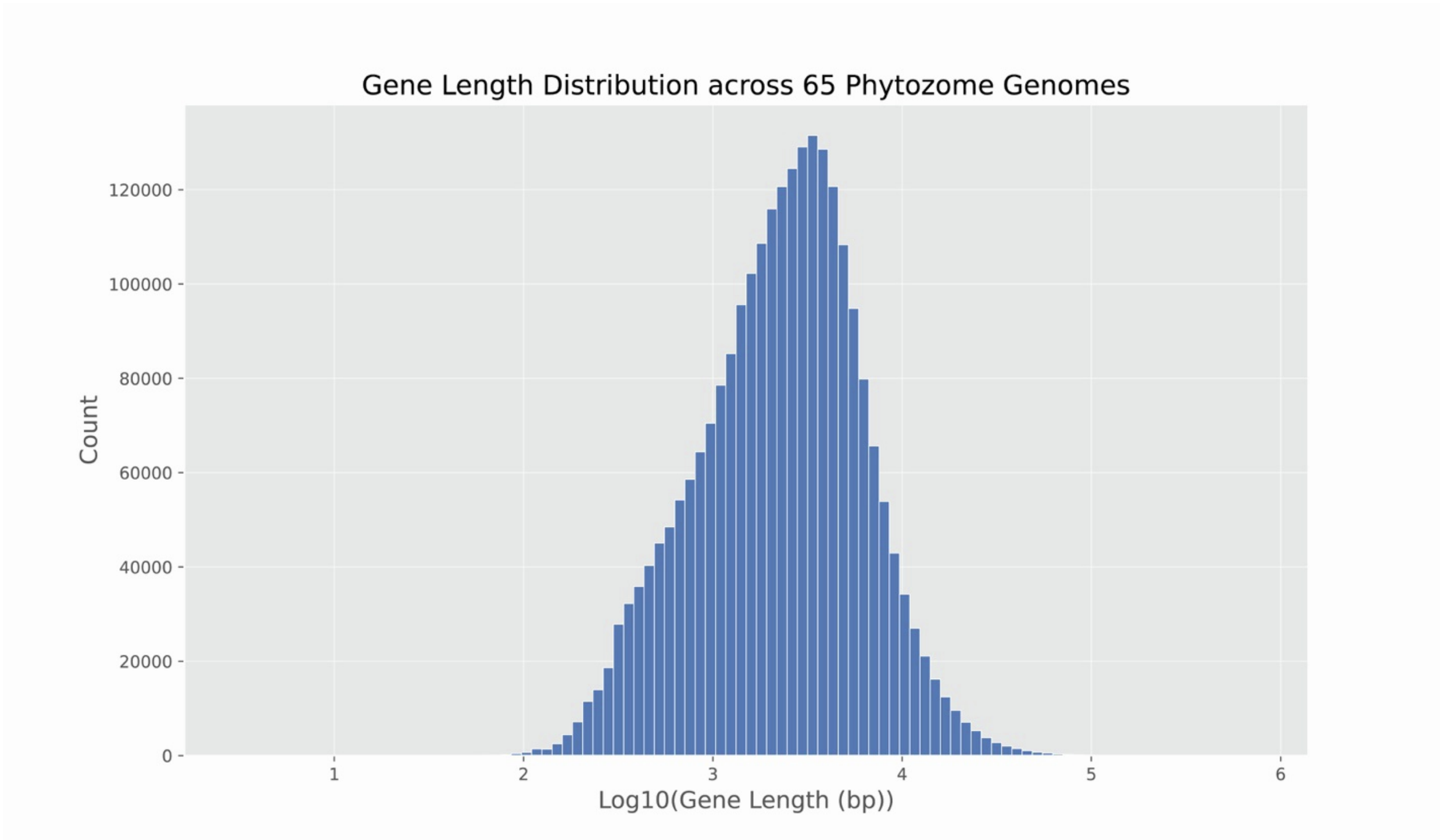

Figure S17. Gene length distribution of 65 pretraining genomes, related to STAR Methods.
